# Comparative Genomics of Plant-Associated *Pseudomonas* spp.: Insights into Diversity and Inheritance of Traits Involved in Multitrophic Interactions

**DOI:** 10.1371/journal.pgen.1002784

**Published:** 2012-07-05

**Authors:** Joyce E. Loper, Karl A. Hassan, Dmitri V. Mavrodi, Edward W. Davis, Chee Kent Lim, Brenda T. Shaffer, Liam D. H. Elbourne, Virginia O. Stockwell, Sierra L. Hartney, Katy Breakwell, Marcella D. Henkels, Sasha G. Tetu, Lorena I. Rangel, Teresa A. Kidarsa, Neil L. Wilson, Judith E. van de Mortel, Chunxu Song, Rachel Blumhagen, Diana Radune, Jessica B. Hostetler, Lauren M. Brinkac, A. Scott Durkin, Daniel A. Kluepfel, W. Patrick Wechter, Anne J. Anderson, Young Cheol Kim, Leland S. Pierson, Elizabeth A. Pierson, Steven E. Lindow, Donald Y. Kobayashi, Jos M. Raaijmakers, David M. Weller, Linda S. Thomashow, Andrew E. Allen, Ian T. Paulsen

**Affiliations:** 1 Agricultural Research Service, U.S. Department of Agriculture, Corvallis, Oregon, United States of America; 2 Department of Botany and Plant Pathology, Oregon State University, Corvallis, Oregon, United States of America; 3 Department of Chemistry and Biomolecular Sciences, Macquarie University, Sydney, Australia; 4 Department of Plant Pathology, Washington State University, Pullman, Washington, United States of America; 5 Laboratory of Phytopathology, Wageningen University, Wageningen, The Netherlands; 6 The J. Craig Venter Institute, Rockville, Maryland, United States of America; 7 Agricultural Research Service, U.S. Department of Agriculture, Davis, California, United States of America; 8 Agricultural Research Service, U.S. Department of Agriculture, Charleston, South Carolina, United States of America; 9 Department of Biology, Utah State University, Logan, Utah, United States of America; 10 Institute of Environmentally-Friendly Agriculture, Chonnam National University, Gwangju, Korea; 11 Department of Plant Pathology and Microbiology, Texas A&M University, College Station, Texas, United States of America; 12 Department of Horticultural Sciences, Texas A&M University, College Station, Texas, United States of America; 13 Department of Plant and Microbial Biology, University of California Berkeley, Berkeley, California, United States of America; 14 Department of Plant Biology and Pathology, Rutgers, The State University of New Jersey, New Brunswick, New Jersey, United States of America; 15 Agricultural Research Service, U.S. Department of Agriculture, Pullman, Washington, United States of America; 16 The J. Craig Venter Institute, San Diego, California, United States of America; University of Toronto, Canada

## Abstract

We provide here a comparative genome analysis of ten strains within the *Pseudomonas fluorescens* group including seven new genomic sequences. These strains exhibit a diverse spectrum of traits involved in biological control and other multitrophic interactions with plants, microbes, and insects. Multilocus sequence analysis placed the strains in three sub-clades, which was reinforced by high levels of synteny, size of core genomes, and relatedness of orthologous genes between strains within a sub-clade. The heterogeneity of the *P. fluorescens* group was reflected in the large size of its pan-genome, which makes up approximately 54% of the pan-genome of the genus as a whole, and a core genome representing only 45–52% of the genome of any individual strain. We discovered genes for traits that were not known previously in the strains, including genes for the biosynthesis of the siderophores achromobactin and pseudomonine and the antibiotic 2-hexyl-5-propyl-alkylresorcinol; novel bacteriocins; type II, III, and VI secretion systems; and insect toxins. Certain gene clusters, such as those for two type III secretion systems, are present only in specific sub-clades, suggesting vertical inheritance. Almost all of the genes associated with multitrophic interactions map to genomic regions present in only a subset of the strains or unique to a specific strain. To explore the evolutionary origin of these genes, we mapped their distributions relative to the locations of mobile genetic elements and repetitive extragenic palindromic (REP) elements in each genome. The mobile genetic elements and many strain-specific genes fall into regions devoid of REP elements (i.e., REP deserts) and regions displaying atypical tri-nucleotide composition, possibly indicating relatively recent acquisition of these loci. Collectively, the results of this study highlight the enormous heterogeneity of the *P. fluorescens* group and the importance of the variable genome in tailoring individual strains to their specific lifestyles and functional repertoire.

## Author Summary

We sequenced the genomes of seven strains of the *Pseudomonas fluorescens* group that colonize plant surfaces and function as biological control agents, protecting plants from disease. In this study, we demonstrated the genomic diversity of the group by comparing these strains to each other and to three other strains that were sequenced previously. Only about half of the genes in each strain are present in all of the other strains, and each strain has hundreds of unique genes that are not present in the other genomes. We mapped the genes that contribute to biological control in each genome and found that most of the biological control genes are in the variable regions of the genome, which are not shared by all of the other strains. This finding is consistent with our knowledge of the distinctive biology of each strain. Finally, we looked for new genes that are likely to confer antimicrobial traits needed to suppress plant pathogens, but have not been identified previously. In each genome, we discovered many of these new genes, which provide avenues for future discovery of new traits with the potential to manage plant diseases in agriculture or natural ecosystems.

## Introduction

*Pseudomonas* is a large genus within the γ subclass of Proteobacteria known for its ubiquity in the environment, utilization of a striking variety of organic compounds as energy sources [1], [2], and production of an array of secondary metabolites [3]–[5]. Some species include well-known pathogens such as *P. syringae*, which comprises many pathovars that are important plant pathogens, and *P. aeruginosa*, an opportunistic human pathogen. Others are not associated with disease and are prevalent in natural habitats, including soil, water, and plant surfaces. Certain strains live in a commensal relationship with plants, protecting them from infection by pathogens that would otherwise cause disease [6]–[8]. As such, *Pseudomonas* spp. function as key components of ecological processes that suppress plant diseases in agricultural and natural environments [9]–[11], and several strains are used commercially to manage plant diseases in agriculture [12].

The genus *Pseudomonas* currently comprises more than 100 named species that have been divided into lineages, groups and subgroups based on multilocus sequence analysis [13]–[15]. Many of the plant commensal strains fall into the *Pseudomonas fluorescens* group, which currently includes more than fifty named species [13]. Given this diversity, it is not surprising that individual plant-associated strains within the *P. fluorescens* group differ in many respects, including their capacity to suppress plant disease. For example, effective antagonists are typically identified only after screening large collections of isolates for plant disease suppression, indicating that only a subset of strains within the *P. fluorescens* group provide biological control. Successful biological control strains have certain characteristics in common: the capacity to colonize plant surfaces, specifically the infection court of target pathogens; and the production of antibiotics toxic to target pathogens or the induction of systemic resistance responses in the plant [8], [16]. Antibiotics, which function as major determinants of biological control, fall into diverse classes, including the phenazines [17], [18], polyketides [3], [19], cyclic lipopeptide biosurfactants [20], and many others. Some strains of *Pseudomonas* spp. also produce phytohormones [21]–[23] or metabolites that alter plant hormone levels [24], [25], directly influencing the growth and development of their plant associates [26]. Other strains induce resistance responses in plants against disease [27], [28]. Plant-commensal strains of *Pseudomonas* spp. are intricately enmeshed in plant and soil biology through all of these diverse activities, and their functions as biological control agents have distinguished them as microorganisms with significant effects on agricultural productivity.

Given the spectrum of ecological, metabolic, and biochemical characteristics of this genus, it is not surprising that diversity among *Pseudomonas* spp. extends to the genomic sequence level. The complete genomes of many species have now been sequenced [29], [30], and only 25% to 35% of the genome of each strain is composed of core genes shared by all members of the genus. Comparisons among the genomes of four strains within the *P. fluorescens* group (*Pseudomonas protegens* Pf-5 (previously called *P. fluorescens* Pf-5 [31]) and *P. fluorescens* strains SBW25, Pf0-1 and WH6 [32]–[34]) highlight the tremendous diversity of these bacteria. Of the 5741–6009 predicted protein-coding genes (referred to herein as the predicted proteome) identified in each genome, only 3115 are present in all four, composing a core genome representing only 52% to 54% of each strain. Furthermore, nearly a third (1488 to 1833 genes) of the predicted proteome for each strain is unique to that strain, again highlighting the heterogeneity of this group of bacteria.

The genomes of *Pseudomonas* spp., like those of many other bacteria, display a highly mosaic structure, being composed of relatively stable core regions interspersed with regions that vary among the strains [29], [32]–[34]. Regions that are unique to a specific strain are thought to shape that strain's distinctive characteristics, including its interactions with plant pathogens that are targets of biological control. Many of the unique genomic regions bear features of horizontally-acquired DNA (i.e., atypical trinucleotide content, lack of repetitive extragenic palindromic (REP) elements, or the presence of transposons, prophages, or genomic islands). Therefore, these features may be exploited as markers of genomic regions that define the distinctive attributes of an individual strain. For example, novel natural products including the cyclic lipopeptide orfamide A [35] and derivatives of rhizoxin [36], [37], and traits, such as LlpA bacteriocins [38] and the FitD insect toxin [39], have been discovered through genomics-guided approaches focused on strain-specific regions of the genome of *P. protegens* Pf-5. The combined repertoire of the core and variable regions of a genome reflects the ecological history of the strain and the various environments or selective pressures that it has encountered over evolutionary time.

To date, the sequenced strains represent only a fraction of the diversity within the *P. fluorescens* group, and much of the group's metabolic, ecological, and genetic diversity remains unexplored. Here, we provide a comparative analysis of strains within the group, and new genomic sequences for seven plant-associated strains. The seven newly-sequenced strains originate from habitats including soil, root and leaf surfaces from two continents, and exhibit biological control activities against bacterial, fungal and oomycete pathogens through varied mechanisms including antibiotic production, induced systemic resistance, and competitive exclusion (Table 1). Several of the strains were obtained from disease-suppressive soils that exhibit natural processes of biological control due to the presence of indigenous microflora antagonistic to soilborne plant pathogenic fungi or nematodes. Our results confirm the strain-to-strain variation observed previously in the *P. fluorescens* group, with several hundred genes unique to each of the new genomes. Within each genome, we discovered genes for traits that can be explored in the future for their roles in biological control and other heterotrophic interactions. To explore the evolutionary origin of these genes, we mapped their genomic distributions along with the sites of REP elements and mobile genetic elements (MGEs) to determine if the traits fell into the more ancestral or recently-acquired regions of the genomes. Finally, we complemented our genomic analysis with phenotypic screens to link the gene inventories to key phenotypes exhibited by plant-associated strains in the *P. fluorescens* group.

**Table 1:** Strains of the *Pseudomonas fluorescens* group.

Strain^a^	Source	Target disease(s) for biological control	Genome sequence
***P. chlororaphis*** ** subsp. ** ***aureofacien*** **s:**			
30-84	Wheat rhizosphere, Washington, USA	Take-all of wheat [18], [164]	This study
O6	Soil, Utah, USA [165]	Wildfire of tobacco [27], target spot of cucumber [166]	This study
***P. protegens:***			
Pf-5	Soil, Texas, USA	Seedling emergence [167], [168]	[33]
***P. brassicacearum*** **:**			
Q8r1-96	Wheat rhizosphere, Washington, USA	Take-all of wheat [169]	This study
***P. fluorescens*** **:**			
Pf0-1	Soil, Massachusetts, USA		[32]
Q2-87	Wheat rhizosphere, Washington, USA	Take-all of wheat [170]	This study
SBW25	Sugar beet phyllosphere, Oxfordshire, UK	Seedling emergence	[32]
A506	Pear phyllosphere, California, USA	Fire blight of pear and apple, frost injury, fruit russeting [12], [171]	This study
SS101	Wheat rhizosphere, The Netherlands	Diseases caused by *Pythium* spp. and *Phytophthora* spp. [172], [173], [174], [175]	This study
***Pseudomonas*** ** sp.:**			
BG33R	Peach rhizosphere, South Carolina, USA	The plant-parasitic nematode *Mesocriconema xenoplax* [176]	This study

a In previous publications, strain Pf-5 has been designated as *P. fluorescens* Pf-5, strain Q8r1-96 as *P. fluorescens* Q8r1-96, and strain BG33R as either *P. synxantha* or *Pseudomonas* sp. BG33R. Strains Q8r1-96 and Q2-87 were isolated in 1996 and 1987, respectively, from roots of wheat grown in the same field.

## Results/Discussion

### Genomic features

A summary of the features of each of the seven newly-sequenced genomes of biocontrol strains of *Pseudomonas* spp. is provided in Table 2. The characteristics (size, GC content, predicted number of coding sequences, and number of rRNA operons) are within the range of previously-sequenced genomes of *Pseudomonas* spp. [29]. Nevertheless, the seven genomes vary in size by approximately one megabase (ranging from 6.02–6.99 Mb) with the number of CDSs ranging from 5333–6224, indicating substantial strain-to-strain variation. The genomes of *P. chlororaphis* strains 30-84 and O6 and *P. protegens* Pf-5 are larger and have a higher GC content than those of the other strains. Only strain A506 has a plasmid, which will be described in detail in a separate publication.

**Table 2:** Genomic features.

Feature	Strains within the *Pseudomonas fluorescens* group									
	30-84	O6	Pf-5	Pf0-1	Q2-87	Q8r1-96	SBW25	A506	SS101	BG33R
Chromosome size (megabase pairs)	6.67	6.99	7.07	6.43	6.36	6.60	6.72	5.96	6.17	6.29
Plasmid size (kilobase pairs)								57.0		
G+C (%)	62.9	62.8	63.3	60.6	60.7	61	60.5	60	60	59.6
Protein-coding sequences (CDSs)	5849	6224	6108	5722	5597	5717	5921	5267	5374	5511
#CDSs on plasmid								66		
# pseudogenes	6	4	62	NA	8	8	NA	56	6	13
# conserved hypotheticals	882	979	884	NA	850	856	NA	812	833	855
# hypotheticals	108	143	303	NA	115	153	NA	168	155	183
Average CDS length (nt)	996	975	1016	1008	996	1008	999	991	1010	1013
Coding (%)	87.6	87.8	88.7	89.0	87.8	87.4	88.0	88.6	88.0	88.9
rRNA operons	6	ND	5	6	5	5	5	6	6	6
tRNA genes	74	ND	71	73	68	65	66	69	68	68
# scaffolds	9	9	1	1	2	5	1	1	1	2
# contigs	13	30	1	1	2	5	1	1	1	2

ND, not determined. Due to the large number of contigs for the O6 genome, the number of tRNAs and rRNA operons could not be determined accurately.

NA, data not available.

### Phylogenetic analysis

**Figure 1:**
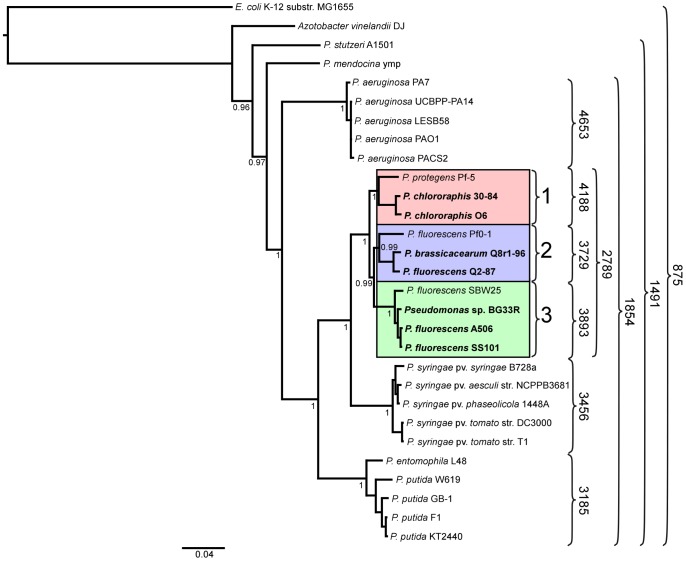
Phylogenetic tree depicting the relationships of sequenced strains of *Pseudomonas* spp. The tree is based on concatenated alignments of ten core housekeeping genes: *acsA*, *aroE*, *dnaE*, *guaA*, *gyrB*, *mutL*, *ppsA*, *pyrC*, *recA*, and *rpoB*, and was generated using the MrBayes package [152]. The interior node values of the tree are clade credibility values, which represent the likelihood of the clade existing, based on the posterior probability values produced by MrBayes. Strains in the *P. fluorescens* group fall within a single clade comprised of three sub-clades, which are numbered 1 to 3 and highlighted pink, blue and green, respectively. Strains sequenced in this study are in bold font. Numbers on the right of the figure represent the size of the core genome of the strains included within the curved brackets.

We inferred a phylogenetic tree using a Bayesian approach for representative strains of *Pseudomonas* spp. having fully sequenced genomes based on multilocus sequence analysis (MLSA) [40] (Figure 1). Along with three of the previously-sequenced strains within the *P. fluorescens* group (Pf-5, Pf0-1, and SBW25), the seven strains of this study fall into a single large clade composed of three sub-clades. The two strains of *P. chlororaphis* fall into Sub-clade 1, with strain Pf-5 more distantly associated with the group. Sub-clade 2 is composed of *P. fluorescens* Q2-87 and *P. fluorescens* Q8r1-96 (revealed as *Pseudomonas brassicacearum* Q8r1-96 in this study) and the previously-sequenced strain *P. fluorescens* Pf0-1, which is not as closely related to strains Q2-87 and Q8r1-96 as those two strains are related to one another. All of the strains in Sub-clades 1 and 2 were isolated from plant roots or soil in the USA (Table 1). In Sub-clade 3, strain A506, which was isolated from a leaf surface in California, USA, and strain SS101, isolated from wheat roots in The Netherlands, are most closely related. Sub-clade 3 also includes the previously-sequenced strain SBW25, isolated from a leaf of sugar beet in England, and *Pseudomonas* sp. BG33R (also called *P. synxantha* BG33R), isolated from roots of a peach tree in South Carolina, USA (Table 1). These results are reasonably consistent with a Bayesian phylogeny based on 16S rRNA (Figure S1) and very consistent with a maximum likelihood phylogeny constructed by concatenating 726 protein sequences present in the fully-sequenced strains of *Pseudomonas* spp. (Figure S2). These phylogenies also are congruent with those from a recent report in which a large number of strains representing many species of *Pseudomonas* were evaluated by MLSA [13]. In the MLSA study, strains of *P. fluorescens* and *P. chlororaphis* also were found to be in a distinct clade clearly distinguished from other *Pseudomonas* spp. Our MLSA analysis also is consistent with a recent report that assigned strain Pf-5 to the new species *P. protegens*, which is related to *P. chlororaphis* but also exhibits distinct properties [31].

### Core and pan-genome analysis

**Figure 2:**
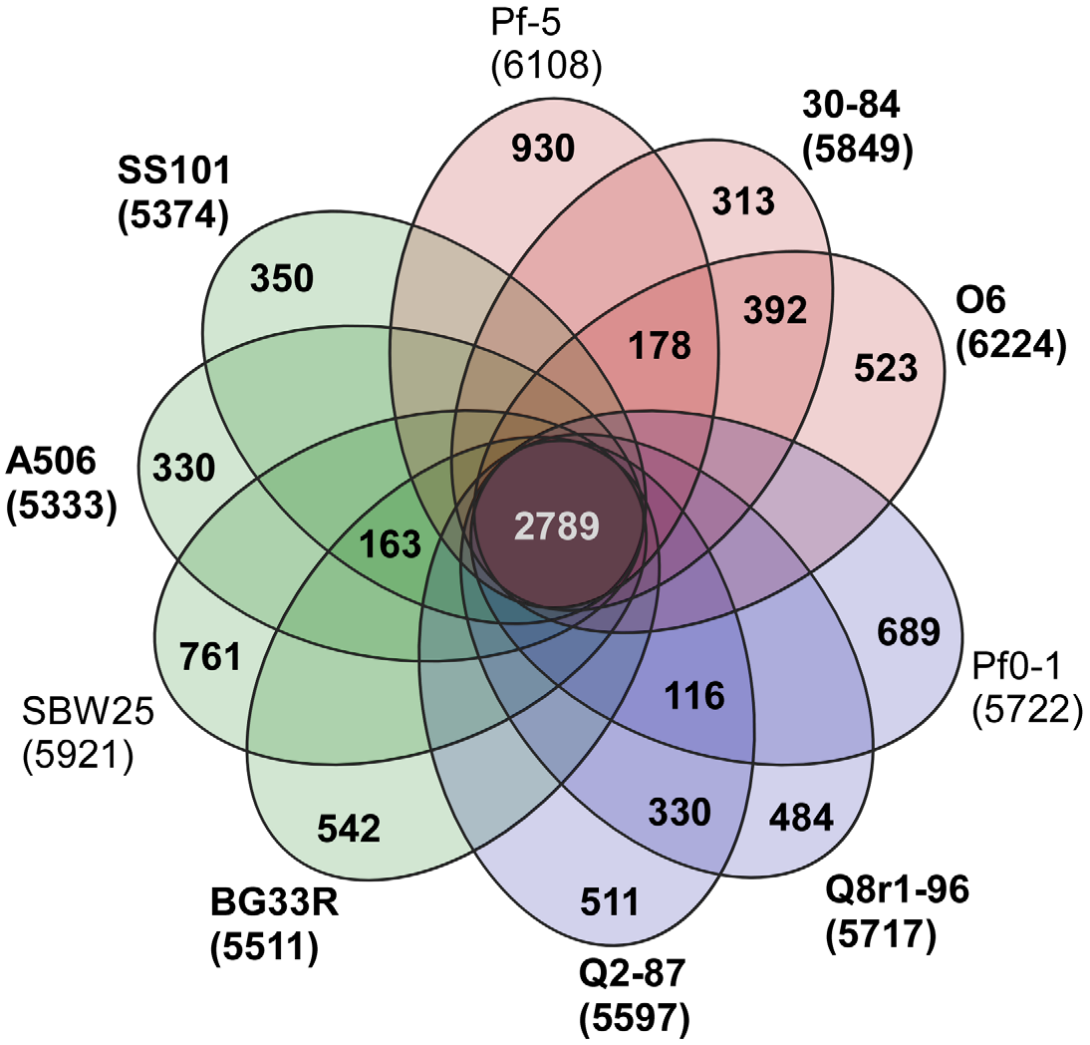
Genomic diversity of strains in the *P. fluorescens* group. Each strain is represented by an oval that is colored according to sub-clade (as in Figure 1). The number of orthologous coding sequences (CDSs) shared by all strains (i.e., the core genome) is in the center. Overlapping regions show the number of CDSs conserved only within the specified genomes. Numbers in non-overlapping portions of each oval show the number of CDSs unique to each strain. The total number of protein coding genes within each genome is listed below the strain name. Strains sequenced in this study are in bold font.

A core genome containing 2789 predicted protein coding genes was identified for the *P. fluorescens* group from a ten-way best-match BLASTp search (Figure 1, Figure 2). This core genome represents only 45% to 52% of the predicted proteome of each strain, further illustrating a large degree of genomic diversity in this group of bacteria. The size of the core genome in the *P. fluorescens* group is considerably smaller than that of *P. aeruginosa*, which we have estimated to be 4653 putative protein-coding genes based on comparative BLASTp searches among five sequenced isolates (Figure 1), but is closer to the core genome sizes we estimated for strains of *P. syringae* and *P. putida/entomophila*, 3456 and 3185 CDSs, respectively (Figure 1). This estimate is also somewhat smaller than earlier estimates based upon the previously-sequenced genomes of strains within the *P. fluorescens* group [29], [32], [34], [41], which is to be expected as the number of strains available for comparison increases. Genes conserved among all of the genomes encode proteins contributing mainly to fundamental housekeeping functions, such as protein and nucleic acid synthesis, whereas genes encoding hypothetical proteins and those associated with mobile elements are underrepresented in the core genome (Table S1).

Of the 2789 core genes, only 20 are specific to the *P. fluorescens* group (Table S2); the other 2769 genes have orthologs in at least one other sequenced genome of *Pseudomonas* spp. Annotated functions of the 20 core genes include biofilm formation, hypothetical or conserved hypothetical proteins and regulation (Table S2). We attribute the remarkably small number of core genes distinguishing this group from other *Pseudomonas* spp. to the diversity of strains within the *P. fluorescens* group and the highly plastic nature of their genomes. This diversity also is reflected in the large size of the pan-genome, which, at 13,872 putative protein-coding genes, is substantially larger than that estimated here for *P. aeruginosa* (7,824 CDSs). The pan-genome of the *P. fluorescens* group also exceeds that estimated here for *P. syringae* (9,386 CDSs) based on the five strains considered in our analysis (Figure 1), but is only slightly larger than the pan-genome of 19 strains of *P. syringae* (12,829 CDSs) estimated by Baltrus et al. [30]. Of the 13,872 CDSs composing the pan-genome of the *P. fluorescens* group, 5798 have no orthologs in other genomes of *Pseudomonas* spp., which probably is due to a high level of differentiation of genes in the group and a high frequency of horizontal gene acquisition from other taxa. It is also likely that the large gene inventory in *Pseudomonas* spp. is not yet reflected in the relatively small number of genomes sequenced to date.

**Figure 3:**
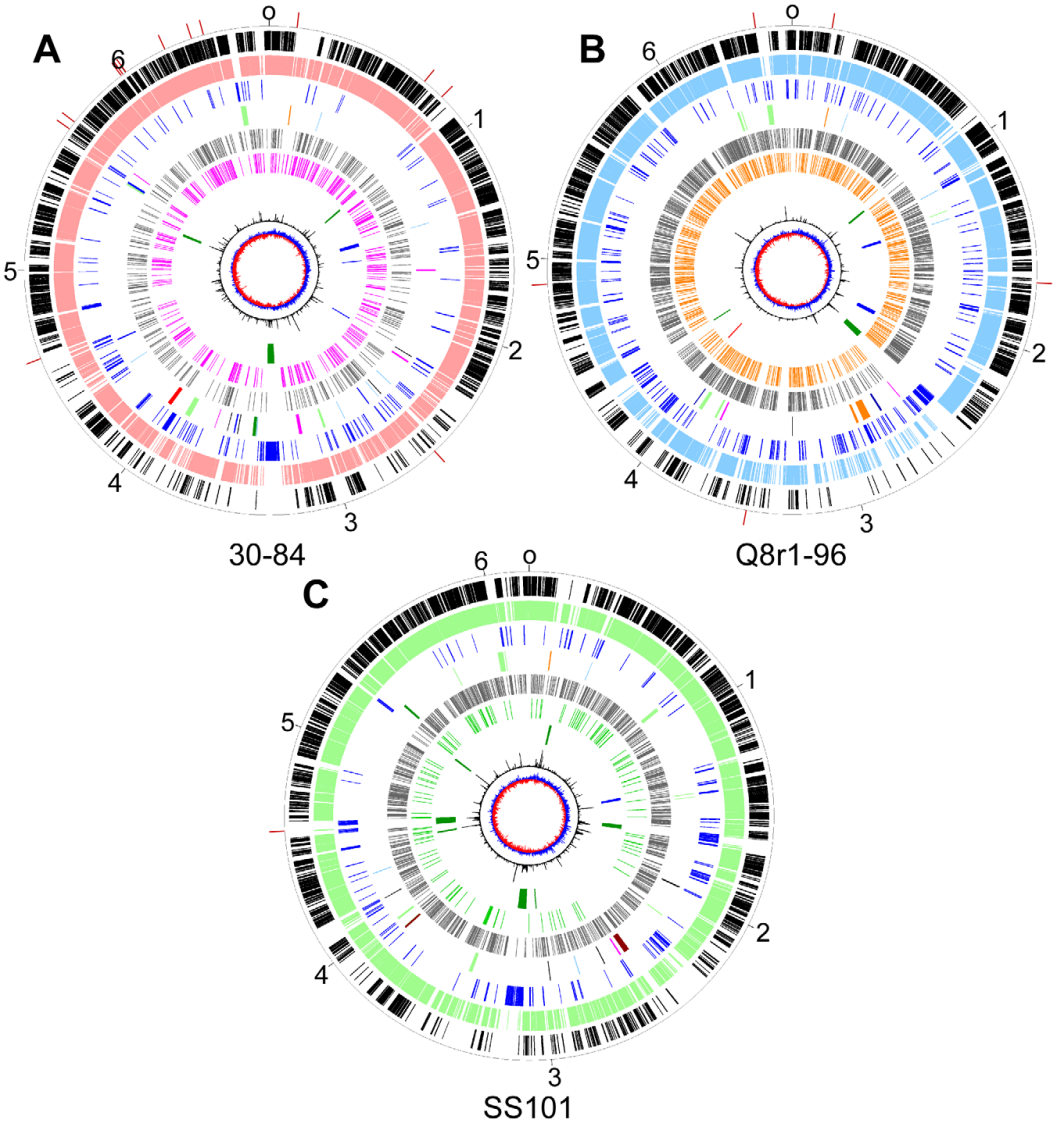
Circular genome diagrams of representative strains from each of three sub-clades in the *P. fluorescens* group. *P. chlororaphis* 30-84, Sub-clade 1 (A); *P. brassicacearum* Q8r1-96, Sub-clade 2 (B), and; *P. fluorescens* SS101, Sub-clade 3 (C). The outer scales designate the coordinates (in Mb) and the red marks indicate the boundaries of scaffolds. The first (outer-most) circles show the core genes shared across *P. aeruginosa*, *P. syringae*, *P. putida* and the *P. fluorescens* group (black). The second circles show the core genes conserved within each respective sub-clade (Sub-clade 1, pink; Sub-clade 2, blue, and; Sub-clade 3, green). The third circles show genes unique to each strain (blue). The fourth circles show the locations of genes or gene clusters coding for the production of antibiotics (blue), cyclic lipopeptides (brown), siderophores (dark green), orphan clusters (orange), bacteriocins (light blue), plant communication (magenta), exoenzymes (black), secretion systems (light green) or insect toxins (red). The fifth and sixth circles show the positions of repetitive extragenic palindromic elements; REPa (grey), REPb (magenta), REPc (green) and REPd (orange, in Q8r1-96 using the REP HMM trained on SBW25 sequences). The seventh circles show the locations of putative mobile genetic elements; genomic islands (dark green), prophage (blue) and transposons (red). The eighth circles show the trinucleotide content (black lines) and the ninth circles show the GC-skew.

Pairwise comparisons of predicted proteomes supported the phylogenetic relationships among strains illustrated in the MLSA analysis. For example, strains within a sub-clade (Figure 1) share 69–90% of their predicted proteomes, whereas strains in different sub-clades share only 64–73% of their proteomes (Figure 1, 3). Correspondingly, the core genomes for each sub-clade are substantially larger than the core genome for the group as a whole, ranging from 3729 to 4188 CDSs among the three sub-clades (Figure 1). Pair-wise BLASTp analyses also offered some support for the relatively distant relationship of strain Pf-5 with Sub-clade 1 and of strain Pf0-1 with Sub-clade 2. Indeed, using the level of shared gene content as an indicator of relatedness, strain Pf0-1 is more closely related to strains in Sub-clade 1 than to Q8r1-96 or Q2-87. Of note, the size of core genomes of Sub-clades 1 and 2 increased by 1045 or 912 CDSs, respectively, when only the two more closely-related strains in each of these sub-clades were used for comparison (Figure 1, Table S4).

Whole genome alignments of the strains in the *P. fluorescens* group were conducted to gauge the level of synteny. There is a relatively high level of synteny around the origin of replication for strains within a single sub-clade (Figure S3, Figure S4, Figure S5), but very little synteny is evident between genomes of strains in different sub-clades. As has been described for a number of bacterial genomes, including *P. fluorescens* [32], [33], the majority of unique genes and genome rearrangements have occurred around the terminus of replication. This is evident from the distribution of core genes, which are concentrated near the origin of replication of each genome (Figure 3). Nonetheless, the current assemblies suggest that inversion events may have taken place near the origin of replication in strain Q2-87 (Figure S4).

The combination of the phylogenetic analysis and the comparative BLASTp dataset provided an opportunity to identify genes that differentiate each sub-clade. The three genomes in Sub-clade 1 share 73 genes that are not present in any other sequenced *Pseudomonas* genome (Table S5). These include genes encoding biosynthesis of the antimicrobial pyrrolnitrin and the insect toxin FitD. Within this clade, the two *P. chlororaphis* strains share 255 genes that are not found in other sequenced strains of *Pseudomonas* spp. (Table S6). These genes, which may be characteristic of the species, include a cytochrome *c* oxidase system, bacteriocins, type I secretion system components and several secondary metabolite biosynthesis gene clusters. The three genomes in Sub-clade 2 share 38 genes that are not present in any other sequenced *Pseudomonas* genome (Table S7). These genes include a lipase and putative type VI secretion system effectors. Strains Q2-87 and Q8r1-96 share 195 genes that are not found in other *Pseudomonas* genomes, including components of type I and type III secretion systems (Table S8). Strains in Sub-clade 3 share 87 genes that are not found in other strains of *Pseudomonas* spp., including genes for pili biosynthesis, components of type III secretion systems, and ribose utilization (Table S9).

Each of the ten genomes of the *P. fluorescens* group includes ca. 300 to 900 genes (6 to 15% of the predicted proteome) that are unique to that strain (Figure 2). This estimate of strain-specific genes is smaller than earlier estimates (ca. 19–29% of the predicted proteome) [32], [34], [41], which is not surprising because the number of unique genes is expected to fall as the number of strains available for comparison increases. Both the large number of strain-specific genes and the large size of the pan-genome indicate a high level of genomic diversity consistent with the observed biological diversity of the *P. fluorescens* group, including the distinctive biocontrol properties of the strains.

### Defining the core and lineage-specific regions of the genomes

We used four criteria to distinguish regions of the *Pseudomonas* genomes that are more ancestral from those that may have been more recently acquired: i) distribution of the genes unique to each strain as well as the core genes shared among all strains, ii) atypical trinucleotide composition, iii) presence of putative MGEs, and iv) distribution of repetitive extragenic palindromic (REP) elements (Figure 3).

#### REP elements

REP elements are short nucleotide sequences, typically 20–60 nt long, that are abundant in the intergenic regions of many *Pseudomonas* spp. [32], [33], [42]–[45]. Functions of REP elements remain in question but they may provide sites for DNA gyrase or DNA polymerase I binding, or for recombination [42]–[44]. REP elements appear to accumulate within the non-coding regions of genomes over time; they are rarely associated with regions of atypical trinucleotide content but display a similarly global distribution to core genes [33]. Therefore, REP elements have been used as markers of older, more stable regions of the genome [33]. Nonetheless, selective pressures are likely to prevent their incorporation within important housekeeping regions where they may disrupt the function of essential cellular processes [32]. For example, there are typically no REP sequences located near the chromosomal replication origin.

The genomes of the *P. fluorescens* group were examined for the presence of REP elements using a combination of basic repeat searches and Hidden Markov Model (HMM) searches. At least one type of REP element occurring at least 250 times was observed within the non-coding regions of each genome, except that of *P. fluorescens* Pf0-1 (Table S10, Table S11). In several genomes, two distinct REP elements were identified. To examine the level of conservation of REP elements within the group, HMMs trained on REP sequences from each strain were used to search the genomic sequences of all other strains. This analysis revealed that one primary REP sequence, referred to here as REPa, was conserved, but not identical, among the strains; HMMs trained on REPa sequences from one strain typically identified a large number of these sequences within the genomes of other strains (Table S11). Interestingly, the HMMs trained on REPa sequences from strains in the *P. fluorescens* group also detected a large number of copies of this element in genomes of *P. putida* and a small number of copies in genomes of *P. syringae* (Table S11).

In addition to the primary REPa elements, secondary REP elements were identified in a number of the genome sequences. The first of these, REPb, was identified in both *P. chlororaphis* strains and at lower abundance in *P. protegens* Pf-5 (Table S10, Table S11). Given that these strains are phylogenetically related within Sub-clade 1 (Figure 1), this sequence may be sub-clade specific. In contrast, two other secondary REP sequences, REPc and REPd, display unique and scattered distributions among strains in Sub-clades 2 and 3. Another secondary REP element, REPe, was identified only within the genome of *P. fluorescens* SBW25 (Table S10, Table S11).

REP sequences frequently are organized into pairs or clusters displaying inverted orientations [46]. This organization may be related to their mechanism of dispersal. Recent work has provided evidence for the involvement of a family of IS*200*/IS*605*-like REP-associated tyrosine transposases (RAYTs) in REP sequence maintenance and within-genome propagation, where REP pairs (REP doublets forming hairpins; REPINs) are likely to be the minimal mobilizable unit [42], [44]. As recently described in *P. fluorescens* SBW25 [44], the majority of REP sequences identified within the newly-sequenced genomes were found as oppositely oriented pairs separated by a uniform distance, typically 60–70 bp (Figure S6).

**Figure 4:**
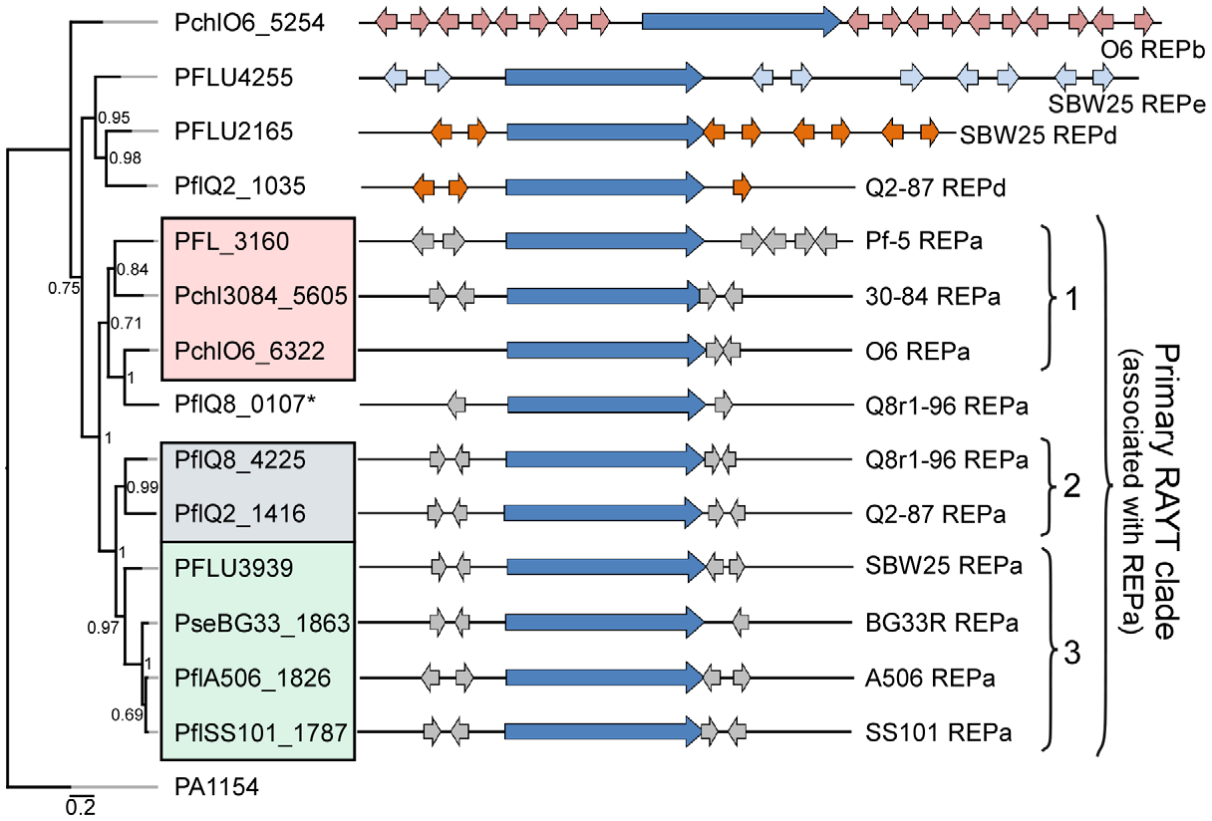
Repeated extragenic palindromic (REP) elements and REP–associated tyrosine transposases (RAYTs) of the *P. fluorescens* group. The left panel shows a phylogenetic tree, generated using the MrBayes package [152], depicting the relationships between RAYT proteins identified within each strain of the *P. fluorescens* group. The interior node values of the tree are clade credibility values, which represent the likelihood of the clade existing, based on the posterior probability values produced by MrBayes. The locus tags for primary RAYT proteins are shaded according to sub-clade using the color scheme of Figure 1; locus tags for secondary RAYT proteins are not shaded. The second primary RAYT in Q8r1-96 is within PflQ8_0107, in a separate reading frame. The right panel shows schematic representations of the RAYT genes (dark blue arrows) and the locations of associated flanking REP elements (REPa sequences in grey; REPd in orange; and REPe in light blue). The *P. aeruginosa* RAYT protein encoded by PA1154 is used as an outgroup, since this RAYT protein was shown previously to fall within a clade separate from the *P. fluorescens* RAYT proteins [44].

We identified at least one RAYT gene in each genome sequence except that of *P. fluorescens* Pf0-1. Phylogenetic analysis of the RAYT protein sequences revealed a major clade containing an orthologous RAYT protein in the other nine genomes (Figure 4). In those nine genomes, the RAYT-encoding gene in this major clade is flanked by copies of the REPa element, suggesting that these RAYT orthologs could be involved in the maintenance and propagation of REPa sequences. Interestingly, the sub-clade structure of the major RAYT clade closely resembles that seen in the MLSA tree of *Pseudomonas* strains (Figure 1, Figure 4), suggesting that these RAYT genes may have been a stable part of the genomes since their divergence. Additional support for this hypothesis comes from the observation that related REPa-associated RAYT genes from Sub-clades 2 and 3 are located within regions of local synteny. Notably, the genome of strain Q8r1-96 harbors two RAYT genes flanked by copies of the REPa sequence (Figure 4). One of these RAYTs (PflQ8_4225) is similar to that encoded by Q2-87 and, as stated above, is encoded in a region of local synteny. The second RAYT in the Q8r1-96 genome is similar to the Sub-clade 1 RAYT proteins and, therefore, may have been acquired laterally from a Sub-clade 1-like strain (Figure 4). Previous studies described a relationship between the number of REP elements within a genome and the presence of a cognate RAYT gene [42]. This trend also is apparent in the strains of this study, most of which carry between 500 and 1500 copies of the REPa sequence element and a single cognate RAYT protein. A larger number of REPa sequences were found in the genome of Q8r1-96, which has two putative cognate RAYT genes. In contrast, very few REP elements are present in the genome of *P. fluorescens* Pf0-1, which has no RAYT gene. Additionally, RAYT genes associated with REPb, REPd and REPe sequences, which are abundant within their respective genomes, were identified in a number of strains (Figure 4). No RAYT genes were found to be associated with REPc sequences, which are at relatively low abundance in the genomes of several strains (Table S11). Interestingly, the Pf-5 genome has 999 copies of REPa but has a mutation in the RAYT gene, which introduced a stop codon and is likely to inactivate its function. It is possible that REPa sequences were dispersed in the Pf-5 genome prior to the mutation in the RAYT, which may have occurred relatively recently. Overall, however, these observations support the role of RAYT proteins in the propagation and maintenance of their cognate REP sequences.

The REP elements are not uniformly distributed in the genomes of the *P. fluorescens* group, and regions lacking REP sequences are striking in the genomes evaluated here (Figure 3), as described previously for Pf-5 [33] and SBW25 [32]. These regions, termed REP deserts, vary in number among the genomes. For example, using an arbitrary lower limit of 25 kb to define a REP desert, the four genomes in Sub-clade 3 have 31 to 66 REPa deserts, totaling 1.2 to 3.2 Mb in size (20% to 47% of the genome). Defining deserts for the secondary REPs, which are present in fewer copies than REPa in all genomes, is difficult; but in many cases, regions lacking secondary REP elements encompass REPa deserts (Figure 3). The REPa deserts commonly correspond to atypical regions of the genome, defined by atypical nucleotide composition, and some of the REPa deserts contain mobile genetic elements (Figure 3).

#### Mobile genetic elements (MGEs)

MGEs were defined in this study as genome segments encoding putative functions linked to the intra- and extracellular movement of DNA in bacteria and/or bearing traces of recent horizontal gene transfer events.

Each *Pseudomonas* genome in this study contains a unique set of transposons (Table S12). The number of transposon copies per genome ranges from six (SS101) to twenty (O6) with about half of the copies likely to be rendered inactive by frameshift mutations and/or deletions. Members of the IS3, IS4, IS5 and IS66 families are most common. Among notable transposon-related features is a 5.2 kb composite transposon from Q8r1-96 with a putative pathway for catabolism of the broadleaf herbicide bromoxynil. The transposon, which is the only composite transposon in these genomes, is comprised of two IS elements of the IS5 family flanking a group of genes that encode a LysR-like transcriptional regulator, a transporter of the sodium solute superfamily, and a bromoxynil-specific nitrilase (Transposon 1). Another interesting transposon-related feature is found in strain 30-84, where two putative insecticidal toxin genes are found adjacent to genes encoding site-specific integrases and a Tn402-like transposase (Island 2). The type of genes present, their overall arrangement, and lack of flanking inverted repeats suggest that this genomic region may represent an integron remnant.

**Figure 5:**
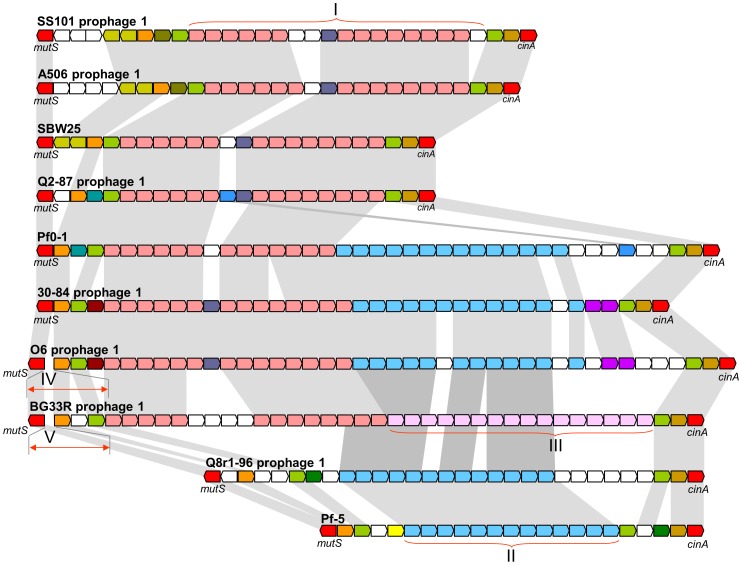
Comparative organization of prophages in the *mutS-cinA* region of ten genomes of the *P. fluorescens* group. Predicted genes and their orientation are shown by arrows. The conserved housekeeping genes *mutS* and *cinA* are colored red, whereas strain-specific genes are colored white. Homologous prophage genes are indicated by other colors and connected with grey shading. Roman numerals correspond to conserved blocks of bacteriophage genes shared among strains. The size of genes and intergenic regions are not to scale.

Genomes of all of the strains contain one to four prophages and/or prophage remnants, each ranging in size from 3.4 to 72.3 kb. Collectively, the seven newly-sequenced genomes have 18 prophages, most of which have a set of cargo genes that are distinct from those in prophages of other strains (Figure S7). Notable exceptions are the prophages integrated in the *mutS/cinA* region (Prophage 1 of each genome), each of which carries a subset of five distinct bacteriophage gene cassettes (Figure 5). These prophages display the mosaic structure that characterizes prophages in other *Pseudomonas* spp. [47]. The remaining prophages in the seven genomes carry a diverse array of cargo genes that encode putative bacteriocins, UV resistance proteins, adenine- and cytosine-specific DNA methyltransferases, and conserved hypothetical proteins (Table S13). In addition, a prophage remnant in strain SS101 contains two gene clusters encoding components of chaperone-usher machinery (Island 2). Each cluster encodes an usher, a chaperone, and two fimbrial subunits that may be involved in the production of cell surface-associated appendages similar to Cup fimbriae of *P. aeruginosa* [48]. In *P. aeruginosa*, these fimbriae are involved in bacterial surface attachment and biofilm formation [49], [50]. Homologous loci are present in *P. syringae* and *P. putida*, but their precise roles, as well as the role of the chaperone-usher machinery in SS101, remain to be discovered.

In addition to transposons and prophages, the genomes carry three to seven genomic islands ranging from 2.3 to 154.3 kb in size (Table S13). Collectively, the seven newly-sequenced genomes have 32 genomic islands. Among the cargo genes of these islands are those with predicted functions as components of restriction-modification systems (Island 1 in 30-84; Island 1 in Q8r1-96), assorted transporters (Island 1 in 30-84; Islands 3 and 6 in SS101; Islands 3 and 4 in BG33R), transcriptional regulators (Island 2 in O6; Island 4 in Q8r1-96; Island 2 and 4 in Q2-87; Islands 2, 4, and 6 in SS101; Island 7 in BG33R), two-component signal transduction systems (Island 3 in 30-84; Islands 1, 2, and 3 in O6; Island 6 in SS101), a methyl-accepting chemotaxis protein (Island 4 in SS101), a polyphosphate kinase (Island 3 in 30-84; Island 2 in O6), a TonB-dependent outer-membrane receptor (Island 1 in O6; Island 3 in BG33R), a putative β-lactamase (Island 4 in SS101), a UV irradiation resistance protein (Island 4 in SS101), and a diverse array of conserved hypothetical proteins (all strains). Other notable features include gene clusters for a mevalonate-independent pathway of isoprenoid production and a type VI secretion system (Island 3 of strain O6), a chaperone-usher fimbrial biogenesis pathway (Island 6 of strain SS101), and an indole-3-acetic acid uptake and catabolism pathway (Island 3 of BG33R). Finally, several genomic islands also contain transposons (Island 2 in 30-84; Island 3 in O6; Islands 1 and 2 in Q2-87; Island 6 in SS101) and genes of bacteriophage or plasmid origin (Island 2 in 30-84; Islands 1 and 2 in O6; Island 3 in A506; Islands 1, 2, and 3 in Q8r1-96; Islands 1 and 5 in Q2-87; Islands 4 and 6 in SS101; and Islands 1, 3, and 6 in BG33R) (Table S13).

Plasmid-like elements were identified in strains A506 and BG33R. Strain A506 carries a 57-kb cryptic plasmid, pA506, which has features in common with the pPT23A family of plasmids, members of which are widespread in *P. syringae* [51]. pA506 and the pPT23A plasmids share genes involved in replication, mating pair formation and conjugative transfer. pA506 also contains a type IV secretion gene cluster interrupted by the insertion of 11 genes that encode components of type IV conjugative pili similar to those of the pathogenicity island PAPI-1 from *P. aeruginosa* PA14 [52]. Other plasmid-borne genes have putative functions as integrases and components of a lesion-bypass DNA polymerase RulAB that may contribute to tolerance of UV-induced DNA damage in A506.

Strain BG33R harbors a 154-kb genomic island, Island 3, which belongs to a class of mosaic elements known as integrative conjugative elements (ICEs) [53]. ICEs resemble conjugative plasmids carrying bacteriophage-like integrase genes and are capable of site-specific integration into bacterial genomes. In the genome of BG33R, Island 3 is integrated into one of the five tRNA-Gly genes. Genes for site-specific integration, plasmid maintenance and conjugation span almost half of the island and are similar to their counterparts in the PFGI-1 ICE of *P. protegens* Pf-5 [54]. However, unlike PFGI-1, Island 3 lacks genes encoding conjugative pili and therefore appears to be anchored in the genome of BG33R. The presence of a fragment of *pilS* suggests that, at some point in time, Island 3 contained a functional conjugative pilus gene cluster that subsequently underwent deletion. Island 3 also contains genes encoding putative pathways for uptake and catabolism of IAA, quinolones, and haloaromatic compounds, a MexCD-like multi-drug resistance efflux pump and other transporters, a pertussis toxin subunit-like protein, several transposases, and regulatory and conserved hypothetical proteins.

CRISPRs (Clustered Regularly Interspaced Short Palindromic Repeats), loci responsible for prokaryotic immunity to phage infection, were not found in any of these strains. Putative CRISPRs identified using the CRISPRFinder program [55] are present within called genes, rather than in intergenic regions as expected, and are not contiguous to genes encoding typical CRISPR-associated proteins that are required for CRISPR functionality.

As expected for elements acquired horizontally, the MGEs map to regions of the genomes having atypical nucleotide composition and devoid of REP elements (Figure 3). The genes carried by MGEs contribute to the heterogeneity of strains in the *P. fluorescens* group, comprising 2% to 6% of each of the genomes, ranging from 131.8 kb (in Q2-87) to 379.2 kb (in BG33R). Nevertheless, they make up a small proportion of the genetic variation seen within the group, and many of the strain-specific regions of the genomes do not exhibit the distinct hallmarks of MGEs (i.e., transposons, integrases, prophages, or conjugative elements) described above.

### Phylogenetic distribution of traits involved in plant-microbe interactions

We surveyed the genomes of each strain for the presence of loci associated with biological control, including secondary metabolite biosynthesis and bacteriocin production. We also identified loci contributing to the interactions of *Pseudomonas* spp. with plant and animal host cells and the environment, such as secretion systems for export of exoenzymes, proteinaceous effectors, and toxins. The locations of these loci were mapped onto the genomes of each strain, along with the locations of unique and core genes, regions of atypical trinucleotide composition, MGEs, and REP elements, to provide insight into the evolution of traits contributing to the distinctive biology of each strain.

#### Secondary metabolite biosynthesis

**Figure 6:**
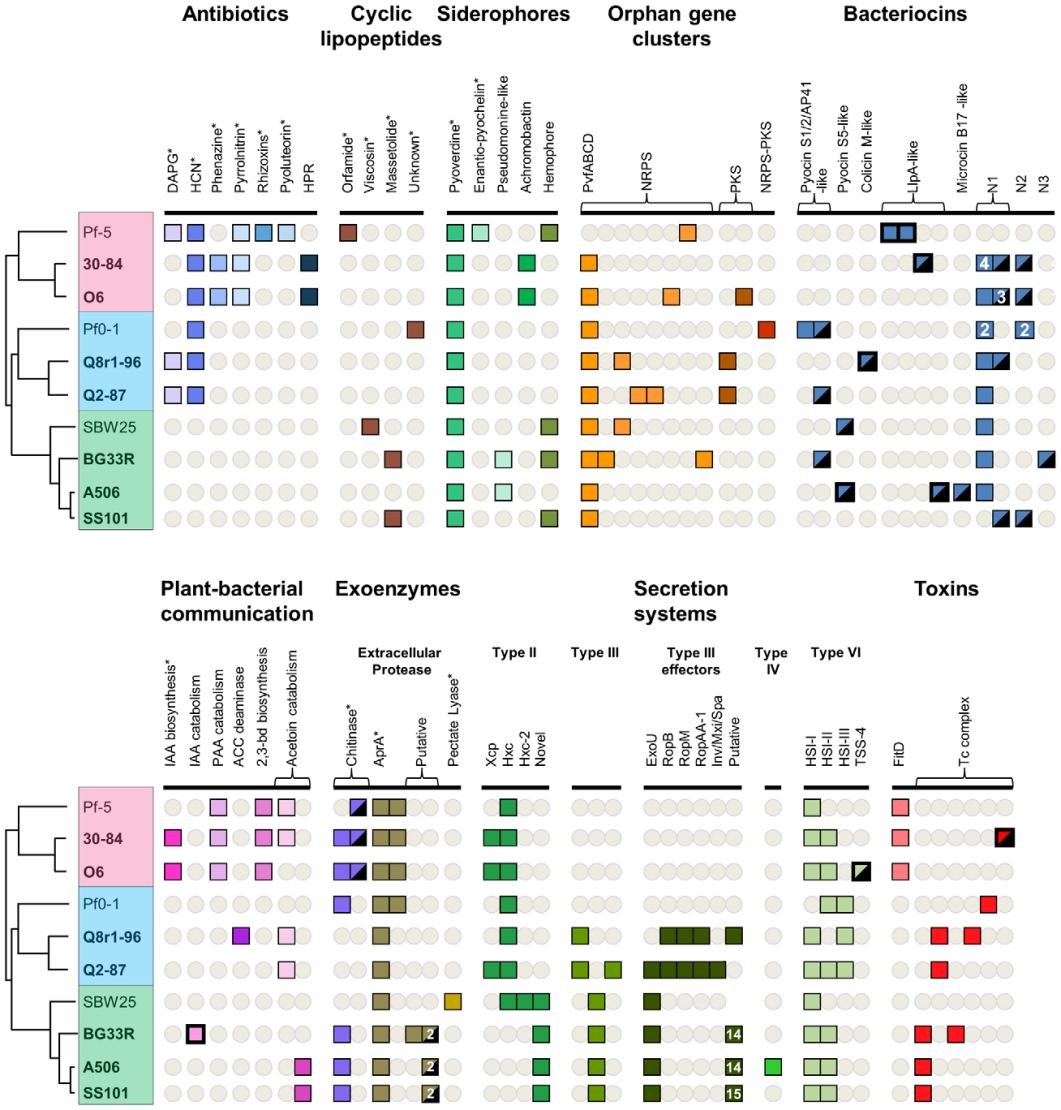
Selected biosynthetic/catabolic genes or gene clusters in the sequenced strains of the *P. fluorescens* group. Colored boxes represent the presence of a gene or gene cluster within a genome, while absence of a cluster is represented by a grey circle; numbers within a box represent the number of copies of a gene or cluster within a genome. Putative T3SS effectors were not examined for SBW25, therefore no box or circle is present in that column for SBW25. Genes within a mobile genetic element have the box outline bolded; genes within regions of atypical trinucleotide content have half of their boxes blackened. Plant-bacterial communication gene clusters are composed of: *iaaMH* (IAA biosynthesis); *iacR*, an ABC transporter, and *iacHABICDEFG* (IAA catabolism); *paaCYBDFGHIJKWLN* (PAA catabolism); *acdS* (ACC deaminase); *budC/ydjL+ilvBN* (2,3-butanediol biosynthesis); *acoRABC+acoX+bdh* (light pink, acetoin catabolism); *acoRABC+budC* (dark pink, acetoin catabolism). Abbreviations are as follows: 2,4-diacetylphloroglucinol (DAPG); hydrogen cyanide (HCN); derivatives of rhizoxin (Rhizoxins); 2-hexyl-5-propyl-alkylresorcinol (HPR); non-ribosomal peptide synthetase (NRPS); polyketide synthase (PKS); novel groups 1–3, respectively, of the carocin- and pyocin-like bacteriocins found in these strains (N1, N2, N3); indole-3-acetic acid (IAA); phenylacetic acid (PAA); aminocyclopropane-1-carboxylic acid (ACC); type VI secretion systems found within virulence loci HSI-I, HSI-II, and HSI-III, respectively, of *P. aeruginosa* (HSI-I, II, II); TSS-4 from *Burkholderia pseudomallei* (TSS-4). Asterisks indicate that the expected phenotype is known to be expressed or was detected in this study by the strains having the indicated genes or gene clusters.

Compounds toxic to phytopathogenic fungi, oomycetes, and bacteria are important contributors to biological control, and collectively, the strains evaluated herein are known to produce phenazines [17], [18], hydrogen cyanide, the chlorinated tryptophan derivative pyrrolnitrin, and the polyketides 2,4-diacetylphloroglucinol, rhizoxin and pyoluteorin [3]. Gene clusters for each of these compounds were identified in the genomic sequences of the producing strains (Figure 6). In addition to these known gene clusters, a locus similar to the characterized 2-hexyl-5-propyl-alkylresorcinol biosynthesis gene cluster of *Pseudomonas chlororaphis* subsp. *aurantiaca* BL915 [56] was identified in the two *P. chlororaphis* genomes. 2-hexyl-5-propyl-alkylresorcinol exhibits moderate antifungal and antibacterial activity and, if produced by *P. chlororaphis* O6 and 30-84, could contribute to their suppression of fungal and bacterial plant pathogens.

**Figure 7:**
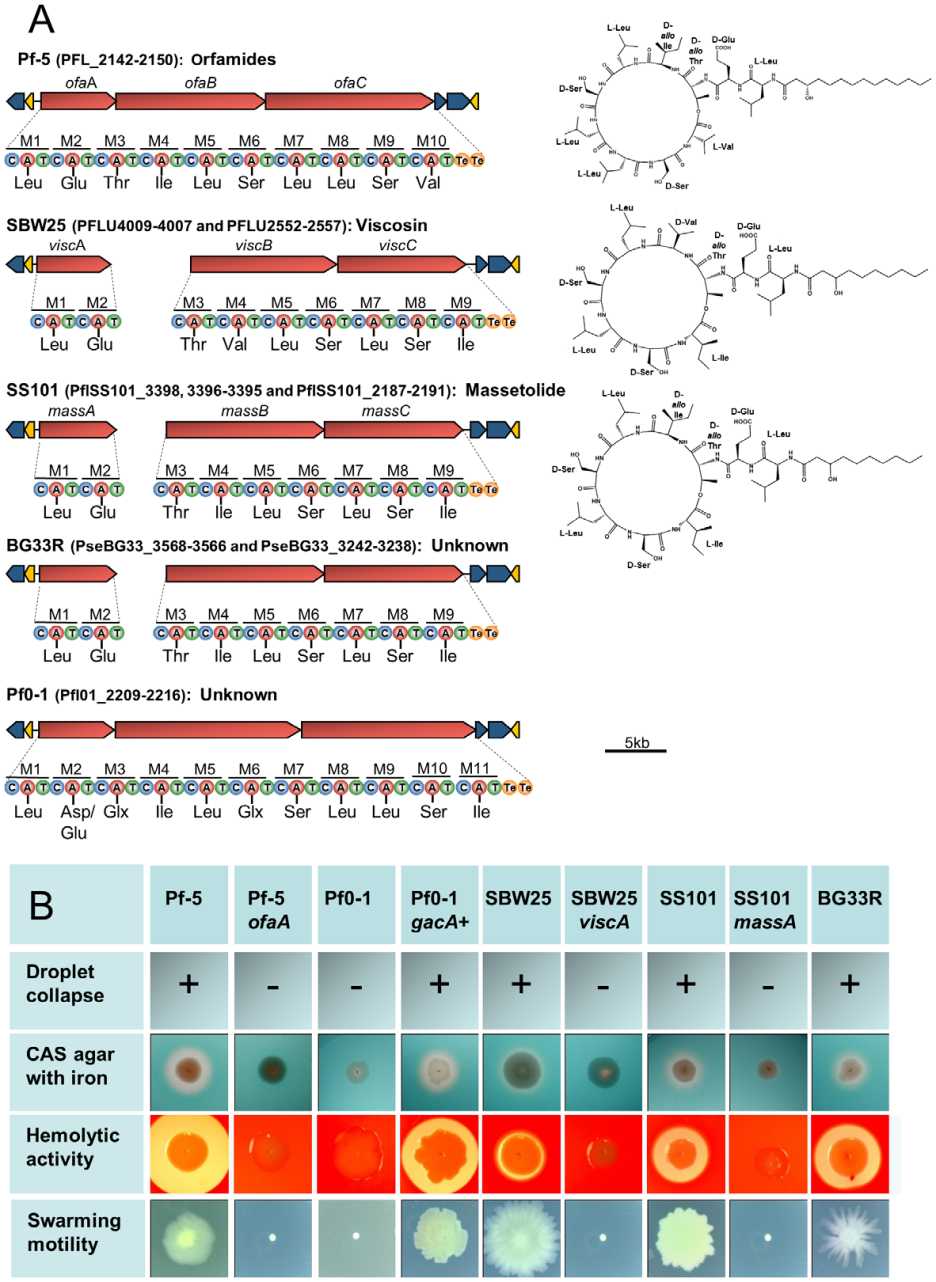
Biosynthetic gene clusters, predicted structures, and phenotypes associated with cyclic lipopeptide (CLP) production by strains in the *P. fluorescens* group. (A) Organization of the clusters and predicted amino acid composition of the CLP peptide chains in five genomes. NRPSs (red arrows) have nine to eleven modules (M1-M11) each containing a condensation (C), adenylation (A), and thiolation (T) domain, with two thioesterase domains (Te) at the terminus. Amino acids predicted to be incorporated into the CLP peptide are shown beneath each adenylation domain. Structures of orfamide A [35], viscosin [60], and massetolide [59] are shown to the right of the corresponding gene clusters. The organization of the biosynthetic clusters, which include genes encoding LysR regulators (yellow arrows) and efflux proteins (blue arrows), is similar among the genomes. (B) Phenotypes associated with CLP production. Strains Pf-5, SBW25, SS101 and BG33R, which have CLP biosynthetic clusters, exhibited surfactant activity, determined by a droplet collapse assay; produced zones on CAS agar containing 0.1 mM FeCl_3_; expressed hemolytic activity; and exhibited swarming motility. Mutants deficient in CLP biosynthesis (Pf-5 *ofaA*, SBW25 *viscA*, and SS101 *massA*) did not express these phenotypes. The four phenotypes also were expressed by a derivative of Pf0-1 containing the *gacA^+^* gene from Pf-5, but not by Pf0-1 or a derivative containing the *gacS^+^* gene from Pf-5 (data not shown).

Cyclic lipopeptides (CLPs), composed of a lipid tail linked to a cyclic oligopeptide, are a class of compounds produced by many strains of *Pseudomonas* spp. that exhibit surfactant, antimicrobial, anti-predation, and cytotoxic properties [20], [35], [57], [58]. The structural diversity of the CLPs is due to differences in the length and composition of the lipid moiety as well as in the type, number and configuration of the amino acids in the peptide chain. These compounds are synthesized via a non-ribosomal mechanism of peptide synthesis and genes encoding non-ribosomal peptide synthetases (NRPSs) are clustered with those having efflux and regulatory functions in the CLP biosynthetic loci of *Pseudomonas* spp. Genes coding for production of the CLP orfamide A are present in a single gene cluster in the Pf-5 genome [35], whereas orthologs for the CLPs massetolide A and viscosin are present in two distinct locations in the genomes of *P. fluorescens* SS101 and SBW25, respectively [59], [60] (Figure 7). We identified gene clusters for CLP biosynthesis in the genomes of BG33R and Pf0-1, and found that strain BG33R exhibited phenotypes (swarming motility, hemolytic activity, and surfactant activity) associated with CLP production. Although these phenotypes were not expressed by Pf0-1, they were exhibited by a derivative of Pf0-1 containing the *gacA* ^+^ gene from strain Pf-5 (Figure 7, Table S14) but not by a derivative of Pf0-1 containing the *gacS* ^+^ gene from strain Pf-5 (Table S14). Similarly, other phenotypes typically expressed by *Pseudomonas* spp. under the control of the Gac/Rsm signal transduction pathway [61] were not exhibited by Pf0-1 but were exhibited by the *gacA* ^+^-complemented derivative of Pf0-1 (Table S14). From these results, we concluded that the previously-sequenced strain Pf0-1 [32] has a mutation in *gacA*, which encodes a component of the GacA/GacS global regulatory system required for the production of many secondary metabolites and exoenzymes in *Pseudomonas* spp. [61]. Consequently, throughout this study we relied on the *gacA* ^+^ derivative of Pf0-1 to explore relationships between gene inventory and phenotypes for this strain. Although the structures of the CLPs produced by BG33R and Pf0-1 are unknown, the amino acid composition of the peptide moiety could be predicted from the sequences of the NRPSs in the CLP gene clusters. The predicted structure of the BR33R CLP includes a 9-amino acid peptide similar to that of massetolide [59] or pseudophomin A and B [62], and the Pf0-1 CLP includes an 11-amino acid peptide that is distinct from other CLPs described to date (Figure 7).

The fluorescent pseudomonads are characterized by their production of fluorescent pigments in the large and diverse pyoverdine class [63], which function as siderophores for iron acquisition by the bacterial cell. Many genes are involved in the biosynthesis, utilization and regulation of the pyoverdine iron-acquisition system [64], and these *Pseudomonas* spp. have a full complement of pyoverdine genes, which are present in three to seven clusters dispersed in the genomes. Many *Pseudomonas* spp. produce secondary siderophores that also contribute to iron nutrition [64], such as enantio-pyochelin, which is produced by Pf-5 [65]. Among these secondary siderophores is pseudomonine, which is produced by the same NRPS pathway used for the biosynthesis of two other siderophores, acinetobactin and anguibactin, with the primary substrate dictating the final product from a common biosynthetic mechanism [66]. Gene clusters for the biosynthesis and uptake of a pseudomonine-like compound [67] are present in the genomes of BG33R and A506, and clusters for the biosynthesis and transport of the siderophore achromobactin [68] are present in *P. chlororaphis* strains O6 and 30-84. The production of these secondary siderophores has not been confirmed. However, we identified a number of putative binding sites for the ferric uptake regulator (FUR) in the intergenic regions of these gene clusters using HMMs trained on sequences identified in the genome of *P. protegens* Pf-5 [69], suggesting that the genes are iron-regulated, as expected for a siderophore biosynthesis region. In addition, four genomes (Pf-5, BG33R, SBW25 and SS101) have a full complement of genes required for the biosynthesis and efflux of a hemophore (Figure 6), a protein that, when exported from the cell, can chelate heme with high affinity and then be bound and taken up by specific outer membrane receptors [70].

Within the genomes, we identified many orphan gene clusters, defined as loci with characteristic sequences of secondary metabolism genes but without known biosynthetic products. Eight orphan clusters have genes for NRPSs; two have genes for polyketide synthases (PKSs); and one contains a hybrid NRPS-PKS (Figure 6). All strains except Pf-5 have a cluster homologous to *pvfABCD*, which contains an NRPS-encoding gene and is required for the biosynthesis of a putative signaling molecule in *P. entomophila* [71]. A homologous gene cluster (*mgoBCAD*) is required for production of the phytotoxin mangotoxin by strains of *P. syringae* pv. *syringae* causing apical necrosis of mango [72], [73], but the recently-described mangotoxin biosynthesis gene cluster (*mboABCDEF*) [74] is not present in any of the ten genomes of the *P. fluorescens* group. The structure of mangotoxin is not known, but we attempted to detect its production by strains of the *P. fluorescens* group using an established plant bioassay. Mangotoxin-associated phytotoxicity was not observed on tomato leaves inoculated with any of the ten strains. These results agree with a recent report that strains Pf-5 and Pf0-1 do not produce mangotoxin [74]. The functions of the *pvfABCD* homologs in the strains of the *P. fluorescens* group are unknown, but possibilities include a signaling role as proposed for *P. entomophila* [71].

Three of the NRPS-containing orphan gene clusters in the newly-sequenced genomes are likely to encode the biosynthesis of secondary siderophores, based upon similarities to siderophore biosynthetic loci in other bacteria and the presence of genes encoding TonB-dependent outer-membrane proteins, which commonly function in siderophore uptake. One of these, a 36.5-kb region in the genome of BG33R, includes genes for the biosynthesis of salicylic acid, an intermediate in the biosynthesis of pyochelin and other siderophores. Of note, putative FUR binding sites also were identified upstream of several genes within this gene cluster, providing further support for a role of the cluster in iron homeostasis. Bioinformatic analysis of the second putative siderophore-biosynthesis cluster, which is present in Q8r1-96 and SBW25, predicts that the NRPS product is a nine amino acid peptide, possibly ornicorrugatin, which is produced by SBW25 [75]. The NRPS-encoding genes in the third cluster, present in the genome of Q2-87, are predicted to synthesize a six amino acid peptide via a biosynthetic pathway similar to that for siderophore biosynthesis by *Ralstonia eutropha* [76].

*Pseudomonas* spp. are well known for their prolific production of diverse secondary metabolites, only a fraction of which are synthesized via the NRPS and PKS mechanisms of biosynthesis considered in this analysis. Although the products of orphan gene clusters in the seven genomes of this study could not be predicted from the nucleotide sequence data, the loci provide promising subjects for identification of novel natural products. In keeping with the roles of known secondary metabolites produced by these strains, the metabolites could certainly serve important functions in the ecology of these bacteria, including their interactions with other soil- or plant-associated microorganisms.

Of the many secondary metabolite and siderophore biosynthetic gene clusters present in the genomes, only the clusters for pyoverdine production are present in all strains (Figure 6). Certain other clusters (e.g., HCN biosynthesis) are in a conserved location in the genomes of all strains composing a sub-clade, possibly indicating acquisition during the divergence of the sub-clade from its progenitors. Other clusters (e.g., phenazine, 2-hexyl-5-propyl-alkylresorcinol, 2,4-diacetylphloroglucinol, and achromobactin) are present in conserved locations within the genomes of the most closely-related strains within a sub-clade, and may have been acquired more recently in the evolution of those strains. The majority of secondary metabolite gene clusters have a patchy distribution among the ten genomes, indicating a complex pattern of inheritance including several independent acquisition events and/or loss of the clusters from the genomes of certain strains (Figure 6). Therefore, the distribution of secondary metabolism gene clusters in the genomes of these *Pseudomonas* spp. cannot be explained by a single type of inheritance, but results from many processes operating throughout the evolution of these strains ([3] and references therein).

#### Bacteriocins

Among the arsenal of anti-microbials produced by *Pseudomonas* spp. are the bacteriocins, narrow-spectrum proteinaceous toxins that typically kill bacteria closely related to the producing strain. Bacteriocins toxic to bacterial phytopathogens can contribute to biocontrol [77] and can play an important role in the fitness of a strain by killing or inhibiting bacterial co-inhabitants that compete for limited resources in the environment. Each of the ten genomes of the *P. fluorescens* group has two to seven predicted bacteriocins (Figure 6). Collectively, the genomes include genes for many of the structurally-diverse bacteriocins known to be produced by *Pseudomonas* spp., including the S1/2/3/AP41 pyocins [78], [79], S5 pyocins [80], colicin M-like bacteriocins [81], and the lectin-like Llp bacteriocins [38] (Figure 6). Strain A506 has a region related to those encoding microcin B17 production in the Enterobacteria [82]; this bacteriocin has not been described previously in *Pseudomonas* spp. We also identified putative novel bacteriocins in the predicted proteomes of the *P. fluorescens* group by the presence of receptor, translocation, and active domains characteristic of these proteinaceous toxins. One group of putative bacteriocins (designated N1 for novel group 1, Figure 6, Figure S8) has members in all strains studied except for Pf-5. The predicted translocation domain (Pfam: PF06958) shared by proteins in the N1 group is similar to those of other bacteriocins produced by *Pseudomonas* spp., whereas the active and receptor-binding domains are variable. Some members of the N1 group have a DNase domain (Pfam: PF12639) distantly related to those found in pyocins S1/2/AP41, whereas others have a cytotoxic domain (Pfam: PF09000) similar to the active domain found in colicin E3 of *E. coli*, which has RNase activity directed at the 16S ribosomal subunit [83]. This cytotoxic domain is not present in any known bacteriocin produced by *Pseudomonas* spp. The second group of putative bacteriocins (designated N2) is found in four strains (Figure 6, Figure S8). All of the proteins in the N2 group have receptor-binding and translocation (Pfam: PF06958) domains similar to, but distinct from, those in carocin S1, a bacteriocin produced by *Pectobacterium carotovorum* [84]. The active domains are predicted to encode DNase activity; these domains are similar to the active domain of pyocin S3 (∼50% ID) or carocin S1 (∼40% ID) for the N2 proteins of 30-84, O6 and SS101, but similar to those of pyocin S1/S2/AP41 for the N2 proteins in Pf0-1 (Pfam: PF12639). A third predicted type of novel bacteriocin (N3, Figure 6), present in the genome of BG33R, has an active domain similar to the pore-forming domain of colicin N in the C terminus (Pfam: PF01024) but similar to a portion of colicin M at the N terminus [85]. The functions of the diverse bacteriocins present in the genomes of the *P. fluorescens* group remain largely uncharacterized, although enzymatic activity was demonstrated for the colicin M-like bacteriocin from Q8r1-96 [81] and antibacterial activity for an Llp bacteriocin produced by strain Pf-5 [38]. The widespread presence and diversity of these proteinaceous toxins suggest that bacteriocins may play an important role in the intraspecific interactions and competitiveness of *Pseudomonas* spp.

In the genomes of the *P. fluorescens* group, many of the genes coding for bacteriocins are clustered with genes encoding immunity, forming prototypic toxin-antitoxin gene pairs. Others are distal from any known immunity gene, suggesting that immunity may be conferred for multiple related bacteriocins from a single immunity gene or that novel resistance genes may exist in these genomes. There are striking differences among strains in the numbers and types of bacteriocins produced, with no clear correlations to the phylogenetic relationships among the strains. Indeed, many of the bacteriocin genes fall in genomic islands or other atypical regions of the genomes (Figure 6), indicating that these genes may be the result of horizontal mechanisms of inheritance and dispersal.

#### Metabolism of phytohormones, volatiles, and plant signaling compounds

Plant-associated bacteria can influence plant growth and development directly by producing or degrading plant hormones or other factors that modulate plant regulatory mechanisms [7]. Indole-3-acetic acid (IAA) is the primary auxin in plants, controlling many important physiological processes, and IAA production by plant-associated bacteria can have profound effects on plant growth and development [22]. We screened the genomes of the *P. fluorescens* group for pathways involved in the production of IAA [22] and detected genes for tryptophan-2-monooxygenase (IaaM) and indole-3-acetamide hydrolase (IaaH), which convert tryptophan to IAA via the two-step indole-3-acetamide pathway, in the genomes of *P. chlororaphis* strains 30-84 and O6. IAA is known to be produced by strain O6 via the indole-3-acetamide pathway [86] and we detected auxin in cultures of strain O6, as expected; however, we did not detect auxin in cultures of 30-84. Although we detected no obvious mutations in *iaaM* and *iaaH* of strain 30-84, the sequences differ slightly from those in strain O6 (e.g., substitution for a conserved proline at site 80 of IaaH) and may be non-functional. Differences in auxin production also could be due to variation in expression of the IAA biosynthesis genes by the two strains under the conditions of our study. An IAA catabolic (*iac*) gene cluster in the genome of strain BG33R (Figure 6) encodes putative IAA degradation enzymes, a regulatory protein, a dedicated outer membrane porin, and an ABC transporter. The overall genetic organization differs from that of the *iac* cluster of *P. putida* 1290, but resembles a putative IAA degradation locus of *Marimonas* sp. MWYL1 [25]. The cluster resides next to a phage-like integrase gene on genomic Island 3 of BG33R, suggesting that it was acquired via horizontal transfer.

Strains 30-84, O6, and Pf-5 also carry genes for catabolism of the plant hormone and antimicrobial metabolite phenylacetic acid (PAA) [87], [88](Figure 6) and we found that the strains can grow on a medium containing PAA as a sole carbon source. These genes, like the well-characterized *paa* operon of *P. putida* U [89], control conversion of PAA to Krebs cycle intermediates via phenylacetyl-CoA (PAA-CoA) and encode a PAA-CoA ligase, a PAA-CoA oxygenase/reductase, and enzymes catalyzing cleavage and further degradation of the aromatic ring [90]. The *paa* clusters of strains in Sub-clade 1 also include genes encoding components of a PAA-specific transporter.

Aminocyclopropane-1-carboxylic acid (ACC) is the immediate precursor of the plant hormone ethylene. Stressed plants accumulate ethylene, which inhibits root elongation and accelerates abscission, aging and senescence [91]. ACC deaminase-producing rhizobacteria lower plant ethylene levels by converting ACC into ammonia and α-ketobutyrate, thereby stimulating root growth and improving tolerance to environmental or pathogen-induced stress. Among Pf-5 and the seven newly-sequenced strains, only strain Q8r1-96 carries the *acdS* gene, which encodes ACC deaminase. Q8r1-96 grew on DF salts medium [92] with 3 mM ACC as the sole source of nitrogen and produced measurable amounts of α- ketobutyrate (2062.4±539.1 nmol mg protein^−1^ hr^−1^) during deamination of ACC . On the other hand, strains Q2-87 and SS101, which do not have *acdS*, did not grow on the DF-ACC medium and exhibited no detectable ACC deaminase activity.

Acetoin and 2,3-butanediol are volatiles often produced by bacteria during mixed acid-type fermentation. Both compounds have been implicated as plant growth-promoting metabolites [27], [93]. The synthesis of acetoin and 2,3-butanediol is best understood in the Enterobacteriaceae and *Bacillus* spp., where it proceeds via the formation of α-acetolactate from pyruvate and further conversion to acetoin and 2,3-butanediol [24]. The transformations are catalyzed by the catabolic α-acetolactate synthase (BudB/AlsS), α-acetolactate decarboxylase (BudA/AlsD) and acetoin reductase (BudC/YdjL) in members of the Enterobacteriaceae and *Bacillus* spp. [94]–[96]. *P. chlororaphis* O6 is known to produce 2,3-butanediol [27], and a putative acetoin reductase gene is present in the genome of O6 and other strains in Sub-clade 1. However, we did not detect orthologs of *budAB/alsSD*, which catalyze the synthesis of α-acetolactate and acetoin from pyruvate in other bacteria, in the genomes of O6 or 30-84. One plausible explanation for this apparent discrepancy is that α-acetolactate is formed by another pathway in strains O6 and 30-84, possibly via the α-acetohydroxyacid synthase encoded by *ilvBN* [97]. We detected orthologs of *ilvBN* in all ten genomes of the *P. fluorescens* group. α-Acetolactate is unstable and spontaneously decomposes in the presence of oxygen into acetoin or diacetyl (also called 2,3-butanedione) [24], which would provide the necessary substrate for the acetoin reductase and formation of 2,3-butanediol by strains in Sub-clade 1. Six strains featured in this study carry *aco* genes for an acetoin dehydrogenase (AoDH) enzyme complex that converts acetoin to acetaldehyde and acetyl-CoA. A four-gene cluster encoding an AcoR regulatory protein and AcoABC proteins that represent, respectively, the E1α, E1β, and E2 subunits of the AoDH enzyme complex, are present in these genomes. Four strains (Pf-5, 30-84, Q8r1-96,and Q2-87) also have an uncharacterized gene, *acoX*, and a 2,3-butanediol dehydrogenase gene, *bdh*, which may allow catabolism of 2,3-butanediol as well as acetoin. Interestingly, the dedicated E3 (dihydrolipoamide dehydrogenase) component of AoDH is missing from all of the genomes, and a common E3 subunit is presumably shared by AoDH and the pyruvate dehydrogenase and 2-oxoglutarate dehydrogenase enzyme complexes [24].

The non-protein amino acid γ-aminobutyric acid (GABA) is secreted in millimolar amounts by plant tissues in response to abiotic and biotic stresses [98]. This metabolite reduces the activity of herbivorous insects and the virulence of bacterial and fungal pathogens [99]. Indeed, *gabT* mutants of *P. syringae* pv. *tomato* DC3000, which lack production of GABA aminotransferase, exhibit reduced expression of type III secretion and effector genes and reduced virulence in *Arabidopsis* [100]. This observation is consistent with the idea that GABA plays a role in plant-bacterial communication. Genomes of all ten strains included in this study have *gabT* and *gabD*, which encode a putative GABA aminotransferase and a succinate semialdehyde dehydrogenase involved in GABA utilization. Interestingly, the genomes of Q8r1-96, Q2-87, 30-84 and O6 carry three *gabT* paralogs, two of which are linked to *gabD*-like genes. An almost identical *gab* gene arrangement is found in the genome of the plant pathogen *P. syringae* pv. *tomato* DC3000, but a recent study by Park et al. [100] implicated only one *gabTD*-like locus in the catabolism of GABA. The function of GABA in the interactions of biocontrol *Pseudomonas* spp. with their plant hosts remains to be established.

#### Exoenzymes

Secreted enzymes are an important group of molecules involved in nutrient acquisition and the interactions of bacteria with their microbial co-inhabitants and eukaryotic hosts. Each of the ten genomes has a conserved cluster for the exoprotease AprA and its secretion via a type I mechanism (Figure 6). The strains also tested positive for exoprotease production, whereas *aprA* deletion mutants of strains Pf-5 and A506 lacked exoprotease production (Table S14), indicating that the conserved *aprA* gene is responsible for this phenotype. AprA (previously called AprX) production by A506 has a confounding role in the biological control of fire blight disease of pear and apple. The protease degrades pantocin A, a peptide antibiotic produced by the biological control agent *Pantoea vagans* C9-1 that is toxic to the fire blight pathogen *Erwinia amylovora* [101]. AprA-mediated proteolysis of pantocin A results in diminished biological control of fire blight when pome fruits are treated with A506 in combination with *P. vagans* [102]. In contrast, a mixed inoculum composed of the *aprA* mutant of A506 with *P. vagans* results in more effective and consistent biological control of fire blight than achieved with either of the biocontrol strains applied individually. This enhanced biological control is attributed to the combined activity of two compatible biocontrol strains that suppress disease by complementary mechanisms [102]. Seven genomes, representing all three sub-clades, contain additional genes with predicted functions as exoproteases (Figure 6), but their roles in the biology of the strains remain unknown at present.

Chitinases produced by certain *Pseudomonas* spp. can hydrolyze fungal cell walls, thereby contributing to the biological control of fungal diseases of plants [103]. Collectively, the genomes contain two chitinase genes, with one form distributed among strains in all three clades (Figure 6). A second chitinase, which is orthologous to *chiC* of *P. aeruginosa* [104], is present in a region with unusual trinucleotide composition in the three strains in Sub-clade 1, suggesting recent acquisition by this lineage. We evaluated all ten strains for chitinase production, and found that strains having at least one of these chitinases exhibited chitinolytic activity in culture (Table S14).

One strain, SBW25, exhibited pectolytic activity on potato, and a gene for pectate lyase [105] is present in the genome of SBW25, whereas neither pectolytic activity nor the pectate lyase gene was present in the other genomes (Figure 6).

#### Secretion systems

Many extracellular enzymes are transported out of the cell through type II secretion systems (T2SSs) and, collectively, the ten genomes evaluated in this study have four T2SSs. Three of the T2SSs are related to the Xcp and Hxc systems of *P. aeruginosa* [106], whereas the fourth system, present only in the genomes of Sub-clade 3, is novel. Each genome has one to three T2SSs, and candidate substrates include lipase, esterases, alkaline phosphatases, and, in SBW25, a pectate lyase.

Type III and Type VI secretion systems, which function in the delivery of effector molecules into plant, animal, or bacterial cells, are prevalent in Gram-negative bacteria, including environmental strains of *Pseudomonas* spp. having no known pathogenic or symbiotic associations with eukaryotic cells [34], [69], [107]–[109]. We identified several types of both secretory systems in the genomes of the plant-associated strains of the *P. fluorescens* group.

**Figure 8:**
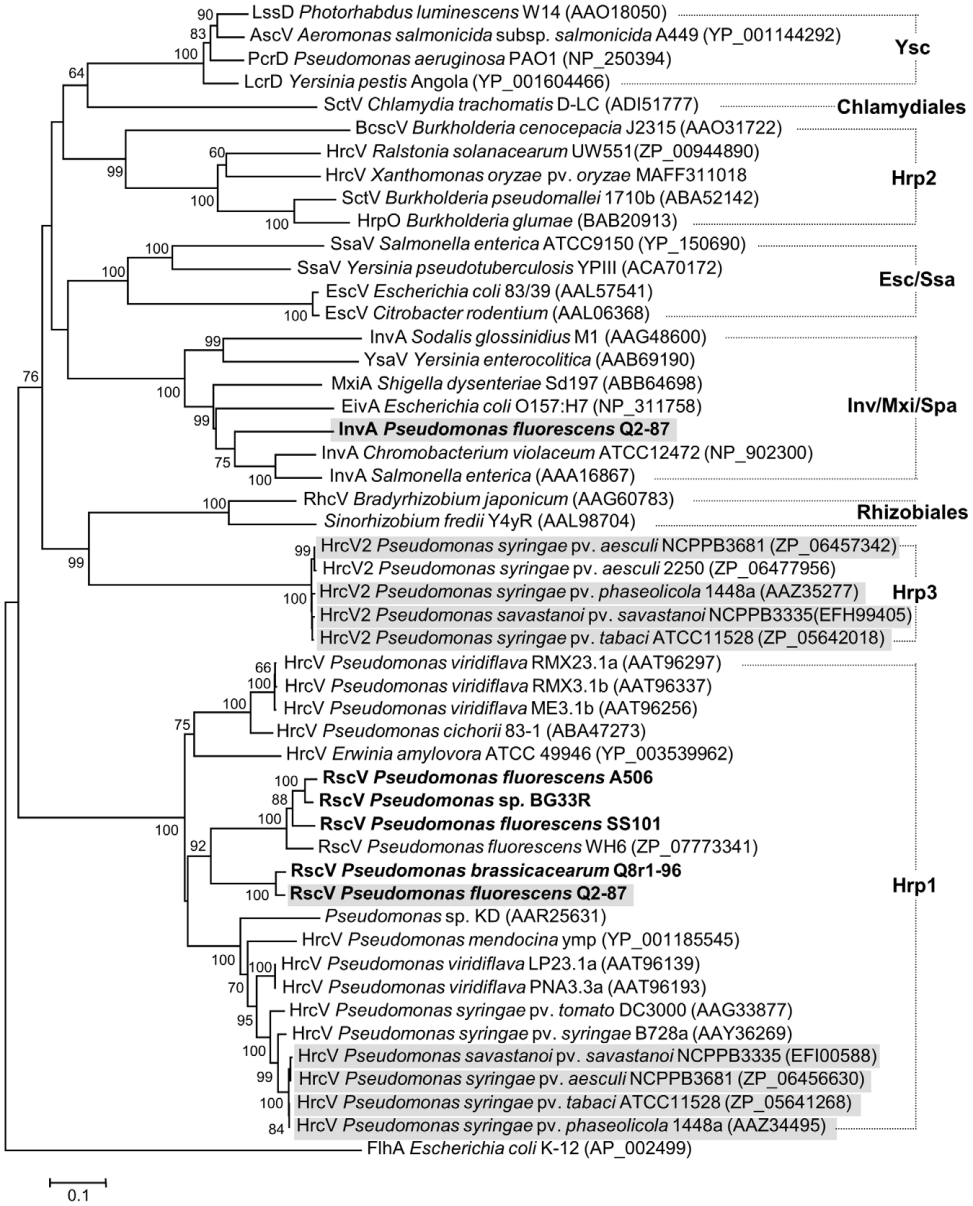
Neighbor-joining phylogeny inferred from aligned amino acid sequences of Hrc(Rsc)V proteins. *Pseudomonas* strains with genomes sequenced in this study are highlighted in boldface, whereas strains carrying two different type III secretion systems are shaded in gray. GenBank accession numbers are shown in brackets. Families of T3SSs are labeled according to Troisfontaines and Cornelis [112]. Flagellar export pore protein FlhA from *E. coli* was used as an outgroup. Indels were ignored during analyses. Evolutionary distances for Hrc(Rsc)V proteins were estimated using the Jones-Taylor-Thornton (JTT) model [177] of amino acid substitution. Bootstrap values equal to or greater than 60% are shown, and the scale bar represents the number of substitutions per site. Branch lengths are proportional to the amount of evolutionary change.

The type III secretion system (T3SS) is used by a variety of Gram-negative bacteria for delivery of effector molecules into a eukaryotic host cell [110]. Six strains examined in this study (i.e. A506, Q8r1-96, Q2-87, SS101, SBW25 and BG33R) carry *rsp/rsc* (rhizosphere-expressed secretion protein and rsp- conserved) gene clusters that vary in length between 18 and 28 kb and resemble the *hrc/hrp* T3SS of the plant pathogen *P. syringae*. The *rsp/rsc* clusters of these six strains belong to the Hrp1 family (Figure 8), which includes T3SSs from pathogenic and saprophytic plant-associated *Pseudomonas* spp. The Hrp1 family is phylogenetically diverse and encompasses multiple lineages of T3SSs that are often encoded by genomic islands [108], [111]. The T3SSs of strains in Sub-clades 2 and 3 are integrated into different sites in the genomes and differ in the arrangement of genes within the *rsc/rspZ* operon. These T3SSs may represent independent acquisitions in the two sub-clades and may relate to sub-clade-specific host or biocontrol properties. No T3SS was detected in the genomes of Sub-clade 1.

Q2-87 is unique amongst the biocontrol strains in possessing a second T3SS gene cluster, in addition to *rsp/rsc*, that is similar to the *inv/mxi/spa* cluster of human and animal pathogens such as *Salmonella enterica* [112]. A type III effector gene in the *inv/mxi/spa*-like gene cluster of *S. enterica* also is present in the Q2-87 genome. This is the first example of an *inv/mxi/spa*-like T3SS in *Pseudomonas* spp.

We identified putative Type III effectors in genomes of several strains in the *P. fluorescens* group through bioinformatic analyses (Figure 6). Of the three effectors shown previously to be secreted by Q8r1-96, two are homologs of the *P. syringae* effectors HopAA1-1 and HopM1; the third, RopB, is a novel effector [108]. Homologs of RopAA-1, RopM and RopB also are present in the genome of Q2-87. A homolog of ExoU, a *P. aeruginosa* effector with phospholipase activity that causes rapid death in eukaryotic cells [113], is present in all strains with T3SSs except for strain Q8r1-96. The A506, SS101 and BG33R genomes have 14 to 15 additional genes preceded by putative Hrp(Rsp)L-dependent promoters; Q8r1-96 also contains one such gene. These genes encode conserved hypothetical proteins with N-termini typical of T3SS-secreted proteins (i.e., abundance of Ser and polar residues at the N-termini, only one acidic residue in the first 12 positions, and an aliphatic amino acid in position 3 or 4)(Table S15, Figure 6). Clearly, experimental evidence is required to prove/disprove the possibility that these proteins indeed represent novel type III effectors.

Despite the widespread occurrence and high level of conservation of T3SSs in strains of the *P. fluorescens* group, their functions remain enigmatic. In SBW25, the T3SS has been shown to operate in the sugar beet rhizosphere and its inactivation compromised the ability of this strain to efficiently colonize plant roots [109],[114],[115]. On the other hand, the T3SSs of *P. brassicacearum* strain Q8r1-96 and *P. fluorescens* KD are expressed during root colonization, but the corresponding mutants are not altered in their rhizosphere competence [108], [116]. In environmental strains of *P. aeruginosa*, ExoU and other T3SS effectors are required for colonization and killing of protozoa [117]. Similarly, the T3SSs of *P. fluorescens* may function in defense of the bacteria against predation and competition in their natural habitats in the soil, rhizosphere, or on aerial plant tissues. In line with this possibility, the T3SS of *P. fluorescens* strain KD is involved in suppression of *Pythium ultimum*, a soilborne oomycete pathogenic to many plant species [116].

Type VI secretion systems (T6SSs) are prevalent and conserved among Gram-negative bacteria. During the first years after their discovery, they were thought to be involved primarily in delivery of virulence effectors to eukaryotic hosts. More recently, the prevalence of T6SSs in genomes of environmental bacteria, which are likely to encounter intense competition and predation in natural habitats, has become increasingly evident; and T6SSs are now thought to play a role in interbacterial interactions [118]–[120]. Each of the genomes of the *P. fluorescens* group includes one to three clusters of genes encoding a T6SS. Collectively, the genomes include four types of T6SSs, three of which are similar to the three well-characterized T6SS loci of *P. aeruginosa* (termed HSI-I, HSI-II and HSI-III) [121]. A locus similar to the HSI-I T6SS, which was described previously in strain Pf-5 [69], is present in nine of the genomes evaluated herein; only strain Pf0-1 lacks an ortholog of HSI-I (Figure 6). These HSI-I loci lack *tagJ1*, which encodes an accessory lipoprotein in the *P. aeruginosa* HSI-I, and four genomes have two additional genes at this location in the gene cluster. Seven genomes (30-84, O6, Q2-87, SS101, Pf0-1, A506, and BG33R) have loci similar to HSI-II, and SBW25 has an incomplete copy of an HSI-II-like T6SS. The HSI-II loci have several gene substitutions relative to the locus in *P. aeruginosa*. Strains A506, SS101 and BG33R lack *clpV*, but contain a gene encoding an Hcp family protein, which is absent from the rest of the genomes. In contrast to *P. aeruginosa*, the HSI-II loci of *P. chlororaphis* 30-84 and O6 include several genes encoding hypothetical proteins and a PAAR motif protein similar to *evpJ*, a non-essential gene found in the T6SS of *Edwardsiella tarda* [122]. A third locus, in the genomes of strains Q2-87, Q8r1-96 and Pf0-1, contains genes encoding all of the necessary components of the HSI-III T6SS of *P. aeruginosa* but lacks an ortholog of PA2372, which is not essential to the function of the transport system [123]. *P. chlororaphis* O6 has a fourth T6SS related to a T6SS locus (y3658–y3677) in *Yersinia pestis* strain Kim [124] and *tss-*4 from *Burkholderia pseudomallei* strain K96243 [125]. This T6SS is in a region of the O6 genome with atypical trinucleotide composition that is flanked by transposases, indicating that it may have been recently acquired. Effector proteins delivered by the T6SSs of *P. fluorescens* or *P. chlororaphis* are unknown and orthologs of Tse1, Tse2, and Tse3, which are secreted via the H1-T6SS of *P. aeruginosa* [119], were not found in these genomes.

#### Insect toxicity

Certain strains in the *P. fluorescens* group are toxic to insects and, in some cases, this toxicity is associated with gene clusters encoding the Mcf (makes caterpillars floppy) toxin or Tc (toxin complexes) first described in insect pathogens such as *Serratia entomophila* and bacterial endosymbionts of entomopathogenic nematodes such as *Photorhabdus* spp. and *Xenorhabdus* spp. [39], [126]–[128]. *fitD* (*fluorescens* insect toxin), which is closely related to *mcf*, is present in the genome of *P. protegens* Pf-5 and associated with that strain's lethality against the tobacco hornworm *Manduca sexta* [39]. The *fitABCDEFGH* locus, which includes genes for regulation and efflux of the FitD toxin [39], is located within a 90-gene insertion into the genome of Pf-5, portions of which have features (phage integrase and phage remnants, unusual nucleotide composition) indicative of horizontal acquisition. *P. chlororaphis* strains O6 and 30-84 also have complete *fitABCDEFGH* loci that are part of 24–28 gene insertions into the same location in both genomes. Genes distantly related to *fitD* (27–28% identity) are present in the genomes of Q8r1-96, Q2-87, and Pf0-1, but other genes of the *fit* locus are not present in these strains.

Whereas the *fit* cluster is present only in members of Sub-clade 1, loci similar to the Tc clusters are present in seven of the ten genomes including representatives of each sub-clade. Collectively, the genomes have six distinct types of Tc clusters distinguished by the number and organization of component genes and the location of the clusters in the genomes. *Pseudomonas* sp. BG33R contains two Tc clusters, which have distinct compositions and are located distally in the genome. The cluster in *P. chlororaphis* 30-84 is flanked by phage integrases within a unique 74.1-kb region (Island 2; Table S13) having a transposase at one terminus. These features, along with the unusual GC content and trinucleotide composition, suggest a horizontal mechanism of inheritance. Tc clusters appear to be widely distributed in bacterial genomes, and their functions in the ecology of the producing strains remain largely unknown. To date, a role for these genes in *Pseudomonas* spp. has been established only for the *tccC* gene from *Pseudomonas taiwanensis*, which confers an insect lethality phenotype when expressed in *E. coli* [128]. Insect toxicity has been reported for Pf-5 [39], [127], but is not known for any of the other strains of this study, and the potential roles of toxins and other phenotypes in the interactions of these bacteria with insects is an intriguing area for future study.

#### Correlating phylogenies with metabolic profiles and gene inventories

The diversity of bacteria within the *P. fluorescens* group has been recognized for many decades, and a polyphasic approach including catabolic profiles has been used to classify these bacteria since the 1960s [129]. We conducted catabolic profiling assays using the Biolog Phenotype Microarray (PM) system and found that, despite their genomic diversity, the strains displayed similar core carbon metabolic profiles (Figure S9). In contrast, the strains differed in some of their subsidiary catabolic abilities, particularly in utilization of plant-derived compounds. For instance, sucrose, D-tartaric acid and M-tartaric acid were utilized by several strains, whereas L-tartaric acid was not utilized. Only Pf0-1 utilized citraconic acid; only A506 utilized 2-deoxy-D-ribose; and only SBW25 and Q8r1-96 utilized L-homoserine. From these catabolic profiles, additional phenotypic characterization of the strains, and bioinformatic analyses of the ten genomes, we identified genes correlated with many of the traits used for classification of species, sub-species and biovars within the *P. fluorescens* group (Table S16).

All strains in Sub-clade 1 (Figure 1) have clusters for utilization of phenylacetic acid, benzoate, and trehalose, which are characteristics of *P. chlororaphis* and the newly-described species *P. protegens*, to which Pf-5 has been assigned [31]. Strains 30-84 and O6 each have a levan sucrase gene, clusters for phenazine biosynthesis and an L-arabinose transporter, whereas Pf-5 lacks these loci; lack of phenazine production, levan sucrase activity and L-arabinose assimilation are among the phenotypes differentiating Pf-5 from *P. chlororaphis* subsp. *aureofaciens* [31], [130] (Table S16). Strain O6 has a cluster encoding a nitrate reductase and nitrite transporter system, and we found that it could reduce nitrate to nitrite. In contrast, strain 30-84 lacks the gene cluster and the nitrate-reducing activity, a phenotype known to vary among strains of *P. chlororaphis* subsp. *aureofaciens* [130]. Additionally, D-serine was utilized by the two *P. chlororaphis* strains, the only two strains in this analysis that contain a D-serine ammonia lyase gene and an adjacent D-serine deaminase transcriptional activator.

Within Sub-clade 2, the genomes of Q2-87 and Q8r1-96 have a full complement of genes for denitrification, and we detected complete reduction of nitrate by both strains. The strains have genes or gene clusters for levan sucrase and the utilization of ethanol, sorbitol, and mannitol; they also utilized these compounds as sole carbon sources (Figure S6, Table S16). These phenotypes are characteristic of *P. brassicacearum*, a species of root-associated bacteria that, like Q2-87 and Q8r1-96, produces 2,4-diacetylphloroglucinol [131] and fits into the *P. corrugata* sub-group defined by Mulet et al. [13]. A very close relationship between *P. brassicacearum* NFM421 and strain Q8r1-96 also was revealed in the phylogenies based on the full genome sequences of these strains, whereas *P. brassicacearum* NFM421 appears more distantly related to strain Q2-87 (Figure S2). Consequently, we adopted the species designation of *P. brassicacearum* for strain Q8r1-96. In contrast to strains Q2-87 and Q8r1-96, Pf0-1 lacks genes for nitrate reduction, levan sucrase, and utilization of *myo*-inositol, sorbitol, and ethanol, and is likely to fall into a separate sub-group within the *P. fluorescens* group. Again, these gene inventories were congruent with the phenotypes exhibited by Pf0-1 in our assays (Table S16).

Three of the four strains in Sub-clade 3 exhibited phenotypes typical of *P. fluorescens* Biovar I [129], testing positive for levan sucrase, utilization of sorbitol, L-arabinose, xylose, L-tryptophan, and adonitol, but lacking the capacity to reduce nitrate. The fourth strain, BG33R, shared all of these phenotypes except for levan sucrase production and therefore falls into Biovar V-6, a group whose commonalities with Biovar I have been noted previously [132]. We made putative links between the use of each of these carbon sources and specific catabolic genes in each of these strains. Therefore, by coupling the genomic analysis with phenotypic tests, we identified a set of traits and gene inventories that are useful in differentiating the strains in a manner congruent with their phylogenies (Table S16, Figure S10).

#### Conclusions

It appears that *Pseudomonas* spp. occupy varied niches by virtue of an expanded pan-genome, with the variable genome providing functions that tailor fitness to specialized habitats occupied by a subset of strains. At 13,872 genes, the pan-genome of ten plant-associated strains within the *P. fluorescens* group makes up a substantial portion of the pan-genome of the genus as a whole, which was estimated at 25,907 in this study. Comparisons of many of the sequenced strains of *Pseudomonas* spp. (Figure 1) identified a core genome of only 1491 genes, representing less than 35% of any individual genome, further emphasizing the heterogeneity of the genus and the important role of the variable genome in tailoring individual strains to their specific lifestyles. This heterogeneity was highlighted further by the discovery that only 20 genes are shared by all strains within the *P. fluorescens* group and absent from the genomes of other *Pseudomonas* spp., suggesting that gene flow among *Pseudomonas* spp. is a significant factor modulating gene inventory. Although the ten strains in this study exhibit many commonalities with respect to their plant commensal lifestyle, their genetic repertoires are varied and plastic. There are clearly multiple pathways to success with respect to establishing bacterial populations on plant surfaces.

Comparison of ten genomes within the *P. fluorescens* group provided ample evidence that the tremendous ecological and physiological diversity of these bacteria extends to the genomic level. Genomic diversity commensurate with the biological diversity of the *P. fluorescens* group also was recognized in earlier studies comparing the genomes of strains sequenced previously, leading Silby et al. [29], [31] to propose that these strains fall into a species complex. Here, we defined three sub-clades within the *P. fluorescens* group on the basis of phylogenies inferred from MLSA and genomic comparisons between ten strains in the group. Distinctions between the three lineages were supported by a number of criteria such as genomic synteny, sizes of the lineage-specific core genomes, and gene inventories. As genome sequences for additional strains within the *P. fluorescens* group are incorporated into these phylogenetic analyses in the future, the number of lineages undoubtedly will increase. For example, Pf-5 and Pf0-1 are not as closely related to other members of their sub-clades as those members are to one another, and it is likely that these strains reside in distinct lineages that will become more defined as genomes for sister strains become available in the future. Alternatively, distinctions between the three lineages defined here may become blurred as more genomes are added. Recently, genomes of many other plant-associated strains within the *P. fluorescens* group have been published [133]–[136], and many more are likely to become available in the near future. Although we retained most species names that have been published previously for the strains included in this study, our genomic analysis highlighted discrepancies in the taxonomy of the *P. fluorescens* group and we recognize that some species designations are likely to change in the future. The need for taxonomic revision within the *P. fluorescens* group is well recognized, and the findings of this study illustrate the important role that comparative genomics is likely to play in defining the relationships between strains comprising this heterogeneous group of bacteria.

Like other *Pseudomonas* spp., strains within the *P. fluorescens* group have large genomes conferring an extensive functional repertoire. In other bacterial genera, genomes are smaller in pathogenic vs. environmental isolates or strains. In contrast, there is no striking pattern correlating genome size to a known pathogenic vs. saprophytic lifestyle in *Pseudomonas* spp. Plant pathogenic strains of *P. syringae*, opportunistic human pathogens of *P. aeruginosa*, and the insect pathogen *P. entomophila* have genome sizes ranging from 5.9 Mb to 6.9 Mb. Therefore, rather than exhibiting a reduced genome size reflecting a specialized pathogenic lifestyle, genomes of these known pathogens within the *Pseudomonas* spp. are similar in size to those of environmental isolates. This similarity is not surprising given that plant pathogenic strains of *P. syringae* are known to live epiphytically on plant surfaces and *P. aeruginosa* can be isolated from soil, water and other environmental substrates, indicating that pathogenesis is only one aspect of the lifestyle of these species. The varied functions conferred by a large genome appear to be required by members of the genus to handle the range of environments that these bacteria encounter.

This study included a survey of the genomes for traits associated with biological control and other multitrophic interactions of the *P. fluorescens* group with plants, microbes, and insects. The distribution of these traits was superimposed on maps defining the ancestral and recently-acquired regions of each genome to develop a view of the evolution of these traits in the *P. fluorescens* group. Regions containing core CDSs shared among all strains, which comprise 45% to 52% of each predicted proteome, represent the more ancestral components of each genome. Almost all of the traits associated with biological control or other multitrophic interactions map to genomic regions present in only a subset of the strains or unique to a specific strain (Figure 3). This finding is consistent with the established literature, which provides numerous examples of strain specificity related to biological control activity. Certain traits (e.g., HCN production) are associated with specific sub-clades, possibly reflecting an ancestral status within specific lineages (Figure 6). Most of the traits have a patchy distribution among the strains, and loci for many of these traits were probably acquired through horizontal gene transfer. A fraction of the identified traits (e.g., certain bacteriocins and Tc insect toxins) are encoded by genes located in clearly-defined MGEs (Table S13), but genes encoding the vast majority of biocontrol traits map outside of the MGEs to other regions of the variable genome. Some of these loci have characteristics suggesting recent acquisition, such as atypical GC content and trinucleotide composition and the lack of REP elements. Other loci map to regions that are similar to the core genome in these respects, possibly reflecting acquisition from related bacteria with similar GC content and trinucleotide skew, acquisition in the distant past with subsequent alteration in sequences, or vertical inheritance accompanied by subsequent deletion from certain strains. On the other hand, many of the variable genomic regions may have resulted from horizontal transfer of sequences, other than defined MGEs, that were introduced into the cell and became integrated through recombination or other mechanisms independent of transposons or integrons. Indeed, one role proposed for REP elements is as sites for homologous recombination [137]. This mechanism of evolution appears particularly likely in the genomes of the *P. fluorescens* group described herein, as many of the genes unique to each genome are present as singles, doubles or large groups inserted into core regions lacking any diagnostic features of MGEs. While the specific mechanisms for inheritance of biocontrol traits are obscure, the findings of this study underscore the exclusive occurrence of many traits in specific strains or sub-clades, which is consistent with the strain-specificity of biological control that has been observed for decades.

The variable regions of the *P. fluorescens* group genomes represent valuable resources for future discovery of new aspects of the biology of these bacteria and their interactions with other organisms. A case in point is provided by strain Pf-5, as the genomic sequence data have facilitated the discovery of four novel traits with potential roles in biological control. These are the cyclic lipopeptide orfamide A [35]; the bacteriocin LlpA [38]; analogs of rhizoxin [36], a macrolide that inhibits microtubule assembly in eukaryotic cells; and the FitD insect toxin [39]. An emphasis of this study was to associate variations in the genomes of strains within the *P. fluorescens* group to phenotypes key to the metabolism or lifestyle of these bacteria. Coupling comparative genomics with phenotype testing, we confirmed many of the known phenotypes of the biological control strains. More importantly, novel gene clusters were identified in each strain, providing opportunities for future exploration of unknown mechanisms by which these bacteria interact with their co-inhabitants, plant hosts and other organisms in the natural environment.

## Materials and Methods

### Selection and characterization of strains

Seven strains were selected for genomic sequencing based upon their characterized and distinctive biological control properties and their isolation from different habitats (bulk or rhizosphere soil or aerial plant surfaces) (Table 1). The seven strains and three previously-sequenced strains (Pf-5, Pf0-1, and SBW25) evaluated in this study exhibited the conserved phenotypes of the P. *fluorescens* group: positive for fluorescence under UV light, arginine dihydrolase activity, and oxidase activity; and negative for growth at 41°C and induction of a hypersensitive response on tobacco, determined through standard methods [138] (Table S16). The ten strains were subjected to a panel of biochemical and biological assays (nitrate reduction, levan sucrase production, potato soft rot, gelatinase activity, and catabolic spectra) [138], [139] to assign each to a biovar of *P. fluorescens* or to a species of *Pseudomonas* [129] (Table 1 and Table S16). Strains A506, 30-84, SS101, and BG33R are rifampicin-resistant (100 µg/ml) derivatives of field isolates; previously, spontaneous mutants with resistance to rifampicin were selected to facilitate tracking of these strains in field studies. Strain A506 is known to have a single nucleotide insertion in *rpoS*, which causes a frameshift resulting in a truncated form of the stationary-phase sigma factor RpoS [140]. During the course of this work, we discovered that strain Pf0-1 has a mutation in *gacA*, which encodes a component of the GacA/GacS global regulatory system in *Pseudomonas* spp. [61]. We sequenced *gacA* and *gacS* from the strain Pf0-1 in our collection and confirmed that the sequences are identical to those in the published genome of Pf0-1 [32]. It is not possible to know whether the mutations in A506 and Pf0-1 were present in the strains prior to isolation or if they developed in the laboratory during storage, but all strains have been maintained as frozen stocks (−80°C) throughout this study and for many years preceding.

### Genome sequencing

The genome sequences were determined using shotgun sequencing with a combination of Sanger sequencing (to 4× coverage of the genome size) and 454 pyrosequencing technologies with paired end reads [141], [142]. A hybrid genome assembly was prepared from these datasets using Newbler 2.3 (Roche) and Celera Assembler 5.42. Each genome was subsequently evaluated for additional assembly improvement with the Celera Assembler 5.42 assembly versions providing the starting points. Multiple gaps were closed by merging overlapping contigs and resolving repetitive gaps. Further physical and sequencing gaps were closed by sequencing of PCR products spanning the gaps. The order of scaffolds in the genomes of strains 30-84 and Q8r1-96 (Figure 3) was confirmed by PCR.

### Accession numbers

The whole genome shotgun sequencing projects have been deposited at DDBJ/EMBL/GenBank under the accessions AHHJ00000000 (30-84), AHOT00000000 (O6), AGBM00000000 (Q2-87), AHPO00000000 (Q8r1-96), AHPP00000000 (BG33R) and AHPN00000000 (SS101). Accession numbers for the complete genome sequences are: CP003041 for the chromosome of A506 and CP000076 for the updated genome sequence of Pf-5.

### Bioinformatic analysis

Identification of putative protein-encoding genes and annotation of the genomes were performed as previously described [143]. A set of open reading frames predicted to encode proteins was initially identified using GLIMMER [144]. Open reading frames consisting of fewer than 30 codons and those containing overlaps were eliminated. Functional assignment, identification of membrane-spanning domains, determination of paralogous gene families, and identification of regions of unusual trinucleotide composition were performed as previously described [143]. The annotation of each of the genomes has undergone significant manual curation, removing small spurious overlapping ORFs and improving gene function calls. Manual curation of the genomes was performed using the MANATEE program (http://manatee.sourceforge.net/jcvi/index.shtml). The annotation of the previously-published genome of Pf-5 was updated and manually curated as part of this study. Phylogenetic analyses on the pyocin proteins were performed using MEGA 5 [145]; domain analyses were performed using the InterProScan program, found on the InterPro website [146]. Secondary metabolite production clusters were examined using the antiSMASH program [147]. The amino acid composition of products from NRPS sequences were predicted using NRPSpredictor 2 [148]. Transposons were identified using the ISfinder database (http://www-is.biotoul.fr/) [149]; only expectation values of 10^−5^ and below were considered as significant matches during searches. The *Pseudomonas* genome database [150] was consulted to obtain information on previously-published genomes for comparative purposes.

The seven genomes were compared to other genomes of *Pseudomonas* species using a multiway BLASTp analysis, and putative orthologs were identified with an E-value cutoff of 10^−5^. Synteny analyses were performed using Progressive MAUVE [151]. Phylogenetic relationships among all sequenced *Pseudomonas* species were investigated by generating phylogenetic trees with MrBayes 3.1.2 [152] using 1) 16S rRNA and 2) concatenated alignments of 10 highly conserved housekeeping genes: *acsA*, *aroE*, *dnaE*, *guaA*, *gyrB*, *mutL*, *ppsA*, *pyrC*, *recA*, and *rpoB*. We also used Hal, a Markov Clustering algorithm based on e-values from reciprocal all-by-all BLASTP analysis [153], to determine phylogenetic relationships among the sequenced strains of *Pseudomonas* spp.

REP elements were defined by searching for repeat sequences greater than 30 nt in length that occurred more than 10 times within individual genomes using RepeatScout [154]. Overlapping repeat regions were identified using sequence alignments and assembled to generate consensus repeat motifs. The consensus sequences were used to search the genomes with an identity cut-off of >90%. Sequences identified were aligned using ClustalX and HMMs were generated from alignments using HMMER2.

### Phenotypic assays supporting gene function or biotype designations

Ten strains within the *P. fluorescens* group (Table 1) were tested for phenotypes associated with gene functions. In addition, we tested derivatives of strain Pf0-1 containing cloned *gacA* or *gacS* genes from strain Pf-5. pJEL5965 has a 1.6-kb *Eco*RI/*Hin*dIII fragment containing the *gacA* gene from Pf-5 cloned into pME6000; pJEL5999 has a 6.7-kb *KpnI* fragment containing the *gacS* gene from Pf-5 cloned into pME6000 [155]. Mutants of strains Pf-5 and A506 were included as controls in phenotypic tests, including *gacS* and *aprA* mutants of A506 [101], and ii) *gacA* [69], *ofaA* [69], *chiC*, *hcnB*, and *aprA* mutants of Pf-5. The *hcnB* mutant was created as described for the *ofaA* mutant [69], except that the PCR product was digested with *Hin*dIII and cloned into the *Hin*dIII site of pEX18Tc [156]. The *aprA* and *chiC* mutants contain in-frame deletions and were made using previously-described methods for creating an in-frame deletion [157], except that the PCR products were digested with *Bam*HI or *Xba*I and cloned into the *Bam*HI or *Xba*I site of pEX18Tc [156]. Primers used for mutant construction are listed in Table S17. The deletions were confirmed by PCR amplification and sequencing of the mutant alleles. All phenotypic tests were done on duplicate cultures grown at 27°C (unless another temperature is specified), experiments were repeated, and representative results are presented (Table S12, Table S16).

#### Exoenzymes

Extracellular protease was assessed visually as a cleared zone around bacterial colonies on half-strength BBL Litmus milk agar (Becton, Dickinson and Company, Sparks, MD USA) following incubation for 2 or 4 days. *aprA* mutants of strains A506 [101] and Pf-5 served as negative controls. Gelatinase activity was assessed in 12% gelatin incubated at 20°C and examined at 48 h and 1 week post inoculation [138]. Lipase activity was assessed in LB agar containing 1% w/v Tween 80, added before autoclaving. A positive result was observed as the formation of a white precipitate around a bacterial colony; plates were examined at 48 h and 1 week post inoculation. Chitinase activity was estimated from cultures grown in KB broth for 4 days with shaking using a methylumbelliferone-based chitinase assay kit (Sigma, St. Louis, MO). A chitinase-deficient mutant (*chiC*) of Pf-5 served as a negative control.

#### Secondary metabolites

Cyclic lipopeptide (CLP) production was assessed as surfactant activity in the droplet collapse assay [35] and hemolytic activity, detected as a clearing zone surrounding colonies grown for 48 hr at 27°C on BBL™ Blood Agar Base (Becton, Dickinson and Company, Sparks, MD, USA). CLP production also was visualized as clear zones surrounding colonies grown on CAS agar amended with iron, as described by Hartney et al. [158]. Swarming motility was assessed on standard succinate medium (SSM) [159] containing 0.6% agar following 2 days of incubation at room temperature, as described previously [60]. Mutants deficient in cyclic lipopeptide production serving as negative controls were: an orfamide deficient mutant (*ofaA*) of strain Pf-5 [69], a viscosin-deficient mutant (*viscA*) of strain SBW25 [60], and a massetolide-deficient mutant (*massA*) of strain SS101 [59].

Indole production was assayed in supernatants of cultures of strains in KB broth with 0.2 mg/ml L-tryptophan for 48 h. Salkowski's reagent [160] was added to the supernatants in a 2∶1 ratio and OD_530 nm_ was measured after 30 min incubation at room temperature.

We attempted to detect mangotoxin-associated activity using an established bioassay [72] evaluating symptoms following wound-inoculation of tomato leaves (cultivars Oregon Spring and Legacy).

Hydrogen cyanide production was detected as described by Sarniguet et al. [161]. A mutant of Pf-5 (*hcnB*) deficient in hydrogen cyanide production served as a negative control.

#### ACC deaminase activity

The amount of α-ketobutyrate generated by the enzymatic hydrolysis of 1-aminocyclopropane-1-carboxylic acid in cell-free extracts was monitored as described by Honma and Shimomura [162].

#### Biolog phenotyping and carbon source utilization

Strains of *Pseudomonas* spp. were grown on LB agar at 25°C overnight. Cells were inoculated into 1× IF-0 media (Biolog, Inc., Hayward, CA, USA) and the transmittance of the suspension measured using a Biolog Turbidimeter (Biolog, Inc.). Cells were added until a uniform suspension of 42% transmittance was achieved. The cell suspension was added to 1× IF-0 media containing Dye A (Biolog, Inc.) in a ratio of 1∶5 to produce a cell suspension with a final transmittance of 85%. 100 µl of cell suspension was transferred to each well of Biolog plates PM01 and PM02A (Biolog, Inc.). Plates were incubated using the OmniLog Phenotype MicroArray System (Biolog, Inc.) at 25°C for 48 h, with measurements recorded at 15 min intervals. Data was visualized using OmniLog File Management/Kinetic Analysis software v1.20.02 and analyzed using OmniLog Parametric Analysis software v1.20.02 (Biolog, Inc.). The total area under the curve was used to compare strain phenotypes.

Growth on selected compounds as sole carbon sources was tested on minimal medium 925 [163] amended with the compounds at 0.1% w/v, unless otherwise noted.

## Supporting Information

Figure S1 Phylogenetic tree depicting the relationships among sequenced strains of *Pseudomonas* spp. The tree is based on 16S rDNA alignments and was generated using the MrBayes package [152]. The interior node values of the tree are clade credibility values, which represent the likelihood of the clade existing, based on the posterior probability values produced by MrBayes.
(TIF)

Figure S2 Phylogenetic tree depicting the relationships among sequenced strains of *Pseudomonas* spp. This maximum likelihood tree is based on the concatenated alignments of 726 shared proteins found within all of the genomes and was generated using the Hal pipeline [153]. The interior node values of the tree are representative of the number of bootstraps out of 100.
(TIF)

Figure S3 Chromosomal alignments of strains within Sub-clade 1 generated using Progressive MAUVE [151]. (A) *P. protegens* Pf-5, *P. chlororaphis* 30-84 and *P. chlororaphis* O6, (B) the *P. chlororaphis* strains only. Regions of significant synteny between the strains are shown as colored blocks in the mauve alignment. Regions of sequence not shared between the strains are seen as white gaps within the blocks or spaces between the blocks. Colored lines connect syntenous blocks of sequence between the strains. Breaks between scaffolds are designated by vertical red lines extending through and below the blocks of a genome (30-84 and O6). The tree on the left hand side of (A) shows the relatedness of the strains as determined by MSLA (Figure 1).
(TIF)

Figure S4 Chromosomal alignments of strains within Sub-clade 2 generated using Progressive MAUVE [151]. (A) *P. fluorescens* Pf0-1, *P. fluorescens* Q2-87, and *P. brassicacearum* Q8r1-96 and (B) *P. brassicacearum* Q8r1-96 and *P. fluorescens* Q2-87 only. Regions of significant synteny between the strains are shown as colored blocks in the mauve alignment. Regions of sequence not shared between the strains are seen as white gaps within the blocks or spaces between the blocks. Breaks between scaffolds are designated by vertical red lines extending through and below the blocks of each genome. Colored lines connect syntenous blocks of sequence between the strains. The tree on the left hand side of (A) shows the relatedness of the strains as determined by MSLA (Figure 1).
(TIF)

Figure S5 Chromosomal alignments of strains within Sub-clade 3 generated using Progressive MAUVE [151]. Regions of significant synteny between the strains (*P. fluorescens* SBW25, *Pseudomonas* sp. BG33R, *P. fluorescens* A506 and *P. fluorescens* SS101) are shown as colored blocks in the mauve alignment. Regions of sequence not shared between the strains are seen as white gaps within the blocks or spaces between the blocks. Breaks between scaffolds are designated by vertical red lines extending through and below the blocks of genome BG33R. Colored lines connect syntenous blocks of sequence between the strains. The tree on the left hand side of the figure shows the relatedness of the strains as determined by MSLA (Figure 1).
(TIF)

Figure S6 REP frequency. Local spacing of REPa sequence elements. The frequency of the distance (bp) between adjacent REPa sequences separated by fewer than 200 bp is shown for each of the seven newly-sequenced strains. Distances were measured from the center of REPa sequences.
(TIF)

Figure S7 Similarities between cargo genes in different mobile genetic elements present in genomes of the *P. fluorescens* group. The level of similarity is depicted by the strength of grey shading in boxes representing overlap between two elements. Abbreviations: pro (prophage); isl (island); plas (plasmid); tn (transposon). +^ni^ = integrase present and not intact; + = integrase present and intact; − = integrase not present. Data for Pf-5 was published previously [54]; SBW25 and Pf0-1 were not examined in this analysis.
(PDF)

Figure S8 Phylogenetic tree depicting the relationships of pyocin-like bacteriocins found in genomes of the *P. fluorescens* group. Translocation domains (Pfam: PF06958) were used for this analysis; they are the most conserved domain in the pyocin-like proteins. Proteins found within the ten genomes examined in this study are bolded; characterized proteins are italicized. Interior node values of the tree are representative of the number of bootstraps out of 1000. Color coding is as follows: bacteriocin group N1 (Red), group N2 (Dark blue), carocin (Light blue), Pyocin S1/2/AP41-like (Green).
(TIF)

Figure S9 Kinetic curves depicting rates of respiration of strains in the *P. fluorescens* group grown in Biolog PM carbon utilization plates PM01 and PM02. Substrates are as follows: PM01: A01: Negative Control; A02: L-Arabinose; A03: N-Acetyl-D-Glucosamine; A04: D-Saccharic Acid; A05: Succinic Acid; A06: D-Galactose; A07: L-Aspartic Acid; A08: L-Proline; A09: D-Alanine; A10: D-Trehalose; A11: D-Mannose; A12: Dulcitol; B01: D-Serine; B02: D-Sorbitol; B03: Glycerol; B04: L-Fucose; B05: D-Glucuronic Acid; B06: D-Gluconic Acid; B07: D,L-α-Glycerol-Phosphate; B08: D-Xylose; B09: L-Lactic Acid; B10: Formic Acid; B11: D-Mannitol; B12: L-Glutamic Acid; C01: D-Glucose-6-Phosphate; C02: D-Galactnoic Acid-γ-Lactone; C03: D,L-Malic Acid; C04: D-Ribose; C05: Tween 20; C06: L-Rhamnose; C07: D-Fructose; C08: Acetic Acid; C09: α-D-Glucose; C10: Maltose; C11: D-Melibiose; C12: Thymidine; D01: L-Asparagine; D02: D-Aspartic Acid; D03: D-Glucosaminic Acid; D04: 1,2-Propanediol; D05: Tween 40; D06: α-Keto-Glutaric Acid; D07: α-Keto-Butyric Acid; D08: α-Methyl-D-Galactoside; D09: α-D-Lactose; D10: Lactulose; D11: Sucrose; D12: Uridine; E01: L-Glutamine; E02: M-Tartaric Acid; E03: D-Glucose-1-Phosphate; E04: D-Fructose-6-Phosphate; E05: Tween 80; E06: α-Hydroxy Glutaric Acid-γ-Lactone; E07: α-Hydroxy Butyric Acid; E08: β-Methyl-D-Glucoside; E09: Adonitol; E10: Maltotriose; E11: 2-Deoxy Adenosine; E12: Adenosine; F01: Glycyl-L-Aspartic Acid; F02: Citric Acid; F03: M-Inositol; F04: D-Threonine; F05: Fumaric Acid; F06: Bromo Succinic Acid; F07: Propionic Acid; F08: Mucic Acid; F09: Glycolic Acid; F10: Glyoxylic Acid; F11: D-Cellobiose; F12: Inosine; G01: Glycyl-L-Glutamic Acid; G02: Tricarballylic Acid; G03: L-Serine; G04: L-Threonine; G05: L-Alanine; G06: L-Alanyl-Glycine; G07: Acetoacetic Acid; G08: N-Acetyl-β-D-Mannosamine; G09: Mono Methyl Succinate; G10: Methyl Pyruvate; G11: D-Malic Acid; G12: L-Malic Acid; H01: Glycyl-L-Proline; H02: p-Hydroxy Phenyl Acetic Acid; H03: m-Hydroxy Phenyl Acetic Acid; H04: Tyramine; H05: D-Psicose; H06: L-Lyxose; H07: Glucuronamide; H08: Pyruvic Acid; H09: L-Galactonic Acid-γ-Lactone; H10: D-Galacturonic Acid; H11: Phenylethylamine; H12: 2-Aminoethanol. PM02A: A01: Negative Control; A02: Chondroitin Sulfate C; A03: α-Cyclodextrin; A04: β-Cyclodextrin; A05: γ-Cyclodextrin; A06: Dextrin; A07: Gelatin; A08: Glycogen; A09: Inulin; A10: Laminarin; A11: Mannan; A12: Pectin; B01: N-Acetyl-D-Galactosamine; B02: N-Acetyl-Neuraminic Acid; B03: β-D-Allose; B04: Amygdalin; B05: D-Arabinose; B06: D-Arabitol; B07: L-Arabitol; B08: Arbutin; B09: 2-Deoxy-D-Ribose; B10: I-Erythritol; B11: D-Fuctose; B12: 3-0-β-D-Galactopyranosyl-D-Arabinose; C01: Gentiobiose; C02: L-Glucose; C03: Lactitol; C04: D-Melezitose; C05: Maltitol; C06: α-Methyl-D-Glucoside; C07: β-Methyl-D-Galactoside; C08: 3-Methyl-Glucose; C09: β-Methyl-D-Glucuronic Acid; C10: α-Methyl-D-Mannoside; C11: β-Methyl-D-Xyloside; C12: Palatinose; D01: D-Raffinose; D02: Salicin; D03: Sedoheptulosan; D04: L-Sorbose; D05: Stachyose; D06: D-Tagatose; D07: Turanose; D08: Xylitol; D09: N-Acetyl-D-Glucosaminitol; D10: γ-Amino Butyric Acid; D11: δ-Amino Valeric Acid; D12: Butyric Acid; E01: Capric Acid; E02: Caproic Acid; E03: Citraconic Acid; E04: Citramalic Acid; E05: D-Glucosamine; E06: 2-Hydroxy Benzoic Acid; E07: 4-Hydroxy Benzoic Acid; E08: β-Hydroxy Butyric Acid; E09: γ-Hydroxy Butyric Acid; E10: α-Keto Valeric Acid; E11: Itaconic Acid; E12: 5-Keto-D-Gluconic Acid; F01: D-Lactic Acid Methyl Ester; F02: Malonic Acid; F03: Melibionic Acid; F04: Oxalic Acid; F05: Oxalomalic Acid; F06: Quinic Acid; F07: D-Ribono-1,4-Lactone; F08: Sebacic Acid; F09: Sorbic Acid; F10: Succinamic Acid; F11: D-Tartaric Acid; F12: L-Tartaric Acid; G01: Acetamide; G02: L-Alaninamide; G03: N-Acetyl-L-Glutamic Acid; G04: L-Arginine; G05: Glycine; G06: L-Histidine; G07: L-Homoserine; G08: Hydroxy-L-Proline; G09: L-Isoleucine; G10: L-Leucine; G11: L-Lysine; G12: L-Methionine; H01: L-Ornithine; H02: L-Phenylalanine; H03: L-Pyroglutamic Acid; H04: L-Valine; H05: D,L-Carnitine; H06: Sec-Butylamine; H07: D,L-Octopamine; H08: Putrescine; H09: Dihydroxy Acetone; H10: 2,3-Butanediol; H11: 2,3-Butanone; H12: 3-Hydroxy-2-Butanone.
(TIF)

Figure S10 Dichotomous key used to differentiate species, subspecies and biovars of the *P. fluorescens* group. The ten strains of this study (Table 1) were evaluated for all phenotypes shown and classified as shown in Table S16 according to this key. Abbreviations and definitions are as follows: Fluorescence (fluorescence of colonies viewed under UV light); Arginine (arginine dihydrolase activity); Oxidase (oxidase activity); HR on Tobacco (hypersensitivity response on tobacco); Levan (levan sucrase activity); Gelatin (gelatinase activity); L-ara (L-arabinose); L-trp (L-tryptophan); *P. fluor.* (*P. fluorescens*); *P. chloro*. subsp. *aureo.* (*P. chlororaphis subsp. aureofaciens*); *P. chloro.* subsp. *chloro*. (*P. chlororaphis subsp. chlororaphis*); bv. (biovar). This scheme was revised from Bossis et al. [178] to focus on phenotypes exhibited by type strains (Table S16) that correlate to the phylogenies inferred in this study (Figure 1). Bold black boxes indicate phenotypes conferred by characterized loci that are present in strains exhibiting these traits (Table S16). Putative gene clusters corresponding to phenotypes shown in bold blue boxes have been identified in the genomes of this study (Table S16).
(TIF)

Table S1 Role category designations for genes within core genomes of *Pseudomonas* spp. Comparative BLASTp searches of the predicted proteomes of representative *Pseudomonas* spp. (shown in Figure 1) were used to identify the core genomes. Numbers show the percentages of genes within each role category represented by the core genomes of: (A) the *P. fluorescens* group (2789 genes); (B) *Pseudomonas* spp. excepting *P. stutzeri* and *P. mendocina* (1854 genes); and (C) *Pseudomonas* spp. (1491 genes). The role category designations are for a representative genome (Pf-5) as listed at the J. Craig Venter Institute Comprehensive Microbial Resource (http://cmr.jcvi.org/cgi-bin/CMR/shared/RoleList.cgi).
(PDF)

Table S2 Genes shared by and unique to ten strains within the *P. fluorescens* group. Locus tags represent CDSs conserved within the genomes of ten sequenced strains within the *P. fluorescens* group, but absent from the genomes of all other representative *Pseudomonas* spp.
(PDF)

Table S3 The proportion of CDSs shared among ten genomes in the *P. fluorescens* group. The proportions shown were calculated as the number of CDSs shared between each pair of strains divided by the number of CDSs in the strain with the smallest genome of the pair (i.e., the number of CDSs that could theoretically be shared by that pair of strains). Pink, blue and green shading highlights comparisons between pairs of strains within Sub-clades 1, 2 and 3, respectively.
(PDF)

Table S4 The number of CDSs shared among ten genomes in the *P. fluorescens* group. Pairwise numbers of CDSs shared between each pair of strains was determined using comparative BLASTp searches. Pink, blue and green shading highlight comparisons between pairs of strains within Sub-clades 1, 2 and 3, respectively.
(PDF)

Table S5 Genes shared by and unique to strains in Sub-clade 1 of the *P. fluorescens* group. Locus tags represent CDSs conserved among the genomes of strains Pf-5, 30-84, and O6, but absent from the genomes of all other representative *Pseudomonas* spp. These CDSs were identified from comparative BLASTp searches of the predicted proteomes of representative *Pseudomonas* spp. (shown in Figure 1).
(PDF)

Table S6 Genes shared by and unique to two strains of *P. chlororaphis*. Locus tags represent CDSs conserved among the genomes of *P. chlororaphis* strains 30-84, and O6, but absent from the genomes of all other representative *Pseudomonas* spp. These CDSs were identified from comparative BLASTp searches of the predicted proteomes of representative *Pseudomonas* spp. (shown in Figure 1).
(PDF)

Table S7 Genes shared by and unique to strains in Sub-clade 2 of the *P. fluorescens* group. Locus tags represent CDSs conserved among the genomes of strains Pf0-1, Q8r1-96, and Q2-87, but absent from the genomes of all other representative *Pseudomonas* spp. These CDSs were identified from comparative BLASTp searches of the predicted proteomes of representative *Pseudomonas* spp. (shown in Figure 1).
(PDF)

Table S8 Genes shared by and unique to strains Q8r1-96 and Q2-87. Locus tags represent CDSs conserved among the genomes of strains Q8r1-96, and Q2-87, but absent from the genomes of all other representative *Pseudomonas* spp. These CDSs were identified from comparative BLASTp searches of the predicted proteomes of representative *Pseudomonas* spp. (shown in Figure 1).
(PDF)

Table S9 Genes shared by and unique to strains in Sub-clade 3 of the *P. fluorescens* group. Locus tags represent CDSs conserved among the genomes of strains BG33R, SBW25, A506, and SS101, but absent from the genomes of all other representative *Pseudomonas* spp. These CDSs were identified from comparative BLASTp searches of the predicted proteomes of representative *Pseudomonas* spp. (shown in Figure 1).
(PDF)

Table S10 Consensus sequences and logos of REP elements in the genomes of the *P. fluorescens* group. HMM searches were used to identify the occurrence of REP elements within the genomes of strains in the *P. fluorescens* group. The number of occurrences as well as the consensus sequence and consensus sequence logo are presented for REP elements appearing more than 250 times in a genome. Imperfect palindromes identified within the consensus sequence are highlighted in red and blue and palindromic nucleotides are underlined.
(PDF)

Table S11 REP HMM hits across *Pseudomonas* spp. genome sequences. HMM searches across the genomes of a collection of *Pseudomonas* strains were conducted using the REP sequences identified within the *P. fluorescens* group to gauge the broader distribution of these sequence elements. Shading highlights strains containing large numbers of REP sequence elements: REPa, grey; REPb, pink; REPc, green; REPd, orange; REPe, blue.
(PDF)

Table S12 Transposons present in the genomes of seven strains in the *P. fluorescens* group. The following information is provided for each putative transposon in the genomes of strains 30-84, O6, Q8r1-96, Q2-87, BG33R, A506, and SS101: transposon family, transposases, and numbers of copies of intact or remnant transposons in each genome.
(PDF)

Table S13 Mobile genetic elements in the genomes of seven strains in the *P. fluorescens* group. The following information is provided for each prophage or genomic island in the genomes of strains 30-84, O6, Q8r1-96, Q2-87, BG33R, A506, and SS101: presence of an integrase, insertion site, size, locus tags, and selected cargo genes.
(PDF)

Table S14 Bioassays linking gene inventories to phenotypes of strains in the *P. fluorescens* group. Ten strains were evaluated for the production of levan sucrase, exoprotease, gelatinase, lipase, chitinase, and hydrogen cyanide as well as biosurfactant and hemolytic activities associated with cyclic lipopetide production. Derivatives of some strains having mutations in *ofaA*, *aprA*, *hcnB*, *viscA*, *massA*, or *gacA* were also evaluated to serve as negative controls in these experiments correlating genotypes to phenotypes. A derivative of Pf0-1 containing a plasmid-borne *gacA* ^+^ produced exoprotease, gelatinase, lipase, chitinase, and hydrogen cyanide and exhibited biosurfactant and hemolytic activity. In contrast, strain Pf0-1 was negative for these phenotypes, supporting our conclusion that the sequenced strain of Pf0-1 has a mutation in *gacA*.
(PDF)

Table S15 Putative type III secretion system effectors were identified in six genomes of the *P. fluorescens* group. T3SS effectors were identified by BLASTp, based on their similarities to members of known bacterial effector families. The six genomes also were screened using hidden Markov models (HMMs) built from the compilation of *P. syringae* Hrp boxes. Putative T3SS effector genes were identified in the Q8r1-96, A506, SS101 and BG33R genomes based on the presence of possible Hrp boxes and N-termini typical of T3SS-secreted proteins (i.e., abundance of Ser and polar residues at the N-termini, only one acidic residue in the first 12 positions, and an aliphatic amino acid in position 3 or 4). The following information is provided for each putative T3SS effector: Locus tag, gene name, %G+C, sequence of the putative Hrp box, amino acid residues in the N-terminus, and closest protein match.
(PDF)

Table S16 Phenotypes of strains in the *P. fluorescens* group and putative genes conferring these phenotypes. The ten strains listed in Table 1 were tested for phenotypes that have been used to classify strains into species of *Pseudomonas* or biovars of *P. fluorescens*. Results of the assays are listed as either positive (+), negative (−), or variable (v); controls for each assay are shown. Based on their phenotypes, we assigned the strains to biovars of *P. fluorescens* according to the scheme presented in Figure S10 or to established species within the *P. fluorescens* group [31], [130], [131]. Putative genes associated with the tested phenotypes were identified and listed.
(PDF)

Table S17 Primers used to construct *hcnB*, *aprA*, and *chiC* mutants in *P. protegens* Pf-5.
(PDF)
