# Supplementary figures and images for: Comparative Genomics of Plant-Associated Pseudomonas spp.: Insights into Diversity and Inheritance of Traits Involved in Multitrophic Interactions
Source: PLoS Genet. 2012 Jul 5;8(7):e1002784. doi: 10.1371/journal.pgen.1002784 (PMC3390384; doi:10.1371/journal.pgen.1002784)

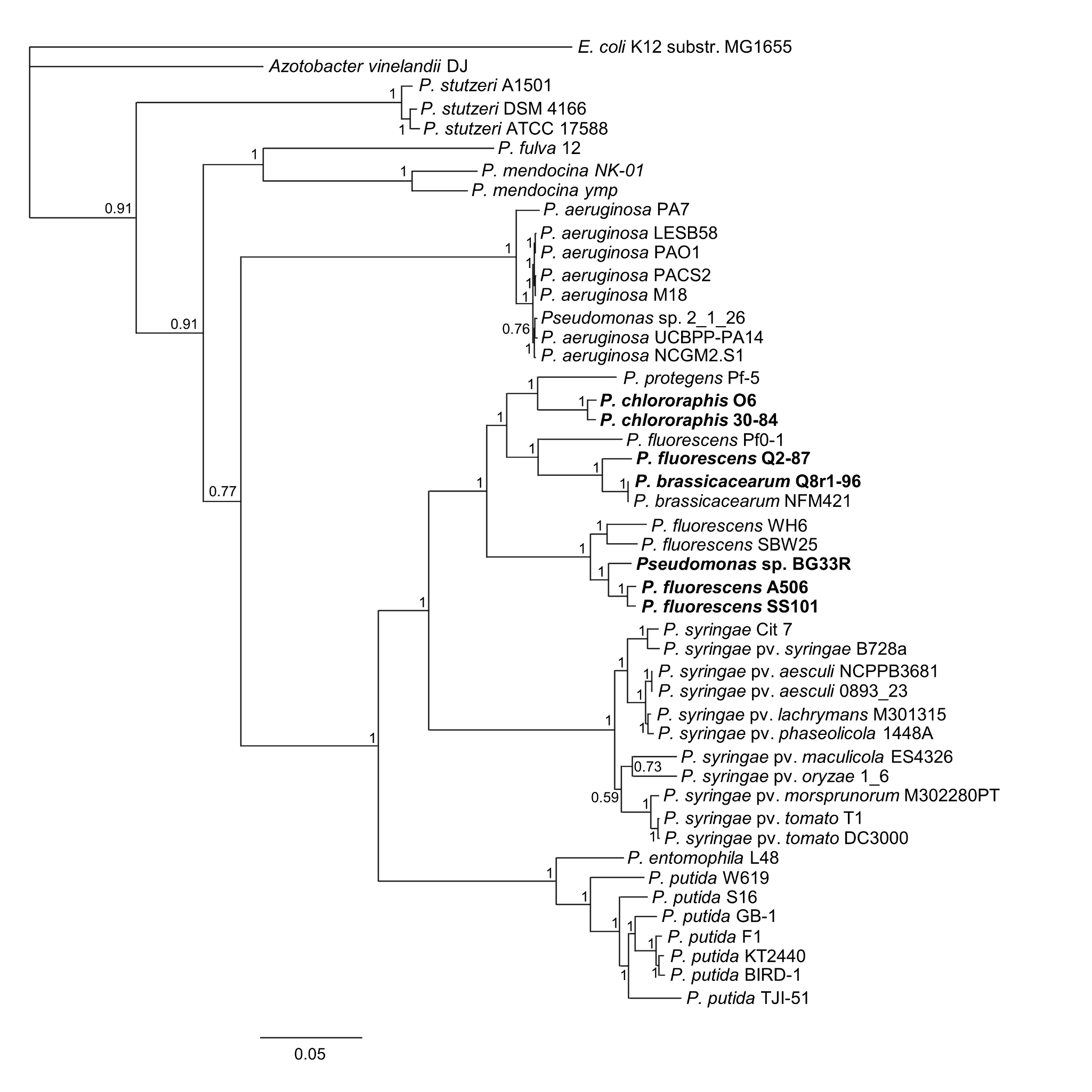

Supplement: Figure S2 — Phylogenetic tree depicting the relationships among sequenced strains of Pseudomonas spp. This maximum likelihood tree is based on the concatenated alignments of 726 shared proteins found within all of the genomes and was generated using the Hal pipeline [153]. The interior node values of the tree are representative of the number of bootstraps out of 100. (TIF) [file pgen.1002784.s002.tif]

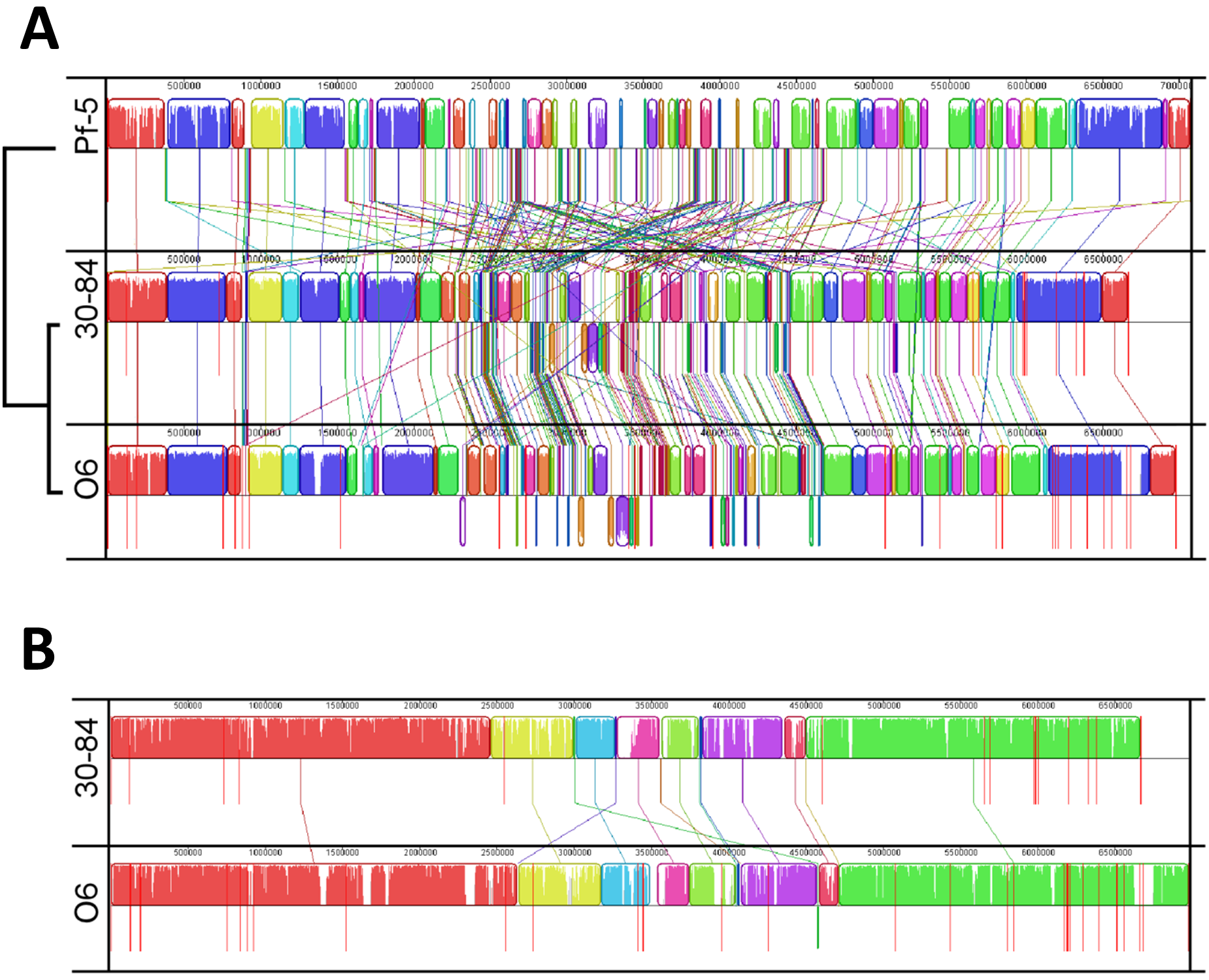

Supplement: Figure S3 — Chromosomal alignments of strains within Sub-clade 1 generated using Progressive MAUVE [151]. (A) P. protegens Pf-5, P. chlororaphis 30-84 and P. chlororaphis O6, (B) the P. chlororaphis strains only. Regions of significant synteny between the strains are shown as colored blocks in the mauve alignment. Regions of sequence not shared between the strains are seen as white gaps within the blocks or spaces between the blocks. Colored lines connect syntenous blocks of sequence between the strains. Breaks between scaffolds are designated by vertical red lines extending through and below the blocks of a genome (30-84 and O6). The tree on the left hand side of (A) shows the relatedness of the strains as determined by MSLA (Figure 1). (TIF) [file pgen.1002784.s003.tif]

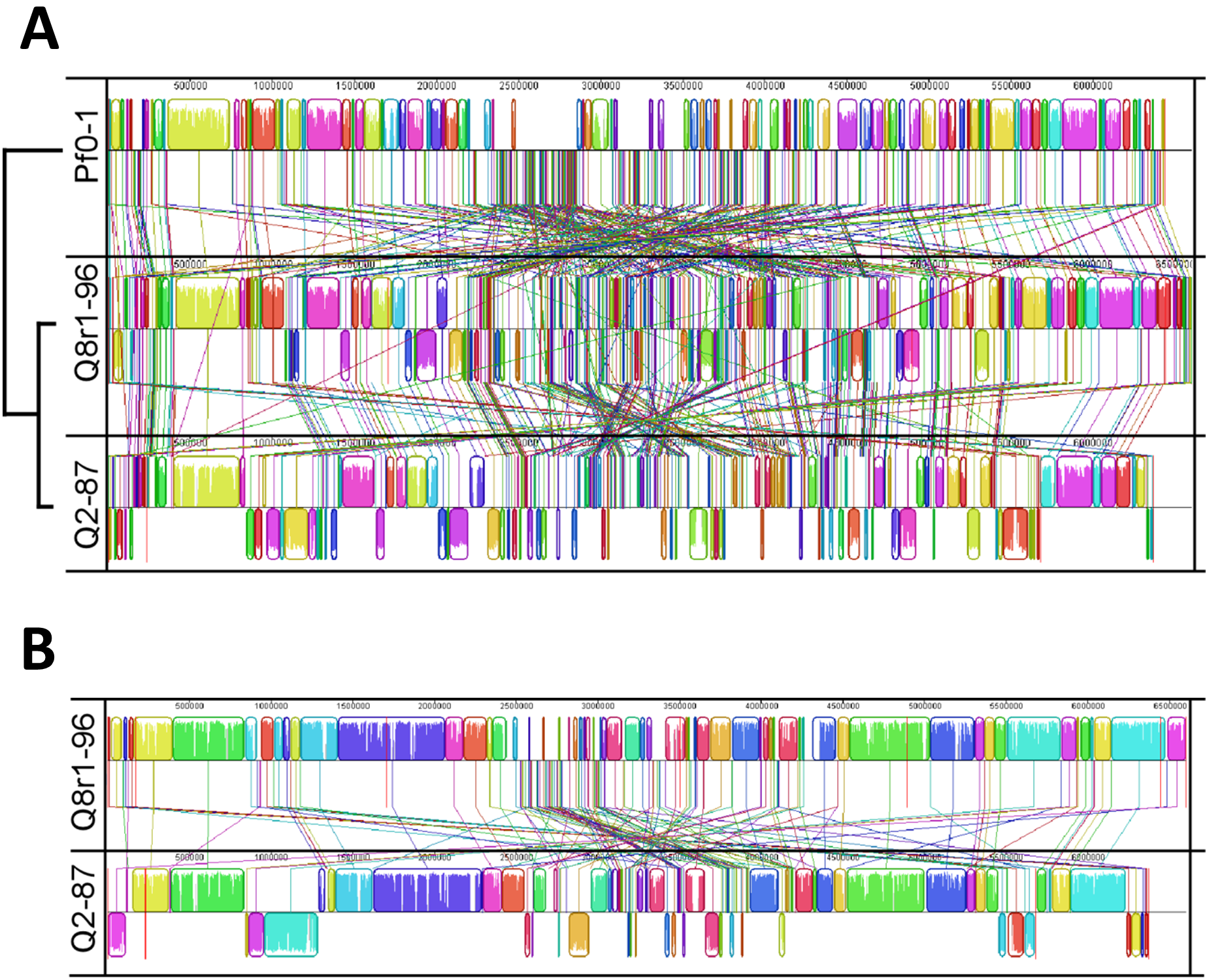

Supplement: Figure S4 — Chromosomal alignments of strains within Sub-clade 2 generated using Progressive MAUVE [151]. (A) P. fluorescens Pf0-1, P. fluorescens Q2-87, and P. brassicacearum Q8r1-96 and (B) P. brassicacearum Q8r1-96 and P. fluorescens Q2-87 only. Regions of significant synteny between the strains are shown as colored blocks in the mauve alignment. Regions of sequence not shared between the strains are seen as white gaps within the blocks or spaces between the blocks. Breaks between scaffolds are designated by vertical red lines extending through and below the blocks of each genome. Colored lines connect syntenous blocks of sequence between the strains. The tree on the left hand side of (A) shows the relatedness of the strains as determined by MSLA (Figure 1). (TIF) [file pgen.1002784.s004.tif]

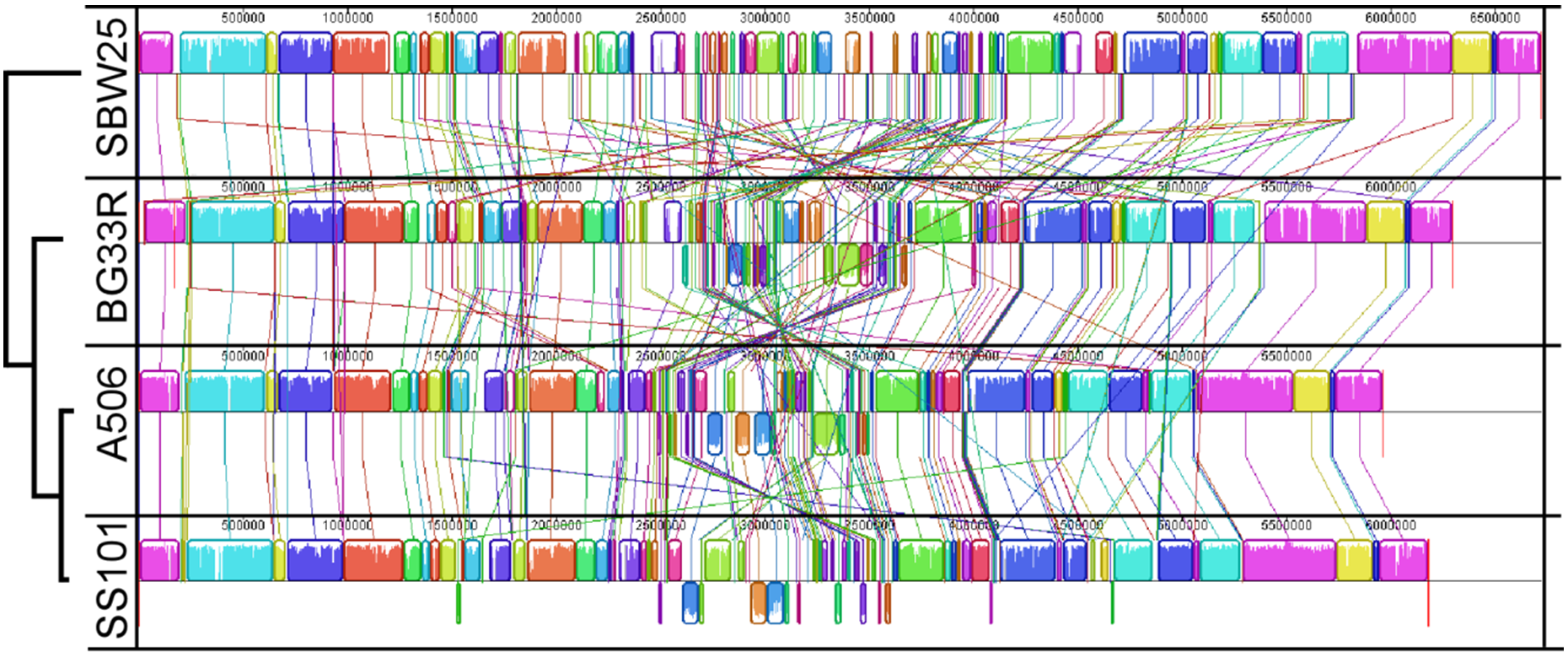

Supplement: Figure S5 — Chromosomal alignments of strains within Sub-clade 3 generated using Progressive MAUVE [151]. Regions of significant synteny between the strains (P. fluorescens SBW25, Pseudomonas sp. BG33R, P. fluorescens A506 and P. fluorescens SS101) are shown as colored blocks in the mauve alignment. Regions of sequence not shared between the strains are seen as white gaps within the blocks or spaces between the blocks. Breaks between scaffolds are designated by vertical red lines extending through and below the blocks of genome BG33R. Colored lines connect syntenous blocks of sequence between the strains. The tree on the left hand side of the figure shows the relatedness of the strains as determined by MSLA (Figure 1). (TIF) [file pgen.1002784.s005.tif]

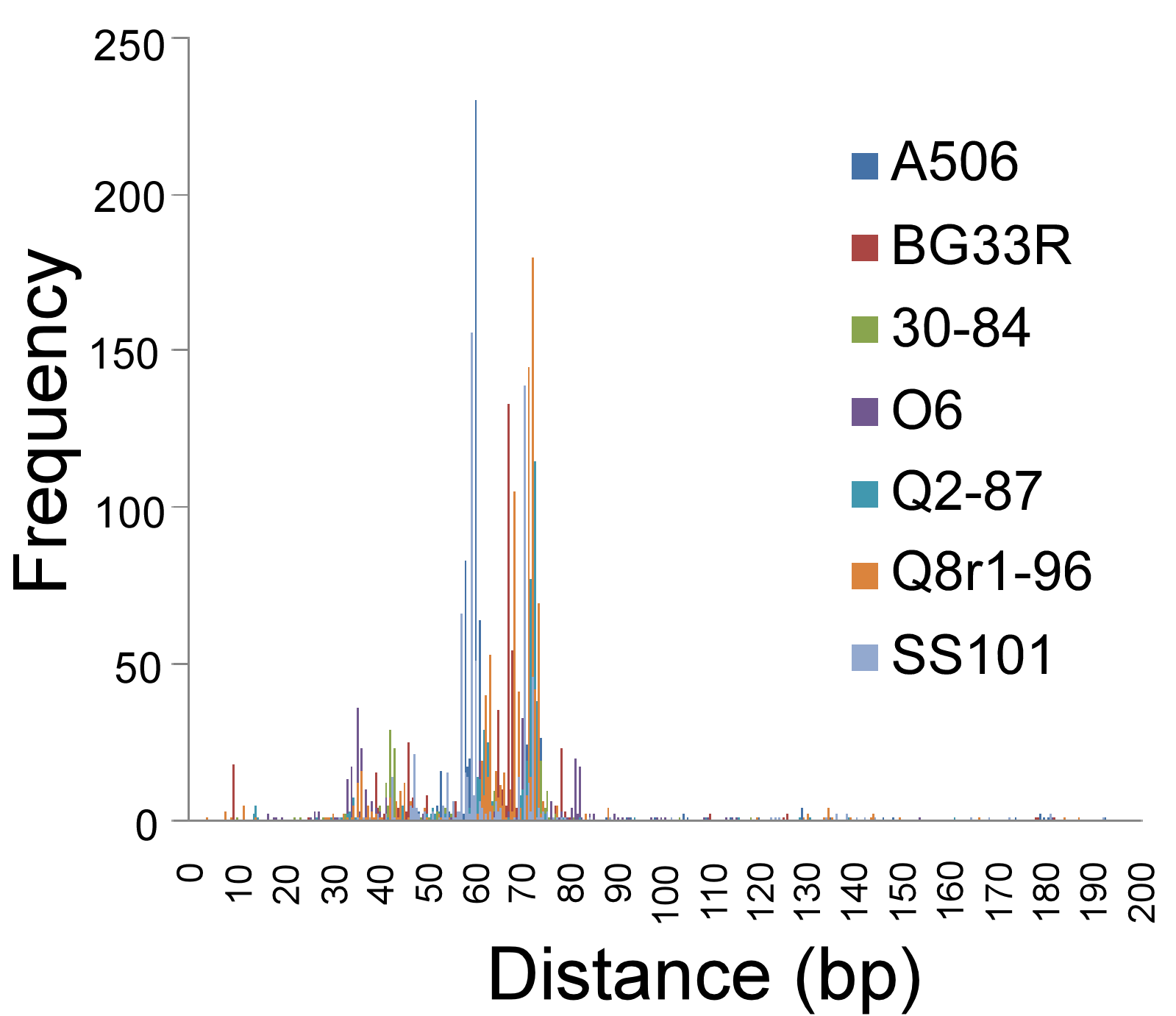

Supplement: Figure S6 — REP frequency. Local spacing of REPa sequence elements. The frequency of the distance (bp) between adjacent REPa sequences separated by fewer than 200 bp is shown for each of the seven newly-sequenced strains. Distances were measured from the center of REPa sequences. (TIF) [file pgen.1002784.s006.tif]

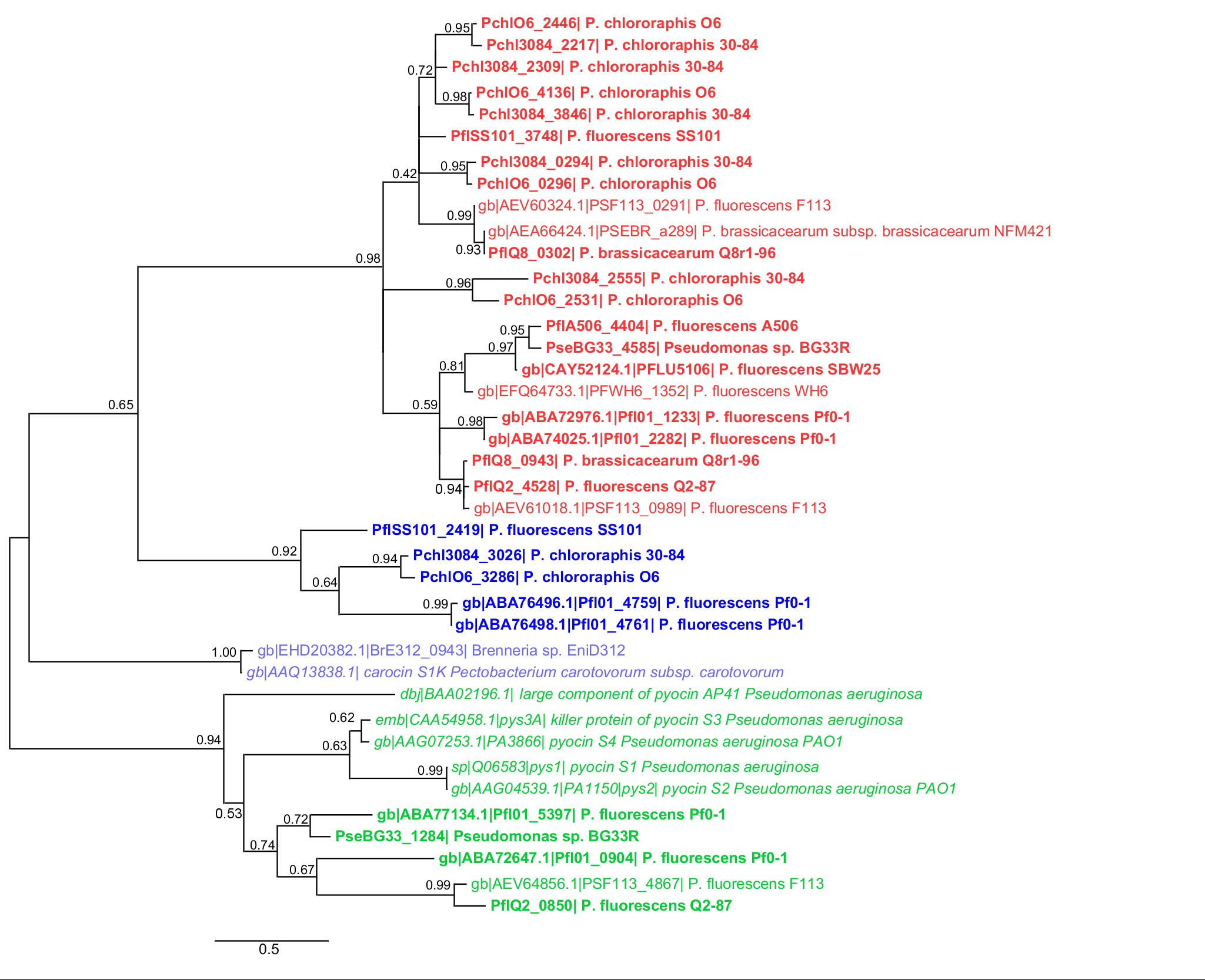

Supplement: Figure S8 — Phylogenetic tree depicting the relationships of pyocin-like bacteriocins found in genomes of the P. fluorescens group. Translocation domains (Pfam: PF06958) were used for this analysis; they are the most conserved domain in the pyocin-like proteins. Proteins found within the ten genomes examined in this study are bolded; characterized proteins are italicized. Interior node values of the tree are representative of the number of bootstraps out of 1000. Color coding is as follows: bacteriocin group N1 (Red), group N2 (Dark blue), carocin (Light blue), Pyocin S1/2/AP41-like (Green). (TIF) [file pgen.1002784.s008.tif]

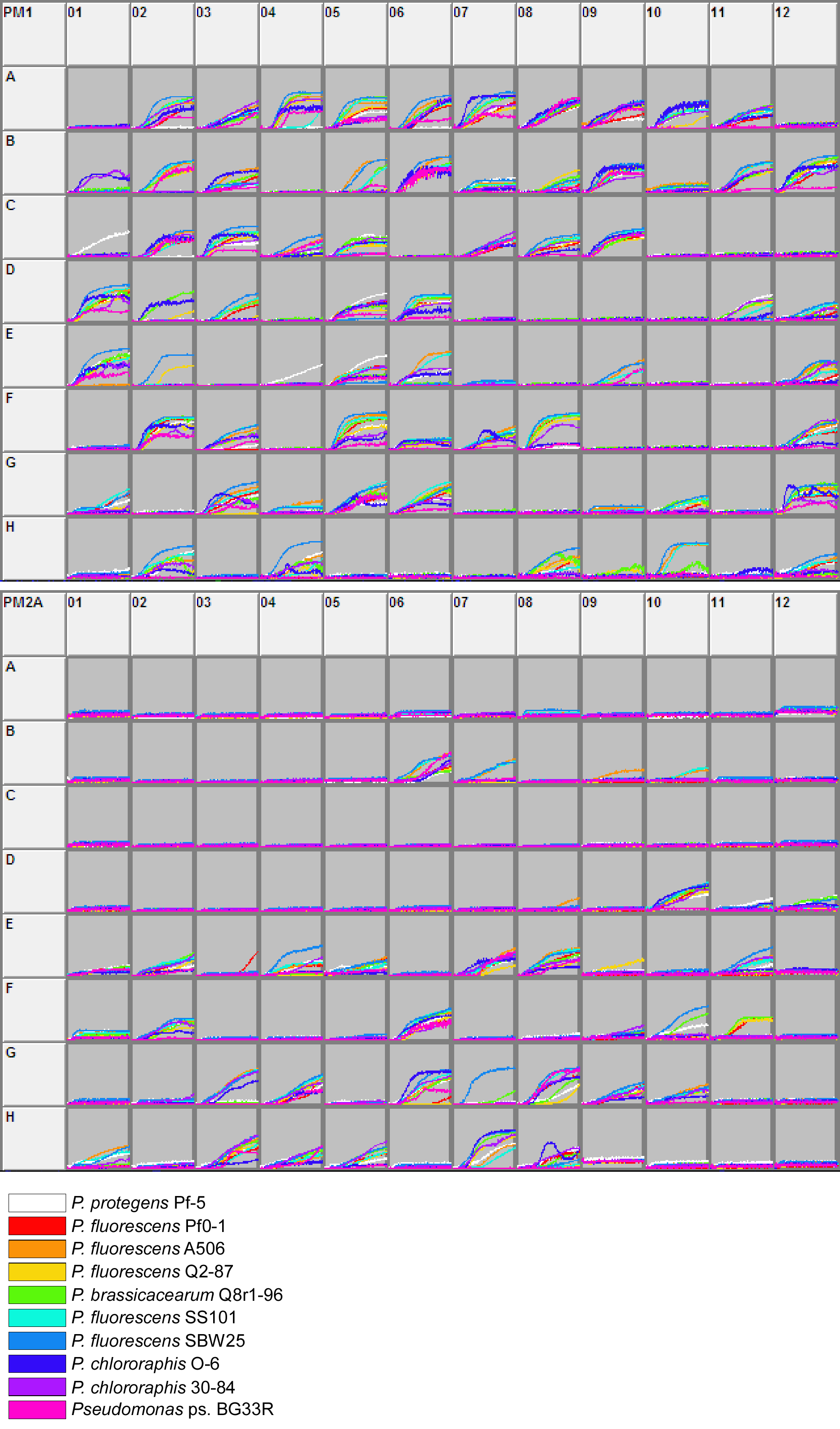

Supplement: Figure S9 — Kinetic curves depicting rates of respiration of strains in the P. fluorescens group grown in Biolog PM carbon utilization plates PM01 and PM02. Substrates are as follows: PM01: A01: Negative Control; A02: L-Arabinose; A03: N-Acetyl-D-Glucosamine; A04: D-Saccharic Acid; A05: Succinic Acid; A06: D-Galactose; A07: L-Aspartic Acid; A08: L-Proline; A09: D-Alanine; A10: D-Trehalose; A11: D-Mannose; A12: Dulcitol; B01: D-Serine; B02: D-Sorbitol; B03: Glycerol; B04: L-Fucose; B05: D-Glucuronic Acid; B06: D-Gluconic Acid; B07: D,L-α-Glycerol-Phosphate; B08: D-Xylose; B09: L-Lactic Acid; B10: Formic Acid; B11: D-Mannitol; B12: L-Glutamic Acid; C01: D-Glucose-6-Phosphate; C02: D-Galactnoic Acid-γ-Lactone; C03: D,L-Malic Acid; C04: D-Ribose; C05: Tween 20; C06: L-Rhamnose; C07: D-Fructose; C08: Acetic Acid; C09: α-D-Glucose; C10: Maltose; C11: D-Melibiose; C12: Thymidine; D01: L-Asparagine; D02: D-Aspartic Acid; D03: D-Glucosaminic Acid; D04: 1,2-Propanediol; D05: Tween 40; D06: α-Keto-Glutaric Acid; D07: α-Keto-Butyric Acid; D08: α-Methyl-D-Galactoside; D09: α-D-Lactose; D10: Lactulose; D11: Sucrose; D12: Uridine; E01: L-Glutamine; E02: M-Tartaric Acid; E03: D-Glucose-1-Phosphate; E04: D-Fructose-6-Phosphate; E05: Tween 80; E06: α-Hydroxy Glutaric Acid-γ-Lactone; E07: α-Hydroxy Butyric Acid; E08: β-Methyl-D-Glucoside; E09: Adonitol; E10: Maltotriose; E11: 2-Deoxy Adenosine; E12: Adenosine; F01: Glycyl-L-Aspartic Acid; F02: Citric Acid; F03: M-Inositol; F04: D-Threonine; F05: Fumaric Acid; F06: Bromo Succinic Acid; F07: Propionic Acid; F08: Mucic Acid; F09: Glycolic Acid; F10: Glyoxylic Acid; F11: D-Cellobiose; F12: Inosine; G01: Glycyl-L-Glutamic Acid; G02: Tricarballylic Acid; G03: L-Serine; G04: L-Threonine; G05: L-Alanine; G06: L-Alanyl-Glycine; G07: Acetoacetic Acid; G08: N-Acetyl-β-D-Mannosamine; G09: Mono Methyl Succinate; G10: Methyl Pyruvate; G11: D-Malic Acid; G12: L-Malic Acid; H01: Glycyl-L-Proline; H02: p-Hydroxy Phenyl Acetic Acid; H03: m-Hydroxy Phenyl Acetic Ac [file pgen.1002784.s009.tif]

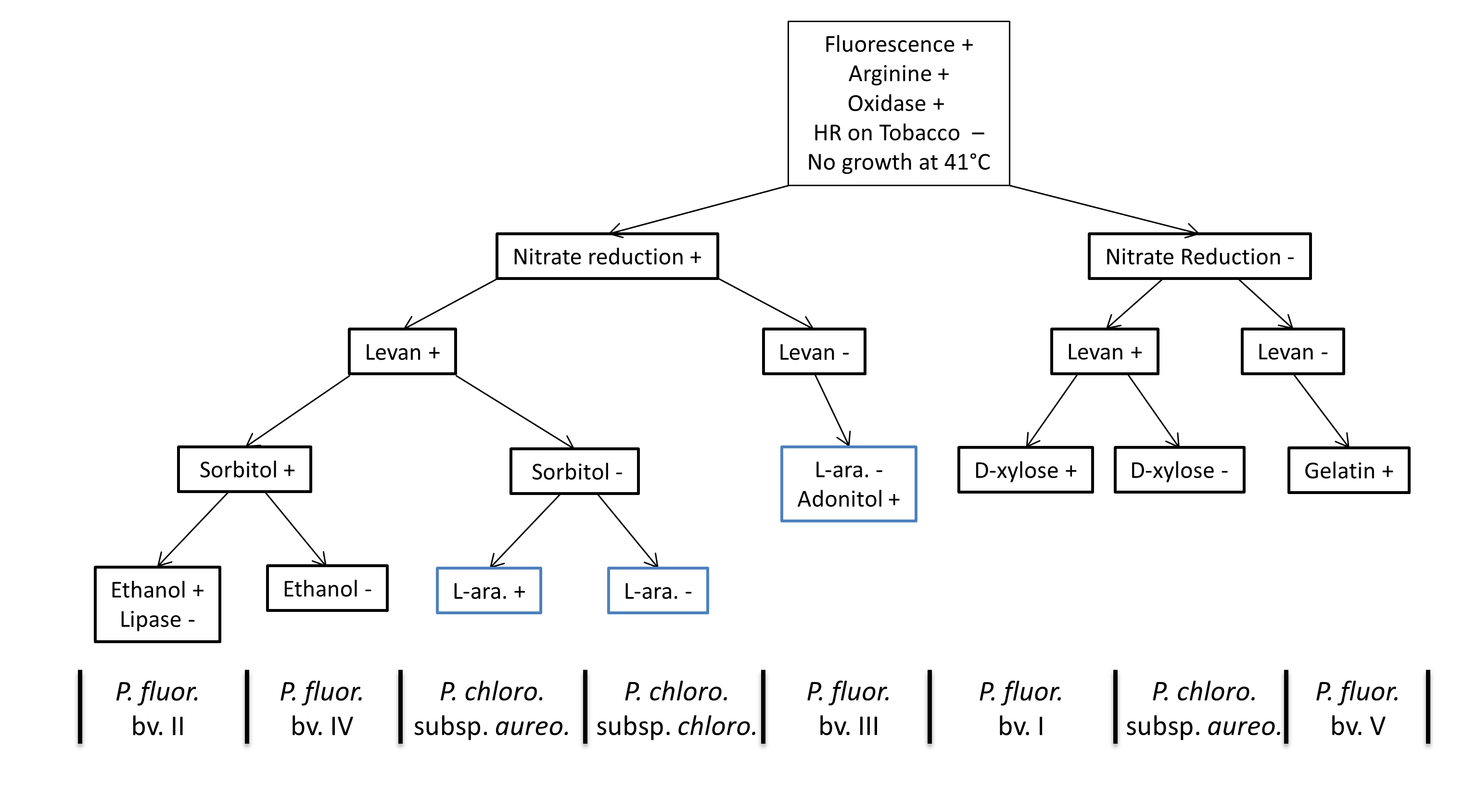

Supplement: Figure S10 — Dichotomous key used to differentiate species, subspecies and biovars of the P. fluorescens group. The ten strains of this study (Table 1) were evaluated for all phenotypes shown and classified as shown in Table S16 according to this key. Abbreviations and definitions are as follows: Fluorescence (fluorescence of colonies viewed under UV light); Arginine (arginine dihydrolase activity); Oxidase (oxidase activity); HR on Tobacco (hypersensitivity response on tobacco); Levan (levan sucrase activity); Gelatin (gelatinase activity); L-ara (L-arabinose); L-trp (L-tryptophan); P. fluor. (P. fluorescens); P. chloro. subsp. aureo. (P. chlororaphis subsp. aureofaciens); P. chloro. subsp. chloro. (P. chlororaphis subsp. chlororaphis); bv. (biovar). This scheme was revised from Bossis et al. [178] to focus on phenotypes exhibited by type strains (Table S16) that correlate to the phylogenies inferred in this study (Figure 1). Bold black boxes indicate phenotypes conferred by characterized loci that are present in strains exhibiting these traits (Table S16). Putative gene clusters corresponding to phenotypes shown in bold blue boxes have been identified in the genomes of this study (Table S16). (TIF) [file pgen.1002784.s010.tif]
