# Supplementary material for: Comparative Genomics of Plant-Associated Pseudomonas spp.: Insights into Diversity and Inheritance of Traits Involved in Multitrophic Interactions
Source: PLoS Genet. 2012 Jul 5;8(7):e1002784. doi: 10.1371/journal.pgen.1002784 (PMC3390384; doi:10.1371/journal.pgen.1002784)
Supplement: Figure S7 — Similarities between cargo genes in different mobile genetic elements present in genomes of the P. fluorescens group. The level of similarity is depicted by the strength of grey shading in boxes representing overlap between two elements. Abbreviations: pro (prophage); isl (island); plas (plasmid); tn (transposon). +ni = integrase present and not intact; + = integrase present and intact; − = integrase not present. Data for Pf-5 was published previously [54]; SBW25 and Pf0-1 were not examined in this analysis. (PDF) [file pgen.1002784.s007.pdf]

Strain	Element (Gene block)	Integrase
30-84	prophage 1 (I)	-
30-84	prophage 1 (II)	-
30-84	prophage 2	+[ni]
30-84	prophage 3	+[ni]
30-84	island 1	+
30-84	island 2	+
30-84	island 3	+
O6	prophage 1 (I)	-
O6	prophage 1 (II)	-
O6	prophage 1 (IV)	+
O6	prophage 2	+
O6	prophage 3	+
O6	island 1	+
O6	island 2	+
O6	island 3	+
A506	prophage 1 (I)	-
A506	prophage 2	+
A506	prophage 3	+
A506	island 1	+
A506	island 2	+
A506	island 3	+
A506	plasmid	+
Q8r1-96	prophage 1 (II)	-
Q8r1-96	prophage 2	+
Q8r1-96	island 1	+
Q8r1-96	island 2	+[ni]
Q8r1-96	island 3	+
Q8r1-96	island 4	+
Q8r1-96	transposon 1	-
Q2-87	prophage 1 (I)	-
Q2-87	prophage 2	+
Q2-87	island 1	+
Q2-87	island 2	+
Q2-87	island 3	+
Q2-87	island 4	+[ni]
Q2-87	island 5	+
SS101	prophage 1 (I)	-
SS101	island 1	+
SS101	island 2	+
SS101	island 3	+
SS101	island 4	+
SS101	island 5	+
SS101	island 6	+
SS101	island 7	+
BG33R	prophage 1 (I)	-
BG33R	prophage 1 (III)	-
BG33R	prophage 1 (V)	+
BG33R	prophage 2	+
BG33R	prophage 3	+
BG33R	prophage 4	+
BG33R	island 1	+
BG33R	island 2	+
BG33R	island 3	+
BG33R	island 4	+
BG33R	island 5	+[ni]
BG33R	island 6	+
BG33R	island 7	+
Pf-5	prophage 1 (II)	
Pf-5	prophage 2	
Pf-5	prophage 3	
Pf-5	prophage 4	
Pf-5	prophage 5	
Pf-5	prophage 6	
Pf-5	PFGI-1	
Pf-5	PFGI-2	

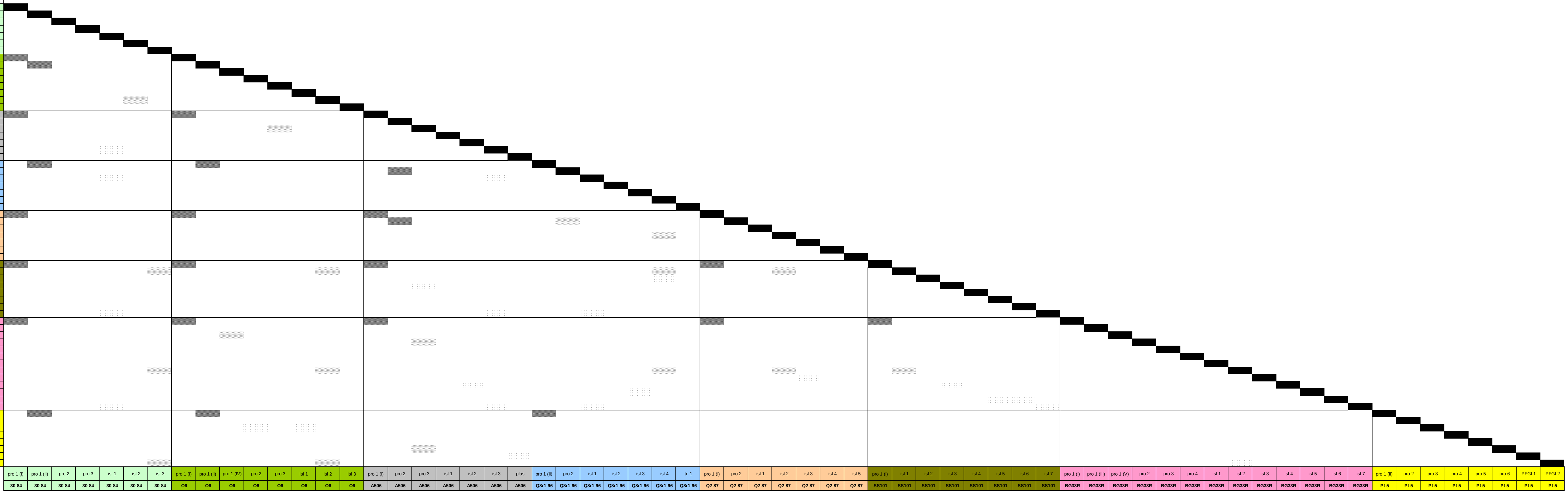

Strong similarity over extended range
Medium level of similarity
Weak similarity over a few genes
No similarity
