# Supplementary material for: Comparative Genomics of Plant-Associated Pseudomonas spp.: Insights into Diversity and Inheritance of Traits Involved in Multitrophic Interactions
Source: PLoS Genet. 2012 Jul 5;8(7):e1002784. doi: 10.1371/journal.pgen.1002784 (PMC3390384; doi:10.1371/journal.pgen.1002784)
Supplement: Table S1 — Role category designations for genes within core genomes of Pseudomonas spp. Comparative BLASTp searches of the predicted proteomes of representative Pseudomonas spp. (shown in Figure 1) were used to identify the core genomes. Numbers show the percentages of genes within each role category represented by the core genomes of: (A) the P. fluorescens group (2789 genes); (B) Pseudomonas spp. excepting P. stutzeri and P. mendocina (1854 genes); and (C) Pseudomonas spp. (1491 genes). The role category designations are for a representative genome (Pf-5) as listed at the J. Craig Venter Institute Comprehensive Microbial Resource (http://cmr.jcvi.org/cgi-bin/CMR/shared/RoleList.cgi). (PDF) [file pgen.1002784.s011.pdf]

Table S1. Role category designations for genes within the core genome

**Role category**	**% Total[a]**		
	**A**	**B**	**C**
Amino acid biosynthesis	65.47	53.96	51.08
Biosynthesis of cofactors, prosthetic groups, and carriers	69.78	60.99	55.49
Cell envelope	43.89	32.13	25.11
Cellular processes	52.10	35.29	26.89
Central intermediary metabolism	46.34	32.93	26.83
DNA metabolism	78.64	66.02	61.17
Energy metabolism	58.02	38.40	27.43
Fatty acid and phospholipid metabolism	52.60	29.48	24.28
Hypothetical proteins	28.92	15.60	12.48
Mobile and extrachromosomal element functions	3.57	0.00	0.00
Protein fate	60.59	47.78	44.33
Protein synthesis	84.11	70.20	69.54
Purines, pyrimidines, nucleosides, and nucleotides	90.32	80.65	75.81
Regulatory functions	42.50	23.96	17.47
Signal transduction	47.67	27.91	23.26
Transcription	61.45	49.40	48.19
Transport and binding proteins	41.65	25.18	14.74
Unknown function	44.22	28.40	23.23
Metabolism	58.29	39.42	30.89

[a] Numbers are percentages of the total number of genes with each role category designation within a representative genome (*Pseudomonas protegens* Pf-5). A: the 2789 core genes of the *P. fluorescens* group; B: 1854 core genes of the *Pseudomonas* (except *P. stutzeri* and *P. mendocina*); C: 1491 core genes of sequenced *Pseudomonas* spp. (see Figure 1).

doi: 10.1371/journal.pgen.1002784
