# Supplementary material for: Comparative Genomics of Plant-Associated Pseudomonas spp.: Insights into Diversity and Inheritance of Traits Involved in Multitrophic Interactions
Source: PLoS Genet. 2012 Jul 5;8(7):e1002784. doi: 10.1371/journal.pgen.1002784 (PMC3390384; doi:10.1371/journal.pgen.1002784)
Supplement: Table S2 — Genes shared by and unique to ten strains within the P. fluorescens group. Locus tags represent CDSs conserved within the genomes of ten sequenced strains within the P. fluorescens group, but absent from the genomes of all other representative Pseudomonas spp. (PDF) [file pgen.1002784.s012.pdf]

Table S2. Genes shared by and unique to ten fully-sequenced strains in the *Pseudomonas fluorescens* group[a]

**Annotated function**	**Pf-5**	**30-84**	**O6**	**Pf0-1**	**Q8r1-96**	**Q2-87**	**BG33R**	**SBW25**	**A506**	**SS101**
Conserved hypothetical protein	PFL_0092	Pchl3084_0090	PchlO6_0094	Pfl01_0045	PflQ8_0052	PflQ2_0066	PseBG33_0108	PFLU0089	PflA506_0088	PflSS101_0088
Biofilm PGA synthesis protein PgaA	PFL_0161	Pchl3084_0182	PchlO6_0181	Pfl01_0177	PflQ8_0168	PflQ2_5565	PseBG33_0177	PFLU0143	PflA506_0154	PflSS101_0155
Biofilm PGA synthesis lipoprotein PgaB	PFL_0162	Pchl3084_0183	PchlO6_0182	Pfl01_0178	PflQ8_0169	PflQ2_5564	PseBG33_0178	PFLU0144	PflA506_0155	PflSS101_0156
Biofilm PGA synthesis N-glycosyltransferase PgaC	PFL_0163	Pchl3084_0184	PchlO6_0183	Pfl01_0179	PflQ8_0170	PflQ2_5563	PseBG33_0179	PFLU0145	PflA506_0156	PflSS101_0157
Putative biofilm PGA synthesis protein PgaD	PFL_0164	Pchl3084_0185	PchlO6_0184	Pfl01_0180	PflQ8_0171	PflQ2_5562	PseBG33_0180	PFLU0146	PflA506_0157	PflSS101_0158
Conserved hypothetical protein	PFL_1293	Pchl3084_1294	PchlO6_1369	Pfl01_1241	PflQ8_1215	PflQ2_4257	PseBG33_1466	PFLU1339	PflA506_1296	PflSS101_4100
Conserved hypothetical protein	PFL_2043	Pchl3084_1970	PchlO6_2191	Pfl01_1872	PflQ8_3930	PflQ2_1714	PseBG33_3750	PFLU4227	PflA506_3549	PflSS101_3585
Transcriptional regulator, DeoR family	PFL_2429	Pchl3084_2237	PchlO6_2468	Pfl01_2141	PflQ8_2635	PflQ2_2551	PseBG33_3183	PFLU3612	PflA506_2356	PflSS101_3062
Response regulator	PFL_2454	Pchl3084_2272	PchlO6_2498	Pfl01_3529	PflQ8_2054	PflQ2_2059	PseBG33_4589	PFLU5118	PflA506_4409	PflSS101_4477
Putative cytochrome P450 oxidoreductase	PFL_2514	Pchl3084_2315	PchlO6_2537	Pfl01_3472	PflQ8_3443	PflQ2_3353	PseBG33_2095	PFLU3256	PflA506_2140	PflSS101_2021
Conserved hypothetical protein	PFL_2570	Pchl3084_2373	PchlO6_2593	Pfl01_3422	PflQ8_3419	PflQ2_2108	PseBG33_2088	PFLU2168	PflA506_2134	PflSS101_2014
DNA-binding response regulator	PFL_3269	Pchl3084_3225	PchlO6_3454	Pfl01_2892	PflQ8_3453	PflQ2_3362	PseBG33_3035	PFLU2722	PflA506_2968	PflSS101_2399
Transcriptional regulator, LysR family	PFL_3291	Pchl3084_2626	PchlO6_2861	Pfl01_2190	PflQ8_3335	PflQ2_2187	PseBG33_3106	PFLU2649	PflA506_2457	PflSS101_2240
GDP-mannose pyrophosphatase NudK / conserved domain protein	PFL_3331	Pchl3084_2574	PchlO6_2807	Pfl01_3567	PflQ8_5806	PflQ2_5681	PseBG33_3179	PFLU3608	PflA506_2359	PflSS101_3059
Conserved hypothetical protein	PFL_3866	Pchl3084_3665	PchlO6_3958	Pfl01_3573	PflQ8_3620	PflQ2_1921	PseBG33_3124	PFLU3541	PflA506_2422	PflSS101_2998
Conserved hypothetical protein	PFL_4007	Pchl3084_3794	PchlO6_4089	Pfl01_3715	PflQ8_3935	PflQ2_1712	PseBG33_2051	PFLU2131	PflA506_2096	PflSS101_1977
Transporter, CPA2 family	PFL_4229	Pchl3084_4091	PchlO6_4345	Pfl01_3967	PflQ8_1711	PflQ2_3768	PseBG33_1826	PFLU1748	PflA506_1790	PflSS101_1749
Aminotransferase, class V	PFL_4607	Pchl3084_4478	PchlO6_4719	Pfl01_4359	PflQ8_1258	PflQ2_4212	PseBG33_4327	PFLU4835	PflA506_4147	PflSS101_4223
Putative lipoprotein	PFL_5371	Pchl3084_5148	PchlO6_5415	Pfl01_4896	PflQ8_5035	PflQ2_4771	PseBG33_4824	PFLU5351	PflA506_4646	PflSS101_4700
Conserved hypothetical protein	PFL_6244	Pchl3084_2038	PchlO6_2252	Pfl01_1927	PflQ8_3792	PflQ2_1840	PseBG33_3664	PFLU4152	PflA506_3461	PflSS101_3478

[a] Genes are present in genomes of the *P. fluorescens* group strains, but are not present in the genomes of other *Pseudomonas* spp. in Figure 1.

doi: 10.1371/journal.pgen.1002784
