# Supplementary material for: Comparative Genomics of Plant-Associated Pseudomonas spp.: Insights into Diversity and Inheritance of Traits Involved in Multitrophic Interactions
Source: PLoS Genet. 2012 Jul 5;8(7):e1002784. doi: 10.1371/journal.pgen.1002784 (PMC3390384; doi:10.1371/journal.pgen.1002784)
Supplement: Table S3 — The proportion of CDSs shared among ten genomes in the P. fluorescens group. The proportions shown were calculated as the number of CDSs shared between each pair of strains divided by the number of CDSs in the strain with the smallest genome of the pair (i.e., the number of CDSs that could theoretically be shared by that pair of strains). Pink, blue and green shading highlights comparisons between pairs of strains within Sub-clades 1, 2 and 3, respectively. (PDF) [file pgen.1002784.s013.pdf]

Table S3. The proportion of CDSs shared among genomes in the *Pseudomonas fluorescens* group[a]

Strains	Pf-5	30-84	O6	Pf0-1	Q8r1-96	Q2-87	BG33R	SBW25	A506	SS101
**Pf-5**										
**30-84**	**0.74**									
**O6**	**0.72**	**0.90**								
**Pf0-1**	**0.70**	**0.72**	**0.73**							
**Q8r1-96**	**0.67**	**0.69**	**0.69**	**0.69**						
**Q2-87**	**0.66**	**0.69**	**0.70**	**0.71**	**0.83**					
**BG33R**	**0.69**	**0.70**	**0.71**	**0.68**	**0.64**	**0.64**				
**SBW25**	**0.67**	**0.67**	**0.67**	**0.68**	**0.68**	**0.68**	**0.77**			
**A506**	**0.72**	**0.72**	**0.72**	**0.70**	**0.68**	**0.67**	**0.84**	**0.78**		
**SS101**	**0.72**	**0.72**	**0.72**	**0.70**	**0.67**	**0.67**	**0.83**	**0.78**	**0.87**	

[a] CDSs shared between each pair of genomes in the *P. fluorescens* group as a proportion of the total number of CDSs in the strain with the smallest genome. Shading designates comparisons between strains within a specific sub-clade: pink, Sub-clade 1; blue, Sub-clade 2; green, Sub-clade 3.

doi: 10.1371/journal.pgen.1002784
