# Supplementary material for: Comparative Genomics of Plant-Associated Pseudomonas spp.: Insights into Diversity and Inheritance of Traits Involved in Multitrophic Interactions
Source: PLoS Genet. 2012 Jul 5;8(7):e1002784. doi: 10.1371/journal.pgen.1002784 (PMC3390384; doi:10.1371/journal.pgen.1002784)
Supplement: Table S4 — The number of CDSs shared among ten genomes in the P. fluorescens group. Pairwise numbers of CDSs shared between each pair of strains was determined using comparative BLASTp searches. Pink, blue and green shading highlight comparisons between pairs of strains within Sub-clades 1, 2 and 3, respectively. (PDF) [file pgen.1002784.s014.pdf]

Table S4. The number of CDSs shared among genomes in the *Pseudomonas fluorescens* group[a]

Strains	Pf-5	30-84	O6	Pf0-1	Q8r1-96	Q2-87	BG33R	SBW25	A506	SS101
**Pf-5**										
**30-84**	4310									
**O6**	4385	5233								
**Pf0-1**	3968	4120	4154							
**Q8r1-96**	3821	3904	3935	3948						
**Q2-87**	3710	3849	3891	3945	4641					
**BG33R**	3805	3845	3881	3761	3537	3535				
**SBW25**	3954	3898	3932	3858	3849	3783	4214			
**A506**	3811	3841	3845	3740	3589	3584	4441	4164		
**SS101**	3842	3879	3867	3751	3568	3563	4456	4202	4631	

[a] Shading designates the number of CDSs shared between two strains within a sub-clade: pink, Sub-clade 1; blue, Sub-clade 2; green, Sub-clade 3.

doi: 10.1371/journal.pgen.1002784
