# Supplementary material for: Comparative Genomics of Plant-Associated Pseudomonas spp.: Insights into Diversity and Inheritance of Traits Involved in Multitrophic Interactions
Source: PLoS Genet. 2012 Jul 5;8(7):e1002784. doi: 10.1371/journal.pgen.1002784 (PMC3390384; doi:10.1371/journal.pgen.1002784)
Supplement: Table S5 — Genes shared by and unique to strains in Sub-clade 1 of the P. fluorescens group. Locus tags represent CDSs conserved among the genomes of strains Pf-5, 30-84, and O6, but absent from the genomes of all other representative Pseudomonas spp. These CDSs were identified from comparative BLASTp searches of the predicted proteomes of representative Pseudomonas spp. (shown in Figure 1). (PDF) [file pgen.1002784.s015.pdf]

Table S5. Genes shared by and unique to Sub-clade 1[a]

Annotated function	Pf-5	30-84	O6
Diguanylate cyclase (GGDEF) domain protein	PFL_0087	Pchl3084_0085	PchlO6_0089
Polysaccharide deacetylase family protein	PFL_0288	Pchl3084_0297	PchlO6_0301
Conserved hypothetical protein	PFL_0319	Pchl3084_0334	PchlO6_0336
Putative acid phosphatase	PFL_0320	Pchl3084_0335	PchlO6_0337
MltA-interacting MipA family protein	PFL_0321	Pchl3084_0336	PchlO6_0338
Conserved hypothetical protein	PFL_0322	Pchl3084_0337	PchlO6_0339
Transcriptional regulator, AraC family	PFL_0323	Pchl3084_0338	PchlO6_0340
Transporter, major facilitator family	PFL_0380	Pchl3084_0375	PchlO6_0378
Putative lipoprotein	PFL_1007	Pchl3084_0997	PchlO6_1005
SEC-C domain protein	PFL_1271	Pchl3084_1271	PchlO6_1346
Conserved hypothetical protein	PFL_1388	Pchl3084_1736	PchlO6_1866
Acetyltransferase, GNAT family	PFL_1418	Pchl3084_1383	PchlO6_1458
Integral membrane protein, DUF6 family	PFL_1488	Pchl3084_1441	PchlO6_1565
Transcriptional regulator, AraC family	PFL_1558	Pchl3084_1506	PchlO6_1635
Conserved hypothetical protein	PFL_2179	Pchl3084_2308	PchlO6_2530
Conserved hypothetical protein	PFL_2227	Pchl3084_2078	PchlO6_2292
Succinylglutamate desuccinylase/aspartoacylase family protein	PFL_2253	Pchl3084_2109	PchlO6_2327
Putrescine ABC transporter, ATP-binding protein	PFL_2339	Pchl3084_2199	PchlO6_2422
Methyl-accepting chemotaxis protein	PFL_2407	Pchl3084_2223	PchlO6_2454
Peptidase, M48 family	PFL_2501	Pchl3084_2305	PchlO6_2526
Acetyltransferase, GNAT family	PFL_2567	Pchl3084_2370	PchlO6_2590
Conserved hypothetical protein	PFL_2569	Pchl3084_2372	PchlO6_2592
Conserved hypothetical protein	PFL_2685	Pchl3084_2535	PchlO6_2764
Conserved hypothetical protein	PFL_2773	Pchl3084_3318	PchlO6_3549
Conserved hypothetical protein	PFL_2782	Pchl3084_2532	PchlO6_2760
Transcriptional regulator, AraC family	PFL_2831	Pchl3084_2712	PchlO6_2942
Malate/L-lactate dehydrogenase family protein	PFL_2905	Pchl3084_2761	PchlO6_2993
Oxidoreductase membrane protein	PFL_2917	Pchl3084_2769	PchlO6_3001
Type I secretion system ATPase FitC	PFL_2982	Pchl3084_3528	PchlO6_3839
Cytotoxin FitD	PFL_2983	Pchl3084_3527	PchlO6_3838
Type I secretion outer membrane protein FitE	PFL_2984	Pchl3084_3526	PchlO6_3836
Sensory box sensor histidine kinase/response regulator FitF	PFL_2985	Pchl3084_3524	PchlO6_3834
Transcriptional regulator FitG	PFL_2986	Pchl3084_3525	PchlO6_3835
Response regulator FitH	PFL_2987	Pchl3084_3523	PchlO6_3833
DNA-binding heavy metal response regulator	PFL_3147	Pchl3084_2781	PchlO6_3013
Conserved hypothetical protein	PFL_3274	Pchl3084_2645	PchlO6_2880
Conserved hypothetical protein	PFL_3295	Pchl3084_2623	PchlO6_2858
Conserved hypothetical protein	PFL_3420	Pchl3084_3994	PchlO6_4259
Conserved hypothetical protein	PFL_3431	Pchl3084_3860	PchlO6_4150
Acetyltransferase, GNAT family	PFL_3459	Pchl3084_2978	PchlO6_3235
2-dehydropantoate 2-reductase	PFL_3476	Pchl3084_2494	PchlO6_2702
Transporter, major facilitator family	PFL_3477	Pchl3084_2493	PchlO6_2701
Conserved hypothetical protein	PFL_3600	Pchl3084_3539	PchlO6_3844
Tryptophan halogenase PrnA	PFL_3604	Pchl3084_3146	PchlO6_3706
Pyrrolnitrin biosynthesis enzyme PrnB	PFL_3605	Pchl3084_3145	PchlO6_3705

doi: 10.1371/journal.pgen.1002784

Aminopyrrolnitrin oxidase PrnD	PFL_3607	Pchl3084_3143	PchlO6_3703
Transcriptional regulator, AraC family	PFL_3820	Pchl3084_3057	PchlO6_3318
Conserved hypothetical protein	PFL_3840	Pchl3084_2361	PchlO6_2579
Conserved hypothetical protein	PFL_3841	Pchl3084_2360	PchlO6_2578
Conserved hypothetical protein	PFL_3845	Pchl3084_5245	PchlO6_5529
Conserved hypothetical protein	PFL_3929	Pchl3084_3728	PchlO6_4023
Putative cyclase	PFL_4035	Pchl3084_3821	PchlO6_4116
Putative membrane protein	PFL_4060	Pchl3084_3050	PchlO6_3311
Amidohydrolase family protein	PFL_4131	Pchl3084_3996	PchlO6_4261
Conserved hypothetical protein	PFL_4186	Pchl3084_4060	PchlO6_4314
Conserved hypothetical protein	PFL_4187	Pchl3084_4061	PchlO6_4315
Conserved hypothetical protein	PFL_4225	Pchl3084_4089	PchlO6_4343
Transcriptional regulator, TetR family	PFL_4557	Pchl3084_4438	PchlO6_4675
Translocator protein, LysE family	PFL_4582	Pchl3084_4454	PchlO6_4693
Putative membrane protein	PFL_4849	Pchl3084_4646	PchlO6_4896
Glycine hydroxymethyltransferase	PFL_5028	Pchl3084_4812	PchlO6_5065
Conserved hypothetical protein	PFL_5225	Pchl3084_4992	PchlO6_5261
Putative membrane protein	PFL_5390	Pchl3084_5158	PchlO6_5425
Conserved hypothetical protein	PFL_5499	Pchl3084_5248	PchlO6_5532
Conserved hypothetical protein	PFL_5709	Pchl3084_5444	PchlO6_5728
Conserved hypothetical protein	PFL_5757	Pchl3084_1965	PchlO6_2186
Aminotransferase, DegT/DnrJ/EryC1/StrS family	PFL_5960	Pchl3084_5674	PchlO6_5952
Conserved domain protein	PFL_6026	Pchl3084_5760	PchlO6_6118
Endoribonuclease L-PSP family protein	PFL_6037	Pchl3084_5771	PchlO6_6129
Conserved hypothetical protein	PFL_6130	Pchl3084_5867	PchlO6_6216
Conserved hypothetical protein	PFL_6143	Pchl3084_5874	PchlO6_6220
Threonine--tRNA ligase	PFL_6184	Pchl3084_5912	PchlO6_6260

[a] Genes are present in genomes of the strains found in Sub-clade 1, but are not present in the genomes of other *Pseudomonas* spp. in Figure 1.

doi: 10.1371/journal.pgen.1002784
