# Supplementary material for: Comparative Genomics of Plant-Associated Pseudomonas spp.: Insights into Diversity and Inheritance of Traits Involved in Multitrophic Interactions
Source: PLoS Genet. 2012 Jul 5;8(7):e1002784. doi: 10.1371/journal.pgen.1002784 (PMC3390384; doi:10.1371/journal.pgen.1002784)
Supplement: Table S6 — Genes shared by and unique to two strains of P. chlororaphis. Locus tags represent CDSs conserved among the genomes of P. chlororaphis strains 30-84, and O6, but absent from the genomes of all other representative Pseudomonas spp. These CDSs were identified from comparative BLASTp searches of the predicted proteomes of representative Pseudomonas spp. (shown in Figure 1). (PDF) [file pgen.1002784.s016.pdf]

Table S6. Genes shared by and unique to the *P. chlororaphis* strains[a]

**Annotated function**	**30-84**	**O6**
Hypothetical protein	Pchl3084_0006	PchlO6_0011
Aquaporin Z	Pchl3084_0007	PchlO6_0012
Cyclic dimeric GMP binding protein LapD	Pchl3084_0140	PchlO6_0137
Conserved hypothetical protein	Pchl3084_0190	PchlO6_0189
Hypothetical protein	Pchl3084_0191	PchlO6_0190
Conserved hypothetical protein	Pchl3084_0198	PchlO6_0199
Conserved hypothetical protein	Pchl3084_0229	PchlO6_0234
Membrane protein, putative	Pchl3084_0267	PchlO6_0273
Hypothetical protein	Pchl3084_0295	PchlO6_0298
Cytochrome c oxidase, subunit II, putative	Pchl3084_0325	PchlO6_0329
Cytochrome c oxidase, subunit I	Pchl3084_0326	PchlO6_0330
Cytochrome c oxidase, subunit III, putative	Pchl3084_0327	PchlO6_0331
Cytochrome c oxidase, subunit III, putative	Pchl3084_0328	PchlO6_0332
Conserved hypothetical protein	Pchl3084_0329	PchlO6_0333
Hypothetical protein	Pchl3084_0573	PchlO6_0579
Putative addiction module killer protein	Pchl3084_0634	PchlO6_0640
Conserved hypothetical protein	Pchl3084_0642	PchlO6_0648
Conserved hypothetical protein	Pchl3084_0678	PchlO6_0684
Acid phosphatase	Pchl3084_0708	PchlO6_0715
TonB-dependent outermembrane receptor	Pchl3084_0709	PchlO6_0716
Phosphorylase family protein	Pchl3084_0710	PchlO6_0717
Purine nucleoside permease, NUP family	Pchl3084_0711	PchlO6_0718
Transporter, MotA/TolQ/ExbB proton channel family	Pchl3084_0714	PchlO6_0721
Oxidoreductase, 2OG-Fe(II) oxygenase family	Pchl3084_0733	PchlO6_0738
AP endonuclease, family 2	Pchl3084_0734	PchlO6_0739
Conserved hypothetical protein	Pchl3084_0742	PchlO6_0747
Hypothetical protein	Pchl3084_0770	PchlO6_0772
Conserved hypothetical protein	Pchl3084_0786	PchlO6_0794
Conserved hypothetical protein	Pchl3084_0840	PchlO6_0846
Hypothetical protein	Pchl3084_0867	PchlO6_0874
Conserved hypothetical protein	Pchl3084_0936	PchlO6_0943
Methyltransferase domain protein	Pchl3084_0968	PchlO6_0976
Lipoprotein, putative	Pchl3084_0996	PchlO6_1004
Lipoprotein, putative	Pchl3084_0998	PchlO6_1006
Hypothetical protein	Pchl3084_1003	PchlO6_1014
Hypothetical protein	Pchl3084_1040	PchlO6_1052
Sensory box histidine kinase/response regulator	Pchl3084_1087	PchlO6_1100
Hypothetical protein	Pchl3084_1100	PchlO6_1111
Conserved hypothetical protein	Pchl3084_1225	PchlO6_1297
Putative TPR repeat protein	Pchl3084_1259	PchlO6_1334
Hypothetical protein	Pchl3084_1301	PchlO6_1376
Membrane protein, putative	Pchl3084_1367	PchlO6_1446
Hypothetical protein	Pchl3084_1368	PchlO6_1447
Conserved hypothetical protein	Pchl3084_1369	PchlO6_1448
Hypothetical protein	Pchl3084_1372	PchlO6_1445
Fimbrial protein, putative	Pchl3084_1392	PchlO6_1862
Lipoprotein, putative	Pchl3084_1399	PchlO6_1470

doi: 10.1371/journal.pgen.1002784

Type I secretion target, putative	Pchl3084_1428	PchlO6_1552
HPt domain protein	Pchl3084_1429	PchlO6_1553
DNA-binding response regulator, LuxR family	Pchl3084_1430	PchlO6_1554
Conserved hypothetical protein	Pchl3084_1460	PchlO6_1584
Outer membrane autotransporter barrel domain protein	Pchl3084_1477	PchlO6_1601
Aromatic ring hydroxylating dioxygenase, beta subunit	Pchl3084_1488	PchlO6_1612
Rieske [2Fe-2S] domain/aromatic ring hydroxylating dioxygenase, alpha subunit domain protein	Pchl3084_1489	PchlO6_1613
Oxidoreductase NAD-binding domain/2Fe-2S iron-sulfur cluster binding domain protein	Pchl3084_1490	PchlO6_1614
Conserved hypothetical protein	Pchl3084_1491	PchlO6_1615
Periplasmic protein	Pchl3084_1529	PchlO6_1660
Hypothetical protein	Pchl3084_1642	PchlO6_1774
Hypothetical protein	Pchl3084_1664	PchlO6_1796
Conserved hypothetical protein	Pchl3084_1734	PchlO6_1864
Lipoprotein, putative	Pchl3084_1786	PchlO6_1918
Conserved hypothetical protein	Pchl3084_1788	PchlO6_1919
Cupin 2 conserved barrel domain protein	Pchl3084_1822	PchlO6_1953
Conserved hypothetical protein	Pchl3084_1824	PchlO6_1957
Conserved hypothetical protein	Pchl3084_1825	PchlO6_1958
Conserved hypothetical protein	Pchl3084_1878	PchlO6_2012
Hypothetical protein	Pchl3084_1883	PchlO6_2017
Hypothetical protein	Pchl3084_1966	PchlO6_2187
Hypothetical protein	Pchl3084_2023	PchlO6_2237
Conserved hypothetical protein	Pchl3084_2072	PchlO6_2279
Antioxidant, AhpC/TSA family	Pchl3084_2080	PchlO6_2293
Hypothetical protein	Pchl3084_2084	PchlO6_2304
Efflux transporter, RND family, MFP subunit	Pchl3084_2085	PchlO6_2305
RND transporter, hydrophobe/amphiphile efflux-1 (HAE1) family	Pchl3084_2086	PchlO6_2306
Alkylhydroperoxidase AhpD family core domain protein	Pchl3084_2094	PchlO6_2314
Integral membrane protein, DUF6 family	Pchl3084_2112	PchlO6_2330
Transcriptional regulator, AraC family	Pchl3084_2113	PchlO6_2331
Conserved hypothetical protein	Pchl3084_2122	PchlO6_2341
Tricarboxylate transporter, TTT family, periplasmic tricarboxylate-binding protein	Pchl3084_2145	PchlO6_2364
Conserved hypothetical protein	Pchl3084_2146	PchlO6_2365
Glyoxalase family protein	Pchl3084_2157	PchlO6_2374
Hypothetical protein	Pchl3084_2183	PchlO6_2400
Putrescine ABC transporter, permease protein	Pchl3084_2197	PchlO6_2420
Conserved hypothetical protein	Pchl3084_2215	PchlO6_2442
Conserved hypothetical protein	Pchl3084_2224	PchlO6_2455
Phosphatidylserine decarboxylase, putative	Pchl3084_2235	PchlO6_2466
Hypothetical protein	Pchl3084_2239	PchlO6_2470
Sensor histidine kinase/response regulator	Pchl3084_2246	PchlO6_2476
Response regulator receiver domain/cyclic	Pchl3084_2247	PchlO6_2477

doi: 10.1371/journal.pgen.1002784

diguanylate phosphodiesterase (EAL) domain protein		
Capsular synthesis regulator component B	Pchl3084_2249	PchlO6_2479
Type I secretion target, putative	Pchl3084_2250	PchlO6_2480
Tetratricopeptide repeat domain protein	Pchl3084_2254	PchlO6_2484
ABC1 family protein	Pchl3084_2307	PchlO6_2528
Conserved hypothetical protein	Pchl3084_2365	PchlO6_2684
Conserved hypothetical protein	Pchl3084_2377	PchlO6_2597
Hypothetical protein	Pchl3084_2382	PchlO6_2602
Phospholipase, patatin family	Pchl3084_2383	PchlO6_2603
Hypothetical protein	Pchl3084_2402	PchlO6_2621
Lipoprotein, putative	Pchl3084_2460	PchlO6_2672
Acetyltransferase, GNAT family	Pchl3084_2462	PchlO6_2675
Lipoprotein, putative	Pchl3084_2469	PchlO6_2682
Hypothetical protein	Pchl3084_2482	PchlO6_2693
Tyrosinase domain protein	Pchl3084_2484	PchlO6_2695
Hypothetical protein	Pchl3084_2509	PchlO6_2727
Cupin region	Pchl3084_2520	PchlO6_2745
Transcriptional regulator, AraC family	Pchl3084_2524	PchlO6_2749
Conserved hypothetical protein	Pchl3084_2534	PchlO6_2763
Endonuclease/exonuclease/phosphatase family protein	Pchl3084_2536	PchlO6_2767
Adenosine specific kinase, putative	Pchl3084_2539	PchlO6_2770
Conserved hypothetical protein	Pchl3084_2540	PchlO6_2772
Membrane protein, putative	Pchl3084_2542	PchlO6_2774
Membrane protein, putative	Pchl3084_2543	PchlO6_2775
Hypothetical protein	Pchl3084_2550	PchlO6_2782
Conserved hypothetical protein	Pchl3084_2552	PchlO6_2784
Cytotoxic domain protein	Pchl3084_2555	PchlO6_2531
Conserved hypothetical protein	Pchl3084_2558	PchlO6_2789
EF hand domain protein	Pchl3084_2575	PchlO6_2808
Na/Pi-cotransporter II-related protein, putative	Pchl3084_2593	PchlO6_2825
Conserved hypothetical protein	Pchl3084_2594	PchlO6_2826
Diguanylate cyclase (GGDEF) domain protein	Pchl3084_2612	PchlO6_2847
Acetyltransferase, GNAT family	Pchl3084_2657	PchlO6_2886
Hypothetical protein	Pchl3084_2658	PchlO6_2888
Hypothetical protein	Pchl3084_2661	PchlO6_2891
Hypothetical protein	Pchl3084_2690	PchlO6_2921
Lipoprotein, putative	Pchl3084_2756	PchlO6_2988
Conserved hypothetical protein	Pchl3084_2782	PchlO6_3014
Conserved hypothetical protein	Pchl3084_2887	PchlO6_3141
Xanthine and CO dehydrogenases maturation factor	Pchl3084_2913	PchlO6_3189
Oxidoreductase, short chain dehydrogenase/reductase family	Pchl3084_2919	PchlO6_3188
Oxidoreductase, short chain dehydrogenase/reductase family	Pchl3084_2970	PchlO6_2843
Oxidoreductase, GMC family	Pchl3084_2985	PchlO6_3237
Lipoprotein, putative	Pchl3084_2986	PchlO6_3238
Phytanoyl-CoA dioxygenase, PhyH family	Pchl3084_2996	PchlO6_3268
Transcriptional regulator, AraC family	Pchl3084_2997	PchlO6_3269

doi: 10.1371/journal.pgen.1002784

Hypothetical protein	Pchl3084_3010	PchlO6_3274
Conserved hypothetical protein	Pchl3084_3013	PchlO6_3276
Conserved hypothetical protein	Pchl3084_3025	PchlO6_3285
Pyocin immunity protein	Pchl3084_3029	PchlO6_3287
Outer membrane porin, OprD family	Pchl3084_3064	PchlO6_3325
Conserved hypothetical protein	Pchl3084_3066	PchlO6_3327
Aldehyde dehydrogenase (NAD) family protein	Pchl3084_3083	PchlO6_3344
Sensor histidine kinase/response regulator	Pchl3084_3110	PchlO6_3368
Chitinase	Pchl3084_3180	PchlO6_3409
Chitin binding domain protein	Pchl3084_3181	PchlO6_3410
Conserved hypothetical protein	Pchl3084_3222	PchlO6_3451
Hypothetical protein	Pchl3084_3285	PchlO6_3515
Fimbrial protein, putative	Pchl3084_3309	PchlO6_3539
Conserved hypothetical protein	Pchl3084_3311	PchlO6_3541
Conserved hypothetical protein	Pchl3084_3313	PchlO6_3542
Conserved hypothetical protein	Pchl3084_3314	PchlO6_3544
Acetyltransferase, GNAT family	Pchl3084_3315	PchlO6_3545
Conserved hypothetical protein	Pchl3084_3317	PchlO6_3548
Protein of unknown function, DUF336 family	Pchl3084_3337	PchlO6_3607
Transcriptional regulator, TetR family	Pchl3084_3339	PchlO6_3609
Hypothetical protein	Pchl3084_3340	PchlO6_3610
Integral membrane protein, DUF6 family	Pchl3084_3341	PchlO6_3611
Thioesterase superfamily protein	Pchl3084_3343	PchlO6_3613
Transporter, monovalent cation:proton antiporter-2 (CPA2) family	Pchl3084_3344	PchlO6_3614
RND transporter, hydrophobe/amphiphile efflux-1 (HAE1) family	Pchl3084_3346	PchlO6_3617
Efflux transporter, RND family, MFP subunit	Pchl3084_3347	PchlO6_3618
Redox-sensitive transcriptional activator SoxR	Pchl3084_3348	PchlO6_3619
Conserved hypothetical protein	Pchl3084_3354	PchlO6_3656
Tetratricopeptide repeat domain protein	Pchl3084_3364	PchlO6_3675
Conserved hypothetical protein	Pchl3084_3366	PchlO6_3677
T1SS-143 repeat domain protein	Pchl3084_3389	PchlO6_3714
T1SS-143 repeat domain protein	Pchl3084_3390	PchlO6_3715
Transcriptional regulator, LuxR family	Pchl3084_3391	PchlO6_3716
Type VI secretion protein Fha1	Pchl3084_3413	PchlO6_3745
Conserved hypothetical protein	Pchl3084_3449	PchlO6_0773
DsbA-like thioredoxin domain protein	Pchl3084_3453	PchlO6_3783
Cytochrome c family protein	Pchl3084_3456	PchlO6_3786
2Fe-2S iron-sulfur cluster binding domain protein	Pchl3084_3457	PchlO6_3787
Aldehyde oxidase and xanthine dehydrogenase family, molybdopterin-binding domain protein	Pchl3084_3458	PchlO6_3788
Sensor histidine kinase/response regulator	Pchl3084_3460	PchlO6_3791
Conserved hypothetical protein	Pchl3084_3513	PchlO6_3823
Methyltransferase domain family	Pchl3084_3514	PchlO6_3825
Glutamate synthase domain protein	Pchl3084_3516	PchlO6_3827
Conserved hypothetical protein	Pchl3084_3521	PchlO6_3830
Protein of unknown function, DUF307 family	Pchl3084_3522	PchlO6_3831
Type I secretion system ATPase FitA	Pchl3084_3530	PchlO6_3841

doi: 10.1371/journal.pgen.1002784

Lipoprotein, putative	Pchl3084_3544	PchlO6_3846
DsbA-like thioredoxin domain protein	Pchl3084_3578	PchlO6_3889
Conserved hypothetical protein	Pchl3084_3623	PchlO6_3930
Hypothetical protein	Pchl3084_3649	PchlO6_3942
Hypothetical protein	Pchl3084_3650	PchlO6_3943
Conserved hypothetical protein	Pchl3084_3660	PchlO6_3953
Conserved hypothetical protein	Pchl3084_3663	PchlO6_3956
Conserved hypothetical protein	Pchl3084_3826	PchlO6_4121
Hypothetical protein	Pchl3084_3837	PchlO6_4127
Type I secretion membrane fusion protein, HlyD family	Pchl3084_3839	PchlO6_4128
Pyocin AP41, large subunit, putative	Pchl3084_3846	PchlO6_4136
DNA/RNA non-specific endonuclease	Pchl3084_3851	PchlO6_4141
Conserved hypothetical protein	Pchl3084_3856	PchlO6_4146
Conserved hypothetical protein	Pchl3084_3882	PchlO6_4174
Conserved hypothetical protein	Pchl3084_3899	PchlO6_4176
Glutathione S-transferase domain protein	Pchl3084_3910	PchlO6_4188
Conserved hypothetical protein	Pchl3084_3932	PchlO6_4215
Acyl carrier protein	Pchl3084_3966	PchlO6_4242
Beta-ketoacyl-acyl-carrier-protein synthase III, putative	Pchl3084_3967	PchlO6_4243
Dialkylrecorsinol condensing enzyme	Pchl3084_3968	PchlO6_4244
Transcriptional regulator, ArsR family	Pchl3084_3972	PchlO6_4248
Conserved hypothetical protein	Pchl3084_3991	PchlO6_4257
D-isomer specific 2-hydroxyacid dehydrogenase family protein	Pchl3084_4012	PchlO6_4269
Conserved hypothetical protein	Pchl3084_4059	PchlO6_4313
Pyoverdine biosynthesis protein	Pchl3084_4065	PchlO6_4319
Conserved hypothetical protein	Pchl3084_4082	PchlO6_4336
Conserved hypothetical protein	Pchl3084_4372	PchlO6_4610
Hypothetical protein	Pchl3084_4373	PchlO6_4611
dTDP-6-deoxy-3,4-keto-hexulose isomerase	Pchl3084_4374	PchlO6_4612
Conserved hypothetical protein	Pchl3084_4379	PchlO6_4616
Hypothetical protein	Pchl3084_4411	PchlO6_4649
Carbohydrate-selective porin OprB	Pchl3084_4484	PchlO6_4725
Type IVb pilin, Flp family	Pchl3084_4532	PchlO6_4774
Conserved hypothetical protein	Pchl3084_4597	PchlO6_4843
Transcriptional regulator, LuxR family	Pchl3084_4696	PchlO6_4942
PAAR motif domain protein	Pchl3084_4790	PchlO6_5040
Hypothetical protein	Pchl3084_4795	PchlO6_5046
Amidohydrolase family protein	Pchl3084_4796	PchlO6_5047
Conserved hypothetical protein	Pchl3084_4798	PchlO6_5051
Fatty acid desaturase domain protein	Pchl3084_4801	PchlO6_5054
Oxidoreductase, FAD/NAD-binding domains/2Fe-2S iron-sulfur cluster binding domain protein	Pchl3084_4802	PchlO6_5055
Fatty acid desaturase domain protein	Pchl3084_4803	PchlO6_5056
Conserved hypothetical protein	Pchl3084_4804	PchlO6_5057
Aminotransferase, class III	Pchl3084_4805	PchlO6_5058
Phosphopantetheine attachment site domain protein	Pchl3084_4806	PchlO6_5059

doi: 10.1371/journal.pgen.1002784

Transcriptional regulator, LuxR family	Pchl3084_4807	PchlO6_5060
Fatty acid desaturase domain protein	Pchl3084_4810	PchlO6_5063
Conserved hypothetical protein	Pchl3084_4835	PchlO6_5087
Autoinducer synthase PhzI	Pchl3084_4949	PchlO6_5218
Transcriptional activator protein PhzR	Pchl3084_4950	PchlO6_5219
Phenazine biosynthesis protein PhzO	Pchl3084_4958	PchlO6_5227
Transcriptional regulator, GntR family/aminotransferase, classes I and II family protein	Pchl3084_5154	PchlO6_5421
Conserved hypothetical protein	Pchl3084_5497	PchlO6_5779
Conserved hypothetical protein	Pchl3084_5553	PchlO6_5833
Adenylate cyclase family protein	Pchl3084_5667	PchlO6_5945
Conserved hypothetical protein	Pchl3084_5671	PchlO6_5949
Hypothetical protein	Pchl3084_5675	PchlO6_5953
Hypothetical protein	Pchl3084_5676	PchlO6_5954
Conserved hypothetical protein	Pchl3084_5682	PchlO6_5960
Hypothetical protein	Pchl3084_5738	PchlO6_6015
Conserved hypothetical protein	Pchl3084_5749	PchlO6_6026
Conserved hypothetical protein	Pchl3084_5751	PchlO6_6028
Conserved hypothetical protein	Pchl3084_5752	PchlO6_6029
HAD-superfamily hydrolase	Pchl3084_5944	PchlO6_6293
Transporter, major facilitator family	Pchl3084_5945	PchlO6_6294
Conserved hypothetical protein	Pchl3084_5946	PchlO6_6295
Nudix-type nucleoside diphosphatase, YffH/AdpP family	Pchl3084_5947	PchlO6_6296
Methylthioadenosine phosphorylase	Pchl3084_5948	PchlO6_6297
Putative dihydrorhizobitoxine desaturase	Pchl3084_5949	PchlO6_6298
Haloacid dehalogenase domain protein hydrolase	Pchl3084_5950	PchlO6_6299
FAD dependent oxidoreductase	Pchl3084_5951	PchlO6_6300
Aminotransferase, DegT/DnrJ/EryC1/StrS family	Pchl3084_5952	PchlO6_6301
Hydrolase, nudix family, putative	Pchl3084_5953	PchlO6_6302
Aminotransferase, DegT/DnrJ/EryC1/StrS family	Pchl3084_5954	PchlO6_6303
Oxidoreductase, Gfo/Idh/MocA family	Pchl3084_5955	PchlO6_6304

[a] Genes are present in genomes of the strains 30-84 and O6 but are not present in the genomes of other *Pseudomonas* spp. in Figure 1.

doi: 10.1371/journal.pgen.1002784
