# Supplementary material for: Comparative Genomics of Plant-Associated Pseudomonas spp.: Insights into Diversity and Inheritance of Traits Involved in Multitrophic Interactions
Source: PLoS Genet. 2012 Jul 5;8(7):e1002784. doi: 10.1371/journal.pgen.1002784 (PMC3390384; doi:10.1371/journal.pgen.1002784)
Supplement: Table S7 — Genes shared by and unique to strains in Sub-clade 2 of the P. fluorescens group. Locus tags represent CDSs conserved among the genomes of strains Pf0-1, Q8r1-96, and Q2-87, but absent from the genomes of all other representative Pseudomonas spp. These CDSs were identified from comparative BLASTp searches of the predicted proteomes of representative Pseudomonas spp. (shown in Figure 1). (PDF) [file pgen.1002784.s017.pdf]

Table S7. Genes shared by and unique to Sub-clade 2[a]

Annotated function	Pf0-1	Q8r1-96	Q2-87
Nodulation efficiency protein NfeD	Pfl01_0167	PflQ8_0241	PflQ2_5495
Conserved hypothetical protein	Pfl01_0296	PflQ8_0328	PflQ2_5408
GCN5-related N-acetyltransferase	Pfl01_0297	PflQ8_0329	PflQ2_5407
Putative transport protein, AzlC family	Pfl01_0298	PflQ8_0330	PflQ2_5406
Transcriptional regulator, LysR family	Pfl01_0299	PflQ8_0331	PflQ2_5405
Conserved hypothetical protein	Pfl01_0314	PflQ8_0348	PflQ2_5379
PhoD family protein	Pfl01_0461	PflQ8_0502	PflQ2_5204
Putative toxin protein	Pfl01_0608	PflQ8_0649	PflQ2_5060
Sensory box domain protein	Pfl01_1029	PflQ8_1019	PflQ2_4448
Amino acid permease, APC family	Pfl01_1043	PflQ8_2343	PflQ2_2349
Conserved domain protein	Pfl01_1378	PflQ8_1403	PflQ2_4061
Methyltransferase domain family	Pfl01_1380	PflQ8_1405	PflQ2_4059
GtrA family protein	Pfl01_1381	PflQ8_1406	PflQ2_4058
Membrane protein, putative	Pfl01_1382	PflQ8_1407	PflQ2_4057
Conserved hypothetical protein	Pfl01_1838	PflQ8_3978	PflQ2_1662
DNA-binding response regulator, LuxR family	Pfl01_1863	PflQ8_3955	PflQ2_1687
Conserved hypothetical protein	Pfl01_1930	PflQ8_3789	PflQ2_1843
Conserved hypothetical protein	Pfl01_2012	PflQ8_3850	PflQ2_1785
Transcriptional regulator, AraC family	Pfl01_2224	PflQ8_2504	PflQ2_2942
FAD dependent oxidoreductase	Pfl01_2315	PflQ8_4787	PflQ2_0863
Hydrolase, alpha/beta fold family	Pfl01_2417	PflQ8_0455	PflQ2_5256
Conserved hypothetical protein	Pfl01_2418	PflQ8_0762	PflQ2_0684
Response regulator receiver domain protein	Pfl01_2452	PflQ8_2749	PflQ2_3033
Delta-60 repeat domain protein	Pfl01_2590	PflQ8_3163	PflQ2_2476
Protease inhibitor Inh	Pfl01_2679	PflQ8_2859	PflQ2_2792
Conserved hypothetical protein	Pfl01_3070	PflQ8_2119	PflQ2_3298
Lipase	Pfl01_3071	PflQ8_2118	PflQ2_3299
Conserved hypothetical protein	Pfl01_3338	PflQ8_2586	PflQ2_0015
Type VI secretion system Vgr family protein	Pfl01_3678	PflQ8_1955	PflQ2_3488
Conserved hypothetical protein	Pfl01_3959	PflQ8_1719	PflQ2_3760
Conserved hypothetical protein	Pfl01_4132	PflQ8_4097	PflQ2_1546
Transcriptional regulator, GntR family	Pfl01_4144	PflQ8_4104	PflQ2_1539
Tat (twin-arginine translocation) pathway signal sequence domain protein	Pfl01_4469	PflQ8_4593	PflQ2_1056
Membrane protein, putative	Pfl01_4512	PflQ8_4475	PflQ2_1171
Autoinducer-binding transcriptional regulator, LuxR family	Pfl01_4889	PflQ8_5023	PflQ2_4761
Conserved hypothetical protein	Pfl01_5217	PflQ8_5380	PflQ2_0504
Conserved hypothetical protein	Pfl01_5218	PflQ8_5381	PflQ2_0503
Oxidoreductase, GMC family	Pfl01_5219	PflQ8_5382	PflQ2_0502

[a] Genes are present in genomes of the strains found in Sub-clade 2, but are not present in the genomes of other *Pseudomonas* spp. in Figure 1.

doi: 10.1371/journal.pgen.1002784
