# Supplementary material for: Comparative Genomics of Plant-Associated Pseudomonas spp.: Insights into Diversity and Inheritance of Traits Involved in Multitrophic Interactions
Source: PLoS Genet. 2012 Jul 5;8(7):e1002784. doi: 10.1371/journal.pgen.1002784 (PMC3390384; doi:10.1371/journal.pgen.1002784)
Supplement: Table S8 — Genes shared by and unique to strains Q8r1-96 and Q2-87. Locus tags represent CDSs conserved among the genomes of strains Q8r1-96, and Q2-87, but absent from the genomes of all other representative Pseudomonas spp. These CDSs were identified from comparative BLASTp searches of the predicted proteomes of representative Pseudomonas spp. (shown in Figure 1). (PDF) [file pgen.1002784.s018.pdf]

Table S8. Genes shared by and unique to Q8r1-96 and Q2-87[a]

**Annotated function**	**Q8r1-96**	**Q2-87**
Putative type I secretion target	PflQ8_0085	PflQ2_1985
Conserved hypothetical protein	PflQ8_0086	PflQ2_0917
Conserved hypothetical protein	PflQ8_0174	PflQ2_5559
Hypothetical protein	PflQ8_0216	PflQ2_5520
Transcriptional regulator, LysR family	PflQ8_0335	PflQ2_5398
Methyl-accepting chemotaxis protein	PflQ8_0414	PflQ2_5310
Hypothetical protein	PflQ8_0454	PflQ2_5249
Conserved hypothetical protein	PflQ8_0483	PflQ2_5223
Conserved hypothetical protein	PflQ8_0486	PflQ2_1813
Conserved hypothetical protein	PflQ8_0542	PflQ2_5167
Hypothetical protein	PflQ8_0660	PflQ2_5049
Conserved hypothetical protein	PflQ8_0761	PflQ2_3994
Conserved hypothetical protein	PflQ8_0763	PflQ2_0685
Conserved hypothetical protein	PflQ8_0818	PflQ2_0731
Conserved hypothetical protein	PflQ8_0838	PflQ2_0754
Conserved hypothetical protein	PflQ8_0919	PflQ2_4553
Hypothetical protein	PflQ8_0920	PflQ2_4552
Hypothetical protein	PflQ8_0925	PflQ2_4537
Conserved hypothetical protein	PflQ8_0929	PflQ2_4536
Hypothetical protein	PflQ8_0930	PflQ2_4535
Hypothetical protein	PflQ8_0939	PflQ2_4525
Hypothetical protein	PflQ8_0940	PflQ2_4524
Conserved hypothetical protein	PflQ8_0997	PflQ2_4467
Transcriptional regulator, AraC family	PflQ8_0999	PflQ2_2314
Hypothetical protein	PflQ8_1026	PflQ2_4441
Type III effector protein RopAA	PflQ8_1079	PflQ2_4389
Conserved hypothetical protein	PflQ8_1124	PflQ2_4342
Hypothetical protein	PflQ8_1207	PflQ2_4158
Conserved hypothetical protein	PflQ8_1312	PflQ2_4157
Aminotransferase class I and II	PflQ8_1339	PflQ2_4130
Diguanylate cyclase (GGDEF) domain protein	PflQ8_1356	PflQ2_4112
LysM domain protein	PflQ8_1496	PflQ2_3966
Nucleotide sugar epimerase/dehydratase WbpM	PflQ8_1633	PflQ2_3841
Hypothetical protein	PflQ8_1858	PflQ2_3593
ABC transporter, permease protein	PflQ8_1963	PflQ2_3480
Oxidoreductase, short chain dehydrogenase/reductase family	PflQ8_2028	PflQ2_3416
Hypothetical protein	PflQ8_2044	PflQ2_2044
Conserved hypothetical protein	PflQ8_2070	PflQ2_2081
2-dehydro-3-deoxygluconokinase	PflQ8_2105	PflQ2_3314
Methyl-accepting chemotaxis protein	PflQ8_2162	PflQ2_2316
Rieske [2Fe-2S] domain protein	PflQ8_2165	PflQ2_2319
Vanillate monooxygenase family protein	PflQ8_2172	PflQ2_2326
Vanillate monooxygenase family protein	PflQ8_2173	PflQ2_2327
Vanillate O-demethylase oxidoreductase	PflQ8_2174	PflQ2_2328
Succinate-semialdehyde dehydrogenase [NAD(P)+]	PflQ8_2182	PflQ2_2337
Conserved hypothetical protein	PflQ8_2184	PflQ2_2339

doi: 10.1371/journal.pgen.1002784

Hypothetical protein	PflQ8_2201	PflQ2_3228
Conserved hypothetical protein	PflQ8_2238	PflQ2_4245
Hypothetical protein	PflQ8_2239	PflQ2_4244
Sigma-54 dependent transcriptional regulator	PflQ8_2324	PflQ2_2270
Conserved hypothetical protein	PflQ8_2328	PflQ2_2244
Conserved hypothetical protein	PflQ8_2340	PflQ2_2346
Transcriptional regulator, GntR family	PflQ8_2341	PflQ2_2347
Aldehyde dehydrogenase (NAD) family protein	PflQ8_2342	PflQ2_2348
Conserved domain protein	PflQ8_2359	PflQ2_2241
Transporter, major facilitator family	PflQ8_2364	PflQ2_2248
Starvation-sensing protein RspA	PflQ8_2405	PflQ2_2388
Transporter, major facilitator family	PflQ8_2407	PflQ2_2390
D-mannonate dehydratase	PflQ8_2409	PflQ2_2392
Transcriptional regulator, GntR family	PflQ8_2410	PflQ2_2564
Conserved hypothetical protein	PflQ8_2412	PflQ2_2566
Conserved hypothetical protein	PflQ8_2418	PflQ2_2570
ATPase/histidine kinase/DNA gyrase B/HSP90 domain protein	PflQ8_2419	PflQ2_2571
Conserved hypothetical protein	PflQ8_2459	PflQ2_2593
Diguanylate cyclase (GGDEF) domain protein	PflQ8_2460	PflQ2_2594
Conserved hypothetical protein	PflQ8_2461	PflQ2_2595
Conserved hypothetical protein	PflQ8_2476	PflQ2_2609
Hypothetical protein	PflQ8_2477	PflQ2_2610
Putative polyketide synthase	PflQ8_2478	PflQ2_2611
Phosphopantetheine attachment site domain protein	PflQ8_2479	PflQ2_2612
FAD binding domain protein	PflQ8_2480	PflQ2_2613
HAD hydrolase, family IB	PflQ8_2481	PflQ2_2614
AMP-binding domain protein	PflQ8_2482	PflQ2_2615
Transcriptional regulator, TetR family	PflQ8_2484	PflQ2_2617
ABC transporter, substrate-binding protein, QAT family	PflQ8_2488	PflQ2_2958
Conserved hypothetical protein	PflQ8_2490	PflQ2_2956
Glutathione S-transferase	PflQ8_2517	PflQ2_2907
Transcriptional regulator, LysR family	PflQ8_2519	PflQ2_2904
Alkylhydroperoxidase AhpD family core domain protein	PflQ8_2521	PflQ2_2903
Sensory box histidine kinase	PflQ8_2577	PflQ2_2875
Conserved hypothetical protein	PflQ8_2580	PflQ2_2350
Hypothetical protein	PflQ8_2600	PflQ2_2853
Epoxide hydrolase	PflQ8_2619	PflQ2_2845
Hypothetical protein	PflQ8_2638	PflQ2_2554
FMN reductase MsuE	PflQ8_2643	PflQ2_2557
Response regulator	PflQ8_2655	PflQ2_2397
Sensory box histidine kinase	PflQ8_2656	PflQ2_2398
Conserved hypothetical protein	PflQ8_2657	PflQ2_2399
Type I secretion system ATPase, AprD family	PflQ8_2664	PflQ2_2406
Type I secretion membrane fusion protein, AprE family	PflQ8_2665	PflQ2_2407
Type I secretion outer membrane protein, TolC family	PflQ8_2666	PflQ2_2408
Conserved hypothetical protein	PflQ8_2713	PflQ2_2451
Conserved hypothetical protein	PflQ8_2714	PflQ2_2452
Patched family protein	PflQ8_2716	PflQ2_2454

doi: 10.1371/journal.pgen.1002784

Conserved hypothetical protein	PflQ8_2721	PflQ2_3057
Conserved hypothetical protein	PflQ8_2780	PflQ2_2998
TRAP transporter, DctQ-like membrane protein	PflQ8_2786	PflQ2_2992
Transporter, major facilitator family	PflQ8_2791	PflQ2_2987
NAD dependent epimerase/dehydratase family protein	PflQ8_2792	PflQ2_2986
Hypothetical protein	PflQ8_2845	PflQ2_2652
ATPase/histidine kinase/DNA gyrase B/HSP90 domain protein	PflQ8_2851	PflQ2_2659
Band 7 protein	PflQ8_2852	PflQ2_2660
Phosphoglycerate mutase family protein	PflQ8_2958	PflQ2_3278
Conserved hypothetical protein	PflQ8_2964	PflQ2_2730
Transcriptional regulator, LysR family	PflQ8_2965	PflQ2_2729
NAD dependent epimerase/dehydratase family protein	PflQ8_2981	PflQ2_2716
Leucine Rich Repeat domain protein	PflQ8_3011	PflQ2_2698
Periplasmic arginine/ornithine-binding protein	PflQ8_3020	PflQ2_2693
Conserved hypothetical protein	PflQ8_3023	PflQ2_2690
Histidine ABC transporter, ATP-binding protein HisP	PflQ8_3067	PflQ2_2822
Histidine ABC transporter, permease protein HisM	PflQ8_3068	PflQ2_2823
Histidine ABC transporter, permease protein HisQ	PflQ8_3069	PflQ2_2824
Lysine/arginine/ornithine ABC transporter, periplasmic lysine/arginine/ornithine-binding protein ArgT	PflQ8_3070	PflQ2_2825
Enzyme, Cys/Met metabolism PLP-dependent family	PflQ8_3072	PflQ2_2827
Aldehyde dehydrogenase (NAD) family protein	PflQ8_3073	PflQ2_2828
Putative threonine synthase	PflQ8_3076	PflQ2_2830
DNA-binding domain/cupin domain protein	PflQ8_3079	PflQ2_2832
Polyol dehydrogenase MtlD	PflQ8_3133	PflQ2_2521
DNA ligase D	PflQ8_3142	PflQ2_2512
Nitrate/nitrite sensor protein NarX	PflQ8_3198	PflQ2_3073
Conserved hypothetical protein	PflQ8_3211	PflQ2_3084
Sensory box domain/GGDEF domain/EAL domain protein	PflQ8_3273	PflQ2_3144
Type II secretion protein, secretin HxcQ	PflQ8_3278	PflQ2_3149
Putative lipoprotein	PflQ8_3297	PflQ2_3534
Conserved hypothetical protein	PflQ8_3312	PflQ2_2210
Hypothetical protein	PflQ8_3369	PflQ2_2157
Hypothetical protein	PflQ8_3554	PflQ2_1976
Non-heme chloroperoxidase	PflQ8_3557	PflQ2_1975
Transcriptional regulator, AsnC family	PflQ8_3558	PflQ2_1974
Conserved hypothetical protein	PflQ8_3596	PflQ2_1942
NmrA family protein	PflQ8_3597	PflQ2_1941
Alginate lyase family protein	PflQ8_3601	PflQ2_1935
Nucleoside:H+ symporter family protein	PflQ8_3602	PflQ2_1934
Oxidoreductase, NAD-binding Rossman domain family	PflQ8_3604	PflQ2_1932
Transcriptional regulator, LacI family	PflQ8_3605	PflQ2_1931
Isochorismatase family protein	PflQ8_3615	PflQ2_1926
Transcriptional regulator, TetR family	PflQ8_3664	PflQ2_3213
Hypothetical protein	PflQ8_3679	PflQ2_1861
Hypothetical protein	PflQ8_3684	PflQ2_3216
ABC transporter, substrate-binding protein, QAT family	PflQ8_3686	PflQ2_3217
NADP oxidoreductase, coenzyme F420-dependent	PflQ8_3692	PflQ2_3220

Transcriptional regulator, LysR family	PflQ8_3696	PflQ2_3223
Rieske [2Fe-2S] domain protein	PflQ8_3697	PflQ2_3224
Oxidoreductase, FAD/NAD-binding domains/2Fe-2S iron-sulfur cluster binding domain protein	PflQ8_3698	PflQ2_3225
Hypothetical protein	PflQ8_3735	PflQ2_1857
Hypothetical protein	PflQ8_3831	PflQ2_1804
Acetyltransferase, GNAT family	PflQ8_3876	PflQ2_1977
Hypothetical protein	PflQ8_3891	PflQ2_1983
Conserved hypothetical protein	PflQ8_4031	PflQ2_1611
Conserved hypothetical protein	PflQ8_4033	PflQ2_1608
Glyoxalase family protein	PflQ8_4049	PflQ2_1593
Conserved hypothetical protein	PflQ8_4052	PflQ2_1587
Conserved hypothetical protein	PflQ8_4053	PflQ2_1586
Transcriptional regulator, AsnC family	PflQ8_4165	PflQ2_1476
Translocator protein, LysE family	PflQ8_4170	PflQ2_1475
Transcriptional regulator, GntR family/aminotransferase, classes I and II family protein	PflQ8_4177	PflQ2_1468
Conserved hypothetical protein	PflQ8_4256	PflQ2_1384
Transporter, Ompp1/FadL/TodX family	PflQ8_4309	PflQ2_1333
Conserved hypothetical protein	PflQ8_4413	PflQ2_1232
Peptidase propeptide and YpeB domain protein	PflQ8_4430	PflQ2_1215
Conserved hypothetical protein	PflQ8_4434	PflQ2_1212
Conserved hypothetical protein	PflQ8_4479	PflQ2_1167
Polyamine ABC transporter, ATP-binding protein	PflQ8_4590	PflQ2_1060
Hypothetical protein	PflQ8_4605	PflQ2_0158
Conserved hypothetical protein	PflQ8_4615	PflQ2_1031
Conserved hypothetical protein	PflQ8_4621	PflQ2_1025
Hypothetical protein	PflQ8_4648	PflQ2_1000
Oxidoreductase, short chain dehydrogenase/reductase family	PflQ8_4852	PflQ2_4593
Conserved hypothetical protein	PflQ8_4973	PflQ2_4714
Hypothetical protein	PflQ8_5010	PflQ2_4752
Conserved hypothetical protein	PflQ8_5126	PflQ2_4862
Conserved hypothetical protein	PflQ8_5139	PflQ2_1679
Tetratricopeptide repeat domain protein	PflQ8_5198	PflQ2_4936
Conserved hypothetical protein	PflQ8_5199	PflQ2_4938
Hypothetical protein	PflQ8_5200	PflQ2_4939
Hypothetical protein	PflQ8_5201	PflQ2_2743
Translation elongation factor Tu	PflQ8_5235	PflQ2_4974
Carboxylesterase	PflQ8_5358	PflQ2_2821
Hypothetical protein	PflQ8_5365	PflQ2_0525
Hypothetical protein	PflQ8_5366	PflQ2_0522
Conserved hypothetical protein	PflQ8_5424	PflQ2_0464
Type III secretion apparatus protein RspA	PflQ8_5530	PflQ2_0353
Harpin RspZ	PflQ8_5531	PflQ2_0352
Type III secretion protein RspF	PflQ8_5536	PflQ2_0347
Type III secretion protein RspT	PflQ8_5539	PflQ2_0344
Putative type III effector protein RopB	PflQ8_5542	PflQ2_0341
Type III secretion protein RspP	PflQ8_5548	PflQ2_0335

doi: 10.1371/journal.pgen.1002784

Type III secretion protein RspO	PflQ8_5549	PflQ2_0334
Acetyltransferase, GNAT family	PflQ8_5555	PflQ2_0328
Putative beta-lactamase	PflQ8_5557	PflQ2_0326
Putative membrane-bound lytic murein transglycosylase RspH	PflQ8_5558	PflQ2_0325
Type III effector protein RopM	PflQ8_5560	PflQ2_0323
Uroporphyrinogen-III synthase/HemX protein	PflQ8_5644	PflQ2_0233
Hypothetical protein	PflQ8_5754	PflQ2_5627
Transcriptional regulator, LysR family	PflQ8_5803	PflQ2_5679

[a] Genes are present in genomes of the strains Q8r1-96 and Q2-87 but are not present in the genomes of other *Pseudomonas* spp. in Figure 1.

doi: 10.1371/journal.pgen.1002784
