# Supplementary material for: Comparative Genomics of Plant-Associated Pseudomonas spp.: Insights into Diversity and Inheritance of Traits Involved in Multitrophic Interactions
Source: PLoS Genet. 2012 Jul 5;8(7):e1002784. doi: 10.1371/journal.pgen.1002784 (PMC3390384; doi:10.1371/journal.pgen.1002784)
Supplement: Table S9 — Genes shared by and unique to strains in Sub-clade 3 of the P. fluorescens group. Locus tags represent CDSs conserved among the genomes of strains BG33R, SBW25, A506, and SS101, but absent from the genomes of all other representative Pseudomonas spp. These CDSs were identified from comparative BLASTp searches of the predicted proteomes of representative Pseudomonas spp. (shown in Figure 1). (PDF) [file pgen.1002784.s019.pdf]

Table S9. Genes shared by and unique to Sub-clade 3[a]

Annotated function	BG33R	SBW25	A506	SS101
Conserved hypothetical protein	PseBG33_0269	PFLU0235	PflA506_0235	PflSS101_0251
Amino acid ABC transporter, periplasmic amino acid-binding protein	PseBG33_0396	PFLU0375	PflA506_0360	PflSS101_0398
Methyl-accepting chemotaxis domain protein	PseBG33_0400	PFLU0379	PflA506_0364	PflSS101_0402
Conserved hypothetical protein	PseBG33_0461	PFLU0440	PflA506_0432	PflSS101_0463
Conserved hypothetical protein	PseBG33_0557	PFLU0540	PflA506_0528	PflSS101_0557
Conserved hypothetical protein	PseBG33_0625	PFLU0608	PflA506_0589	PflSS101_0617
Putative acetyl-CoA acetyltransferase	PseBG33_0669	PFLU0655	PflA506_0633	PflSS101_0661
Type III secretion protein RspO	PseBG33_0736	PFLU0712	PflA506_0699	PflSS101_0729
Type III secretion protein RspP	PseBG33_0737	PFLU0713	PflA506_0700	PflSS101_0730
Type III secretion system negative regulatory protein RspV	PseBG33_0743	PFLU0719	PflA506_0706	PflSS101_0736
Type III secretion protein RspT	PseBG33_0744	PFLU0720	PflA506_0707	PflSS101_0737
Putative amino acid ABC transporter, periplasmic amino acid-binding protein	PseBG33_0983	PFLU0963	PflA506_0944	PflSS101_0980
Conserved hypothetical protein	PseBG33_1029	PFLU1008	PflA506_0987	PflSS101_1028
Conserved hypothetical protein	PseBG33_1031	PFLU1009	PflA506_0989	PflSS101_1030
Gram-negative pili assembly chaperone, N-terminal domain protein	PseBG33_1033	PFLU1011	PflA506_0991	PflSS101_1032
Conserved hypothetical protein	PseBG33_1034	PFLU1012	PflA506_0992	PflSS101_1033
Conserved hypothetical protein	PseBG33_1035	PFLU1013	PflA506_0993	PflSS101_1034
Conserved hypothetical protein	PseBG33_1036	PFLU1015	PflA506_0994	PflSS101_1035
Conserved hypothetical protein	PseBG33_1273	PFLU1206	PflA506_1181	PflSS101_1219
Conserved hypothetical protein	PseBG33_1633	PFLU1527	PflA506_1543	PflSS101_1549
Conserved hypothetical protein	PseBG33_1652	PFLU1547	PflA506_1561	PflSS101_1567
Transcriptional regulator, AbrB family	PseBG33_1709	PFLU1618	PflA506_1671	PflSS101_1623
Beta-hydroxylase, aspartyl/asparaginyl family	PseBG33_1711	PFLU1620	PflA506_1673	PflSS101_1625
Transcriptional regulator, LysR family	PseBG33_1716	PFLU1625	PflA506_1678	PflSS101_1630
Drug resistance transporter, Bcr/CflA family	PseBG33_1717	PFLU1626	PflA506_1679	PflSS101_1631
Conserved hypothetical protein	PseBG33_1779	PFLU1695	PflA506_1743	PflSS101_1702
Extracellular metalloproteinase, serralysin family	PseBG33_1851	PFLU1793	PflA506_1814	PflSS101_1775
Putative type III secretion effector protein	PseBG33_2028	PFLU2099	PflA506_1996	PflSS101_1952
Putative type III secretion effector protein	PseBG33_2030	PFLU2101	PflA506_1998	PflSS101_1954
Transporter, major facilitator family	PseBG33_2089	PFLU4214	PflA506_2135	PflSS101_2015
Sensor histidine kinase/response regulator	PseBG33_2100	PFLU2181	PflA506_2147	PflSS101_2026
Conserved hypothetical protein	PseBG33_2321	PFLU4167	PflA506_2188	PflSS101_2068
Transcriptional regulator, LysR family	PseBG33_2326	PFLU2291	PflA506_2223	PflSS101_2114

doi: 10.1371/journal.pgen.1002784

Ribose ABC transporter, periplasmic ribose-binding protein	PseBG33_2375	PFLU3996	PflA506_3373	PflSS101_3383
Ribose ABC transporter, ATP-binding protein	PseBG33_2376	PFLU3995	PflA506_3372	PflSS101_3382
Carbohydrate kinase, FGGY family	PseBG33_2379	PFLU3992	PflA506_3369	PflSS101_3379
Sugar binding transcriptional regulator, LacI family	PseBG33_2380	PFLU3990	PflA506_3368	PflSS101_3378
Short chain dehydrogenase/reductase family protein	PseBG33_2381	PFLU3991	PflA506_3367	PflSS101_3377
Conserved hypothetical protein	PseBG33_2391	PFLU3980	PflA506_3357	PflSS101_3367
Conserved hypothetical protein	PseBG33_2419	PFLU2456	PflA506_3118	PflSS101_3127
Conserved hypothetical protein	PseBG33_2455	PFLU0566	PflA506_3079	PflSS101_3087
Transcriptional regulatory protein domain protein	PseBG33_2459	PFLU3709	PflA506_3075	PflSS101_3083
Conserved hypothetical protein	PseBG33_2526	PFLU2605	PflA506_2433	PflSS101_2988
Transketolase	PseBG33_2537	PFLU3599	PflA506_2366	PflSS101_3052
DNA-3-methyladenine glycosylase I	PseBG33_2576	PFLU3499	PflA506_2527	PflSS101_2314
Transporter, major facilitator family	PseBG33_2627	PFLU3436	PflA506_2885	PflSS101_2357
Conserved hypothetical protein	PseBG33_2812	PFLU3022	PflA506_2704	PflSS101_2629
Conserved hypothetical protein	PseBG33_2825	PFLU3034	PflA506_2691	PflSS101_2616
Conserved hypothetical protein	PseBG33_2834	PFLU3043	PflA506_2682	PflSS101_2607
Drug resistance transporter, Bcr/CflA family	PseBG33_2836	PFLU3046	PflA506_2680	PflSS101_2605
Conserved hypothetical protein	PseBG33_2892	PFLU3133	PflA506_2634	PflSS101_2574
Conserved hypothetical protein	PseBG33_2895	PFLU3135	PflA506_2630	PflSS101_2572
Ribonucleoside-diphosphate reductase, beta subunit	PseBG33_2979	PFLU2783	PflA506_2912	PflSS101_2466
PF06945 family protein	PseBG33_2981	PFLU2782	PflA506_2913	PflSS101_2464
Conserved hypothetical protein	PseBG33_3058	PFLU2699	PflA506_2997	PflSS101_2372
Conserved hypothetical protein	PseBG33_3070	PFLU2686	PflA506_3009	PflSS101_2360
Conserved hypothetical protein	PseBG33_3105	PFLU2650	PflA506_2458	PflSS101_2241
Conserved hypothetical protein	PseBG33_3184	PFLU3614	PflA506_3060	PflSS101_2966
Conserved hypothetical protein	PseBG33_3187	PFLU3616	PflA506_3063	PflSS101_2969
Translocator protein, LysE family	PseBG33_3233	PFLU3651	PflA506_2298	PflSS101_2197
Conserved hypothetical protein	PseBG33_3266	PFLU3755	PflA506_3140	PflSS101_3148
Conserved hypothetical protein	PseBG33_3342	PFLU3835	PflA506_3217	PflSS101_3223
Conserved hypothetical protein	PseBG33_3525	PFLU2409	PflA506_2280	PflSS101_2176
Putative lipoprotein	PseBG33_3552	PFLU3999	PflA506_3375	PflSS101_3385
Conserved hypothetical protein	PseBG33_3565	PFLU2297	PflA506_3382	PflSS101_3394
5'-nucleotidase, lipoprotein e(P4) family, putative	PseBG33_3609	PFLU4030	PflA506_3433	PflSS101_3444
Conserved hypothetical protein	PseBG33_3678	PFLU4166	PflA506_3475	PflSS101_3492
Pyroglutamyl-peptidase I	PseBG33_3684	PFLU4174	PflA506_3482	PflSS101_3499

doi: 10.1371/journal.pgen.1002784

Membrane protein, PF06166 family	PseBG33_3685	PFLU4175	PflA506_3483	PflSS101_3500
Membrane protein, PF06149 family	PseBG33_3686	PFLU4176	PflA506_3484	PflSS101_3501
Conserved hypothetical protein	PseBG33_3727	PFLU4215	PflA506_3528	PflSS101_3545
Conserved hypothetical protein	PseBG33_3752	PFLU4244	PflA506_3552	PflSS101_3587
Conserved hypothetical protein	PseBG33_3759	PFLU4252	PflA506_3559	PflSS101_3596
Conserved hypothetical protein	PseBG33_3821	PFLU4329	PflA506_3639	PflSS101_3658
Conserved hypothetical protein	PseBG33_3844	PFLU4357	PflA506_3662	PflSS101_3681
Putative bacteriophage-acquired protein	PseBG33_4065	PFLU4584	PflA506_3884	PflSS101_3909
Conserved hypothetical protein	PseBG33_4175	PFLU4688	PflA506_3993	PflSS101_4072
Putative outer membrane protein H1	PseBG33_4210	PFLU4721	PflA506_4028	PflSS101_4108
Conserved hypothetical protein	PseBG33_4215	PFLU4727	PflA506_4033	PflSS101_4113
Conserved hypothetical protein	PseBG33_4269	PFLU4779	PflA506_4095	PflSS101_4173
Conserved hypothetical protein	PseBG33_4643	PFLU5181	PflA506_4474	PflSS101_4538
Conserved hypothetical protein	PseBG33_4913	PFLU5430	PflA506_4721	PflSS101_4776
Conserved hypothetical protein	PseBG33_5414	PFLU5907	PflA506_5195	PflSS101_5245
Fimbrial usher protein	PseBG33_5416	PFLU5908	PflA506_5197	PflSS101_5247
Conserved hypothetical protein	PseBG33_5418	PFLU5910	PflA506_5199	PflSS101_5249
HAD-superfamily hydrolase, subfamily IA, variant 1 and 3	PseBG33_5454	PFLU5949	PflA506_5234	PflSS101_5284
Type VI secretion protein Fha1	PseBG33_5521	PFLU6017	PflA506_5299	PflSS101_5349

[a] Genes are present in genomes of the strains found in Sub-clade 3, but are not present in the genomes of other *Pseudomonas* spp. in Figure 1.

doi: 10.1371/journal.pgen.1002784
