# Supplementary material for: Comparative Genomics of Plant-Associated Pseudomonas spp.: Insights into Diversity and Inheritance of Traits Involved in Multitrophic Interactions
Source: PLoS Genet. 2012 Jul 5;8(7):e1002784. doi: 10.1371/journal.pgen.1002784 (PMC3390384; doi:10.1371/journal.pgen.1002784)
Supplement: Table S10 — Consensus sequences and logos of REP elements in the genomes of the P. fluorescens group. HMM searches were used to identify the occurrence of REP elements within the genomes of strains in the P. fluorescens group. The number of occurrences as well as the consensus sequence and consensus sequence logo are presented for REP elements appearing more than 250 times in a genome. Imperfect palindromes identified within the consensus sequence are highlighted in red and blue and palindromic nucleotides are underlined. (PDF) [file pgen.1002784.s020.pdf]

Table S10. REP elements appearing in *Pseudomonas* genomes more than 250 times.

Strain	REP	HMM hits[a]	Consensus[b,c]	Logo[d]
Pf-5	REPa	999	CCCGTAGGAGCCGGCTTGCCGGCGAAGAGGCCCGCAAGCC	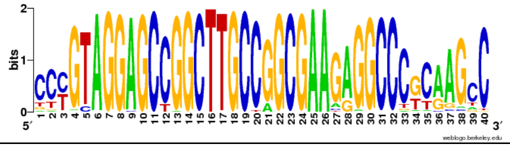
30-84	REPa	620	CCCTGTAGGAGCGAGCTTGCTCGCGATAGCGT	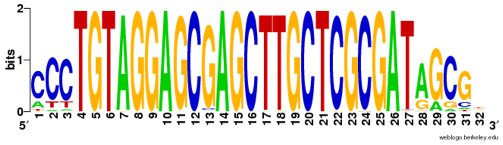
	REPb	801	GTCGATCGCAGCCTCGCGGGCTCGGCAGCGGCTACAGGG	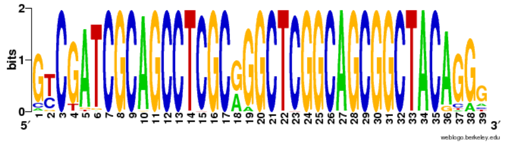
O6	REPa	888	ATCGCGGGCAAGCCTCGCTCCTACAGAAGC	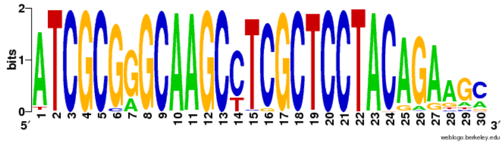
	REPb	676	CGATCGCAGCCTCGCAGGCTCGGCAGCGGCTACAGGG	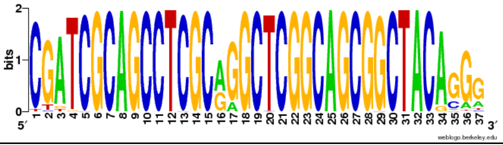
Q8r1-96	REPa	1987	ATCGCGAGCAAGCTTTGCTCCCACAGGTCCT	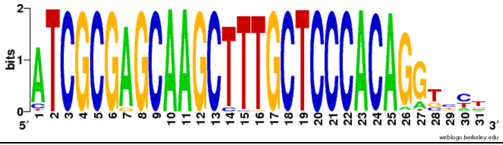
Q2-87	REPa	1276	TTCGCGAGCAAGCCCGCTCCCACAGGGGATTTT	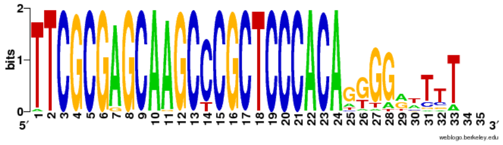
	REPd	774	TGGGGCTGCTGCGCAGCCCAGCGGGAGCAAGCTCCCTCGCCACA	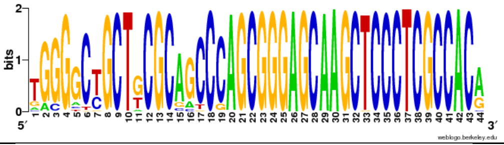
SBW25[e]	REPa	1024	TGTGGGAGGGGGCTTGCCCCCGATGGCG	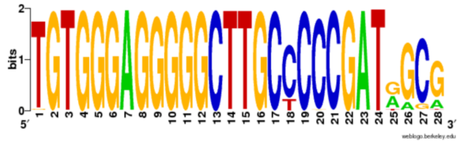
	REPd	981	CGGGAGCAAGCTCCCTCGCCACA	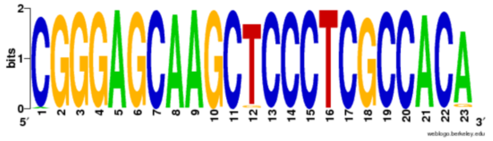
	REPe	325	CCCAGCGCGGGGCAAGCCCGCTCAC	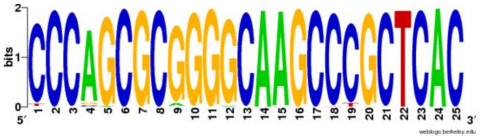

doi: 10.1371/journal.pgen.1002784

BG33R	REPa	1119	ATCGGGAGCAAGCCCCCTCCCACATT	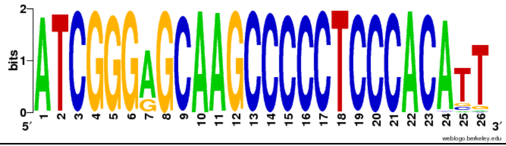
A506	REPa	1397	TGTGGGAGCTGGCTTGCCTGCGATAGCGG	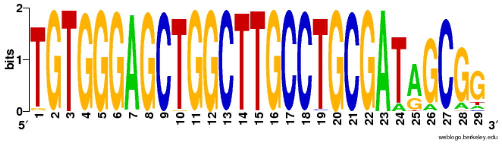
	REPc	255	GCATTCCCACGCAGAGCGTGGGAACGATCA	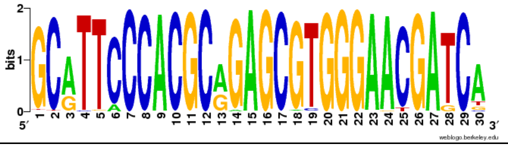
SS101	REPa	1559	ACCGCCATCGCAGGCAAGCCAGCTCCCACA	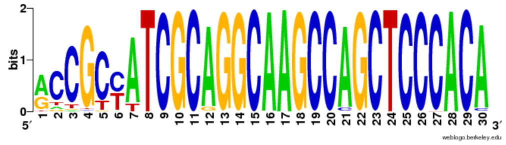
	REPc	270	GCGGCATTCCCACGCAGAGCGTGGGAACGATCA	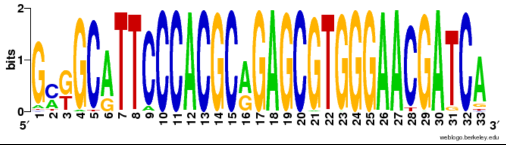

[a] Number of HMM hits to the respective genome using an E value cut-off of 10^{-3}
[b] Consensus of sequences used to generate HMM
[c] Palindromic sequences are indicated by red/blue shading and are underlined
[d] Logos were generated from the alignments used to generate HMMs
[e] Some overlap was observed between hits from SBW25 HMMs using an E value cut off of 10^{-3}

doi: 10.1371/journal.pgen.1002784
