# Supplementary material for: Comparative Genomics of Plant-Associated Pseudomonas spp.: Insights into Diversity and Inheritance of Traits Involved in Multitrophic Interactions
Source: PLoS Genet. 2012 Jul 5;8(7):e1002784. doi: 10.1371/journal.pgen.1002784 (PMC3390384; doi:10.1371/journal.pgen.1002784)
Supplement: Table S11 — REP HMM hits across Pseudomonas spp. genome sequences. HMM searches across the genomes of a collection of Pseudomonas strains were conducted using the REP sequences identified within the P. fluorescens group to gauge the broader distribution of these sequence elements. Shading highlights strains containing large numbers of REP sequence elements: REPa, grey; REPb, pink; REPc, green; REPd, orange; REPe, blue. (PDF) [file pgen.1002784.s021.pdf]

Table S11. REP HMM hits across genome sequences of *Pseudomonas* spp.

	Number of HMM hits using E value limit of 10^{-3} [a]																											
	P. fluorescens group										*P. syringae*					*P. entomophila*	*P. putida*				*P. aeruginosa*					Other species[c]			
	**Pf-5**	**30-84**	**O6**	**Pf0-1**	**Q8r1-96**	**Q2-87**	**BG33R**	**SBW25**	**A506**	**SS101**	**DC3000**	**T1**	**1448A**	**NCPPB3681**	**B728a**	**L48**	**KT2440**	**F1**	**GB-1**	**W619**	**PAO1**	**PA7**	**PA14**	**LESB58**	**PACS2**	**ymp**	**A1501**	**DJ**	**MG1655**
**REPa (primary REP sequence)**																													
Pf-5 REPa	999	106	149	56	153	272	128	38	348	403	6	7	27	28	81	1019	457	544	109	309	2	2	3	2	2	32	6	4	0
30-84 REPa	623	620	876	169	1931	1409	911	781	1329	1509	37	39	108	88	352	868	483	658	357	333	4	3	2	3	3	43	9	11	0
O6 REPa	545	601	888	66	1794	905	202	62	428	733	15	16	42	39	175	1042	318	407	65	159	2	3	2	2	2	34	11	6	1
Q8r1-96 REPa	49	487	785	110	1987	1127	251	71	578	810	13	12	48	42	128	242	135	154	54	152	1	3	1	1	1	7	1	2	0
Q2-87 REPa	470	456	611	172	1776	1276	965	729	1394	1586	54	51	100	77	257	685	685	980	302	244	1	0	0	1	1	13	3	7	0
BG33r REPa	252	397	516	134	1574	1807	1119	1140	1419	1613	18	18	40	46	118	421	474	642	182	253	0	1	0	0	0	23	6	4	0
SBW25 REPa[b]	45	260	348	123	1581	1468	1017	1024	1308	1113	25	23	48	46	110	52	295	360	125	195	0	0	0	0	0	3	1	2	0
A506 REPa	763	303	423	140	1582	1175	879	718	1397	1350	27	22	51	44	94	558	412	523	171	315	0	1	1	0	0	13	2	3	0
SS101 REPa	823	367	547	136	1666	1190	932	738	1434	1559	40	34	83	64	154	571	518	719	183	309	0	0	0	0	0	17	4	4	1
**REPb**																													
30-84 REPb	186	801	683	0	13	1	3	14	16	6	0	0	0	1	2	2	1	3	0	1	3	1	3	3	3	1	1	3	0
O6 REPb	185	804	676	1	27	1	4	21	18	7	0	0	0	0	1	1	0	1	0	2	1	1	1	1	1	0	1	2	0
**REPc**																													
A506 REPc	0	1	1	140	213	0	3	5	255	291	183	167	341	92	356	0	1	0	1	1	0	0	1	0	0	5	0	4	0
SS101 REPc	0	0	0	114	203	0	4	3	243	270	170	159	331	68	342	0	1	0	1	1	0	0	0	0	0	4	0	2	0
**REPd**																													
Q2-87 REPd	22	8	7	2	101	774	32	514	35	25	0	1	0	0	0	3	0	5	0	1	0	0	0	0	0	0	0	0	0
SBW25 REPd[b]	40	10	5	28	883	1005	476	981	88	77	8	7	27	25	94	3	3	4	2	2	0	0	0	1	0	0	3	2	0
**REPe**																													
SBW25 REPe[b]	5	6	6	11	14	11	9	325	36	28	1	2	5	6	6	54	11	21	6	9	9	17	12	11	10	6	4	6	0

Color codes: Grey, genomes having ≥ 100 REPa sequences; pink, genomes having ≥ 100 REPb sequences; green, genomes having ≥ 100 REPc sequences; dark orange, genomes having ≥ 100 REPd sequences; gold, genomes having ≥ 30 but <100 REPd sequences; blue, genomes having ≥ 100 REPe sequences.

a. Using an E value cut off of 10^{-3} we observed very little overlap between HMM hits of secondary REP HMMs with those of the primary REPa HMM from each strain. Refer to Figure 1 for species designations of strains.

b. Using an E value cut off of 10^{-3} we observed a degree of overlap between SBW25 REPa, REPd and REPe HMM hits. However, using a stringency of 10^{-7} this overlap is negligable.

c. *Pseudomonas mendocina* strain ymp, *Pseudomonas stutzeri* A1501, *Azotobacteria vinelandii* DJ, and *Escherichia coli* MG1655

doi: 10.1371/journal.pgen.1002784
