# Supplementary material for: Comparative Genomics of Plant-Associated Pseudomonas spp.: Insights into Diversity and Inheritance of Traits Involved in Multitrophic Interactions
Source: PLoS Genet. 2012 Jul 5;8(7):e1002784. doi: 10.1371/journal.pgen.1002784 (PMC3390384; doi:10.1371/journal.pgen.1002784)
Supplement: Table S12 — Transposons present in the genomes of seven strains in the P. fluorescens group. The following information is provided for each putative transposon in the genomes of strains 30-84, O6, Q8r1-96, Q2-87, BG33R, A506, and SS101: transposon family, transposases, and numbers of copies of intact or remnant transposons in each genome. (PDF) [file pgen.1002784.s022.pdf]

Table S12. Transposons present in sequenced *Pseudomonas fluorescens* group genomes[a]

Type[b]	Family/ Group	Transposase	Copies	№ of intact copies (locus tags)	№ of remnants (locus tags)
**Strain 30-84**					
ISPpu8-like	IS4/IS4	1 ORF	1	1 (Pchl3084_2929)	
ISBph1-like	IS3	3 ORFs	1		1 (Pchl3084_2928)
IS222-like	IS3	3 ORFs	1		1 (Pchl3084_2959)
ISPsy10-like	IS5	1 ORF	1		1 (Pchl3084_2968)
ISPsy24-like	IS3	3 ORFs	1		1 (Pchl3084_3486)
ISAzvi6-like	IS4	1 ORF	1		1 (Pchl3084_5080)
**Strain O6**					
ISPGme5-like	ISL3	1 ORF	12	11 (PchlO6_0389; PchlO6_1202; PchlO6_1401; PchlO6_1492; PchlO6_1888; PchlO6_1948; PchlO6_1955; PchlO6_4536; PchlO6_4620; PchlO6_5453; PchlO6_6080)	1 (PchlO6_4883 and PchlO6_4884, frameshift)
ISRso10-like	IS3/IS2	2 ORFs	2	2 (PchlO6_3587 – PchlO6_3588; PchlO6_3594 – PchlO6_3595)	
ISPsy12-like	IS3	2 ORFs	1		1 (PchlO6_6073, contains frameshift)
ISXc5-like	Tn3	2 ORFs	2		2 (PchlO6_6050; PchlO6_6087 and PchlO6_6088; both contain frameshifts)
ISPst8-like	IS5	3 ORFs	2		2 (PchlO6_6086; PchlO6_6112; both contain frameshifts)
ISPsy2-like	IS5	1 ORF	1		1 (PchlO6_6093, contains frameshift)
**Strain Q8r1-96**					
ISPpu10-like	IS110	1 ORF	6	5 (PflQ8_4357; PflQ8_1705; PflQ8_3208; PflQ8_5066; PflQ8_5327)	1 (PflQ8_1368 and PflQ8_1369, frameshift)
ISPpu14-like	IS66	3 ORFs	1	1 (PflQ8_3883 – PflQ8_3885)	
IS200-like	IS66	1 ORF	2	2 (PflQ8_1467; PflQ8_1468)	
ISPst8-like	IS5	3 ORFs	1		1 (PflQ8_3542, truncated)
ISAzvi11-like	IS481	1 ORF	1		1 (PflQ8_0849, truncated)
**Strain Q2-87**					
ISPpu14-like	IS66	3 ORFs	4	4 (PflQ2_4627 – PflQ2_4629; PflQ2_1598 – PflQ2_1600; PflQ2_0680 – PflQ2_0681, and PflQ2_0683; PflQ2_5393 – PflQ2_5395)	
ISPen1-like	IS4	1 ORF	1	1 (PF03_0821)	
ISPpu10-like	IS110	1 ORF	1	1 (PF03_3914)	
ISPpu13-like	IS66	3 ORFs	1		1 (PflQ2_4000)
Tn2501-like	Tn3	2 ORFs	1		1 (PflQ2_4075)
ISPsy24-like	IS3	1 ORF	2		2 (PflQ2_3275; PflQ2_1994 and PflQ2_1995, frameshift)
SPpu9-like	IS110	1 ORF	1		1 (PflQ2_1621)

doi: 10.1371/journal.pgen.1002784

**Strain BG33R**					
ISPst10-like	IS30	1 ORF	2	1 (PseBG33_2173)	1 (PseBG33_2211 and PseBG33_2212, frameshift)
ISPsy29-like	IS3/IS51	3 ORFs	1	1 (PseBG33_2176 – PseBG33_2178)	
ISPsy5-like	IS66	3 ORFs	1	1 (PseBG33_2198 – PseBG33_2200)	
ISPen1-like	IS4/IS4	2 ORFs	2	2 (PseBG33_2209 – PseBG33_2210; PseBG33_4397 – PseBG33_4398)	
ISPa7-like	IS1182	1 ORF	2		2 (PseBG33_3744; PseBG33_4768 and PseBG33_4769, frameshift)
ISPst8-like	IS5/IS427	3 ORFs	1		1 (PseBG33_2196 – PseBG33_2197)
ISPpu14-like	IS66	3 ORFs	1		1 (PseBG33_2206)
ISPsy2-like	IS5/IS5	1 ORF	1		1 (PseBG33_2217)
ISPst12-like	IS5	1 ORF	1		1 (PseBG33_5153 and PseBG33_5154, frameshift)
**Strain A506**					
IS222-like	IS3/IS3	2 ORFs	7	6 (PflA506_2142 – PflA506_2143; PflA506_2258 – PflA506_2259; PflA506_2368 – PflA506_2369; PflA506_2631 – PflA506_2632; PflA506_3053 – PflA506_3054; PflA506_3570 – PflA506_3571)	1 (PflA506_5313 and PflA506_5315, truncated)
ISPpu8-like	IS4/IS4	1 ORF	9	9 (PflA506_2272; PflA506_2345; PflA506_2982; PflA506_5181; PflA506_0615; PflA506_2099; PflA506_2243; PflA506_2370; PflA506_2624)	
ISPpu14-like	IS66	3 ORFs	1	1 (PflA506_1651, PflA506_1652, PflA506_1653)	
ISPpu13-like	IS66	3 ORFs	2		2 (PflA506_2865, contains frameshift; PflA506_1477[c], truncated)
**Strain SS101**					
ISPpu14-like	IS66	3 ORFs	2	1 (PflSS101_1463 – PflSS101_1465)	1 (PflSS101_4026 – PflSS101_4027)
ISPen1-like	IS4	1 ORF	1		1 (PflSS101_2810, truncated)
ISPpu13-like	IS66	3 ORFs	2		2 (PflSS101_4020 – PflSS101_4021; PflSS101_2886)
ISCfr1-like	IS1182	1 ORF	1		1 (PflSS101_4033, frameshift)

[a] Strains Pf-5, Pf0-1, and SBW25 were not considered in this analysis
[b] Only expectation values of 1e-05 and below were considered as significant matches during BLAST database searches.
[c] Gene not present in the genome annotation (called gene is in incorrect reading frame).

doi: 10.1371/journal.pgen.1002784
