# Supplementary material for: Comparative Genomics of Plant-Associated Pseudomonas spp.: Insights into Diversity and Inheritance of Traits Involved in Multitrophic Interactions
Source: PLoS Genet. 2012 Jul 5;8(7):e1002784. doi: 10.1371/journal.pgen.1002784 (PMC3390384; doi:10.1371/journal.pgen.1002784)
Supplement: Table S13 — Mobile genetic elements in the genomes of seven strains in the P. fluorescens group. The following information is provided for each prophage or genomic island in the genomes of strains 30-84, O6, Q8r1-96, Q2-87, BG33R, A506, and SS101: presence of an integrase, insertion site, size, locus tags, and selected cargo genes. (PDF) [file pgen.1002784.s023.pdf]

Table S13. Mobile genetic elements in the genomes of the *Pseudomonas fluorescens* group[a]

MGE type	Integrase	Insertion site	Size (kb)	Start locus tag	Stop locus tag	Cargo genes and predicted functions
**Strain 30-84**						
prophage 1	none	*mutS-cinA*	33.8	Pchl3084_1193	Pchl3084_1232	Bacteriocin, LlpA family; gene blocks I, II, and IV
prophage 2[b]	not intact	tRNA-Gly	3.4	Pchl3084_1391	Pchl3084_1393	Pilus assembly chaperone CupC2; flanked by *attL* and *attR*
prophage 3[b]	not intact	tRNA-Val	5.2	Pchl3084_2012	Pchl3084_2016	Acyltransferase; flanked by *attL* and *attR*
island 1	intact	tmRNA ssrA	14.6	Pchl3084_0791	Pchl3084_0800	Type I restriction modification system
island 2	intact		74.1	Pchl3084_2928	Pchl3084_2967	Toxin complex (Tc) proteins; Mu-like transposase; ISPpu8-like transposase
island 3	intact	tRNA-Leu	23.0	Pchl3084_4769	Pchl3084_4791	Polyphosphate kinase; two-component signal-transduction system
**Strain O6**						
prophage 1	intact	*mutS-cinA*	72.3	PchlO6_1205	PchlO6_1304	DNA helicase, phosphatase family protein; transcriptional regulators; gene blocks I and II
prophage 2	intact	tRNA-Ser	36.5	PchlO6_1500	PchlO6_1543	N6 adenine-specific DNA methyltransferase; bifunctional DNA primase/polymerase
prophage 3	intact	tRNA dihydrouridine synthase A	56.9	PchlO6_2092	PchlO6_2179	C-5 cytosine-specific DNA methylase family protein
island 1[b]	intact	tRNA-Leu	41.6	PchlO6_3551	PchlO6_3585	Glutathione S-transferase 2; TonB-dependent siderophore receptor; molybdopterin-binding oxidoreductase; heavy metal two-component signal-transduction system; MFS transporter
island 2	intact	tRNA-Leu	23.6	PchlO6_5017	PchlO6_5042	Transcriptional regulators; nucleoid-associated protein; polyphosphate kinase; two-component signal-transduction system
island 3	intact	putative Mg chelatase	125.1	PchlO6_6034	PchlO6_6114	Mevalonate-independent pathway for isoprenoid production; biofilm associated proteins

doi: 10.1371/journal.pgen.1002784

**Strain Q8r1-96**						
prophage 1	none	*mutS-cinA*	22.0	PflQ8_1124	PflQ8_1150	Bacteriocin, colicin M family; gene block II
prophage 2	intact	tRNA dihydrouridine synthase A	37.7	PflQ8_1855	PflQ8_1897	Ultraviolet light resistance proteins RulAB; C-5 cytosine-specific DNA methylase
island 1	intact	tmRNA ssrA	16.5	PflQ8_0767	PflQ8_0776	Type II restriction enzyme; putative nuclease
island 2	not intact	tRNA-Gly	62.2	PflQ8_2129	PflQ8_2160	Catalase; UvrD/REP helicase family protein
island 3b	intact	tRNA-Pro	7.1	PflQ8_3770	PflQ8_3779	C-5 cytosine-specific DNA methylase; N-4 cytosine methyltransferase
island 4	intact	tRNA-Met	16.9	PflQ8_4877	PflQ8_4889	Reverse transcriptase-like protein; transcriptional regulator
transposon	none	oxidoreductase	5.2	PflQ8_3538	PflQ8_3542	Catabolism of herbicide bromoxynil flanked by IS5 elements; transcriptional regulator; cation:acetate symporter, ActP family; hydrolase, carbon-nitrogen family
**Strain Q2-87**						
prophage 1	none	*mutS-cinA*	17.0	PflQ2_4322	PflQ2_4342	Conserved hypothetical proteins; gene block I
prophage 2	intact	tRNA dihydrouridine synthase A	48.2	PflQ2_3538	PflQ2_3584	N6 adenine-specific DNA methyltransferases; ultraviolet light resistance proteins RulAB
island 1	intact	tRNA-Thr	11.8	PflQ2_5389	PflQ2_5396	DEAD/DEAH box helicase-like protein; ISPpu14-like transposon
island 2	intact	tRNA-Met	20.5	PflQ2_4617	PflQ2_4632	ISPpu14-like transposon; transcriptional regulator
island 3[b]	intact	tRNA-Gly	15.5	PflQ2_3275	PflQ2_3289	ISPsy24-like transposon (inactive)
island 4	not intact	tRNA-Val	9.4	PflQ2_1752	PflQ2_1766	Transcriptional regulator; integral membrane protein
island 5[b]	intact	tRNA-Met	9.4	PflQ2_0575	PflQ2_0586	Plasmid conjugation machinery
**Strain BG33R**						
prophage 1	intact	*mutS-cinA*	64.0	PseBG33_1169	PseBG33_1255	Phosphatase family protein; transcriptional regulators; endonuclease; gene blocks I, III, and V

doi: 10.1371/journal.pgen.1002784

prophage 2	intact		50.7	PseBG33_1320	PseBG33_1392	C-5 cytosine-specific DNA methylase; flanked by *attL* and *attR*
prophage 3	Intact	tRNA dihydrouridine synthase A	12.7	PseBG33_1691	PseBG33_1701	Conserved hypothetical proteins
prophage 4	intact		37.2	PseBG33_3468	PseBG33_3517	D12 class N6 adenine-specific DNA methyltransferase; transcriptional regulators; integrase domain protein
island 1	intact		20.5	PseBG33_0005	PseBG33_0024	Reverse transcriptase-like protein; N-6 DNA methylase family protein; plasmid partitioning
island 2	intact	tRNA-Arg	16.6	PseBG33_0201	PseBG33_0214	Conserved hypothetical proteins
island 3[c]	intact	tRNA-Gly	154.3	PseBG33_2111	PseBG33_2266	IAA uptake and catabolism; MexCD-like MDR transporter; pertussis toxin subunit-like protein; haloaromatics catabolism; plasmid replication, partitioning and conjugation; transposons; TonB-dependent receptors; *attL*
island 4	intact		2.3	PseBG33_2835	PseBG33_2837	Transporter, Bcr/CflA subfamily
island 5	not intact	tRNA-Pro	6.5	PseBG33_3641	PseBG33_3651	Phage repressor; kinase domain protein (zeta toxin)
island 6	intact	SRP RNA[d]	11.3	PseBG33_4068	PseBG33_4075	Resolvase domain protein; reverse transcriptase
island 7[e]	intact	tmRNA	3.1	PseBG33_4754	PseBG33_4757	Transcriptional regulator
**Strain A506**						
prophage 1	none	*mutS-cinA*	19.5	PflA506_1133	PflA506_1160	D-isomer specific 2-hydroxyacid dehydrogenase; dimethylmenaquinone methyltransferase; ultraviolet light resistance protein; gene block I
prophage 2	intact	tRNA dihydrouridine synthase A	35.0	PflA506_1613	PflA506_1663	D12 class N6 adenine-specific DNA methyltransferase; DNA-cytosine methyltransferase
prophage 3	intact	alpha/beta superfamily hydrolase	52.5	PflA506_2008	PflA506_2084	Bacteriocin, LlpA family; reverse transcriptase domain protein

doi: 10.1371/journal.pgen.1002784

island 1	intact	tRNA-Ser	75.3	PflA506_1413	PflA506_1490	Bacteriocin, Pyocin S5 family; adenine-specific modification methylase; DNA ligase; flanked by *attL* and *attR*
island 2	intact		20.9	PflA506_2668	PflA506_2677	Hypothetical proteins; flanked by direct repeats
island 3	intact	tmRNA ssrA	20.3	PflA506_4578	PflA506_4587	Helicase domain protein; DNA methylase N-4/N-6 domain protein; flanked by direct repeats
plasmid	intact		57.0	PflA506_p0001	PflA506_p0066	Conserved hypothetical proteins, lesion bypass DNA polymerase
**Strain SS101**						
prophage 1	none	*mutS-cinA*	19.8	PflSS101_1171	PflSS101_1199	N-acetylmuramoyl-L-alanine amidase domain protein; gene block I
island 1	intact	tRNA-Arg	16.6	PflSS101_0186	PflSS101_0197	Conserved hypothetical proteins
island 2[c]	intact	tRNA-Ser	20.5	PflSS101_1457	PflSS101_1490	Thiol:disulfide interchange protein; cyclic diguanylate phosphodiesterase domain proteins; sensory box His kinases/response regulators; DNA-binding response regulators; cluster of fimbrial proteins; ISPpu14-like transposon; flanked by *attL* and *attR*
island 3	intact		8.5	PflSS101_2599	PflSS101_2606	Methyl-accepting chemotaxis protein; transporter, Bcr/CflA subfamily; conserved hypothetical proteins
island 4	intact	tRNA-Leu	86.1	PflSS101_2702	PflSS101_2799	Relaxase; nucleases; ultraviolet light resistance proteins RulAB; beta-lactamase; transcriptional regulators; restriction endonuclease; Ser/Thr phosphatase family protein
island 5	intact	SRP RNA[c]	12.9	PflSS101_3912	PflSS101_3925	Resolvase domain protein
island 6	intact	tRNA-Ser	61.0	PflSS101_4000	PflSS101_4040	Fimbrial biogenesis; two-component signal-transduction system; transcriptional regulators; type I secretion transporter system; surface adhesins/hemagglutinins; topoisomerase DNA-binding C4 zinc finger domain protein; transposons; flanked by *attL* and *attR*

doi: 10.1371/journal.pgen.1002784

island 7	intact	tmRNA	14.5	PflSS101_4646	PflSS101_4655	DEAD/DEAH box helicase-like protein

[a] Strains Pf-5, Pf0-1, and SBW25 were not considered in this analysis; [b] Prophage remnant; for islands, part of the island is a prophage remnant; [c] Integrative conjugative element; [d] Signal recognition particle RNA (SRP RNA); [e] Island remnant

doi: 10.1371/journal.pgen.1002784
