# Supplementary material for: Comparative Genomics of Plant-Associated Pseudomonas spp.: Insights into Diversity and Inheritance of Traits Involved in Multitrophic Interactions
Source: PLoS Genet. 2012 Jul 5;8(7):e1002784. doi: 10.1371/journal.pgen.1002784 (PMC3390384; doi:10.1371/journal.pgen.1002784)
Supplement: Table S14 — Bioassays linking gene inventories to phenotypes of strains in the P. fluorescens group. Ten strains were evaluated for the production of levan sucrase, exoprotease, gelatinase, lipase, chitinase, and hydrogen cyanide as well as biosurfactant and hemolytic activities associated with cyclic lipopetide production. Derivatives of some strains having mutations in ofaA, aprA, hcnB, viscA, massA, or gacA were also evaluated to serve as negative controls in these experiments correlating genotypes to phenotypes. A derivative of Pf0-1 containing a plasmid-borne gacA + produced exoprotease, gelatinase, lipase, chitinase, and hydrogen cyanide and exhibited biosurfactant and hemolytic activity. In contrast, strain Pf0-1 was negative for these phenotypes, supporting our conclusion that the sequenced strain of Pf0-1 has a mutation in gacA. (PDF) [file pgen.1002784.s024.pdf]

Table S14. Bioassays evaluating exoenyzme and cyclic lipopeptide production by strains in the *Pseudomonas fluorescens* group and derivatives[a]

	Pf-5	Pf-5 *gacA*	Pf-5 *ofaA*	Pf-5 *aprA*	Pf-5 *chiC*	Pf-5 *hcnB*	30-84	O6	Pf0-1	Pf0-1 *gacA+*	Pf0-1 *gacS+*	Q8r1-96	Q2-87	SBW25	SBW25 *viscA*	A506	A506 *rpoS+*	A506 *gacS*	A506 *aprA*	SS101	SS101 *massA*	BG33R
Levan sucrase	-	-	nt	nt	nt	nt	+	+	-	-	nt	-	+	+	nt	+	nt	nt	nt	+	nt	-
Exoprotease	+	-	nt	-	+	+	+	+	-	+	-	+	w	+	nt	+	nt	-	-	+	nt	+
Gelatinase	+	-	nt	w	nt	nt	+	+	-	+	nt	+	+	+	nt	+	nt	w	-	+	nt	+
Lipase	+	+	nt	nt	nt	nt	+	+	+	+	nt	+	+	-	nt	+	nt	+	nt	+	nt	+
Chitinase[b]:																						
4-Methylumbelliferyl N,N'-diacetyl-β-D-chitobioside	1920 ±30	14.9± 1.9	nt	nt	9.5 ±0.0	nt	2400 ±40	2250 ±40	13.2 ±1.7	1710 ±10	nt	4.8 ±0.0	4.8 ±3.4	15.6 ±1.7	nt	390 ±8	491 ±8	nt	nt	465 ±20	nt	778 ±7
4-Methylumbelliferyl N-acetyl-β-D-glucosaminide	13.2 ±1.7	10.8± 0.0	nt	nt	7.6 ±2.7	nt	2.4 ±0.0	8.4 ±1.7	10.8 ±1.7	9.6 ±0.0	nt	4.8 ±0.0	4.8 ±0.0	14.4 ±0.0	nt	14.6 ±1.9	19.9 ±1.9	nt	nt	9.6 ±0.0	nt	12.0 ±3.4
4-Methylumbelliferyl β-D-N,N',N"-triacetylchitotriose	799 ±2	12.2± 1.9	nt	nt	9.5 ±0.0	nt	1270 ±10	1340 ±20	10.8 ±1.7	433 ±0	nt	6.0 ±1.7	4.8 ±0.0	14.4 ±0.0	nt	99.5 ±1.9	125 ±0.0	nt	nt	123 ±2	nt	206 ±3
Cyclic lipopeptide:																						
Hemolysis	+	-	-	nt	nt	nt	-	-	-	+	nt	-	-	+	-	-	-	nt	nt	+	-	+
Droplet collapse	+	-	-	nt	nt	nt	-	-	-	+	-	-	-	+	-	-		-	nt	+	-	+
Hydrogen cyanide	+	-	nt	nt	+	-	+	+	-	+	-	+	+	-	nt	-	nt	-	nt	-	nt	-

Abbreviations: +, positive; -, negative; w, weak positive; nt, not tested.

[a]Derivatives of strains described previously: The *gacA* and *ofaA* (orfamide A biosynthesis) mutants of Pf-5 [1], viscosin-deficient mutant (*viscA*) of strain SBW25 [2], massetolide-deficient mutant (*massA*) of strain SS101 [3], *gacS* mutant of A506 [4], *aprA* (for extracellular alkaline protease, previously called *aprX*) mutant of A506 [4], and an *rpoS*[+] derivative of A506 [5]. Mutants of Pf-5 having deletions in *aprA* (for extracellular alkaline protease), *chiC* (for chitinase), and *hcnB* (for hydrogen cyanide biosynthesis) were constructed as described in the Materials and Methods. The *gacA*[+] derivative of Pf0-1 harbors plasmid pJEL5965 (containing the *gacA*[+] gene of strain Pf-5) and the *gacS*[+] derivative of Pf0-1 harbors plasmid pJEL5999 (containing the *gacS*[+] gene of strain Pf-5).

[b]Chitinase activity was estimated from the amount of 4-Methylumbelliferone (ng) released, calculated from a standard curve generated with 4-Methylumbelliferone controls provided in chitinase assay kit (Sigma, St. Louis, MO, USA).

References:

1. Hassan KA, Johnson A, Shaffer BT, Ren Q, Kidarsa TA, et al. (2010) Inactivation of the GacA response regulator in *Pseudomonas fluorescens* Pf-5 has far-reaching transcriptomic consequences. Environ Microbiol 12: 899-915.
2. de Bruijn I, de Kock MJD, Yang M, de Waard P, van Beek TA, et al. (2007) Genome-based discovery, structure prediction and functional analysis of cyclic lipopeptide antibiotics in *Pseudomonas* species. Mol Microbiol 63: 417-428.
3. de Bruijn I, de Kock MJ, de Waard P, van Beek TA, Raaijmakers JM (2008) Massetolide A biosynthesis in *Pseudomonas fluorescens*. J Bacteriol 190: 2777-2789.
4. Anderson LM, Stockwell VO, Loper JE (2004) An extracellular protease of *Pseudomonas fluorescens* inactivates antibiotics of *Pantoea agglomerans*. Phytopathology 94: 1228-1234.
5. Hagen MJ, Stockwell VO, Whistler CA, Johnson KB, Loper JE (2009) Stress tolerance and environmental fitness of *Pseudomonas fluorescens* A506, which has a mutation in RpoS. Phytopathology 99: 679-688.

doi: 10.1371/journal.pgen.1002784
