# Supplementary material for: Comparative Genomics of Plant-Associated Pseudomonas spp.: Insights into Diversity and Inheritance of Traits Involved in Multitrophic Interactions
Source: PLoS Genet. 2012 Jul 5;8(7):e1002784. doi: 10.1371/journal.pgen.1002784 (PMC3390384; doi:10.1371/journal.pgen.1002784)
Supplement: Table S15 — Putative type III secretion system effectors were identified in six genomes of the P. fluorescens group. T3SS effectors were identified by BLASTp, based on their similarities to members of known bacterial effector families. The six genomes also were screened using hidden Markov models (HMMs) built from the compilation of P. syringae Hrp boxes. Putative T3SS effector genes were identified in the Q8r1-96, A506, SS101 and BG33R genomes based on the presence of possible Hrp boxes and N-termini typical of T3SS-secreted proteins (i.e., abundance of Ser and polar residues at the N-termini, only one acidic residue in the first 12 positions, and an aliphatic amino acid in position 3 or 4). The following information is provided for each putative T3SS effector: Locus tag, gene name, %G+C, sequence of the putative Hrp box, amino acid residues in the N-terminus, and closest protein match. (PDF) [file pgen.1002784.s025.pdf]

Table S15. Putative type III secretion system effectors in genomes of the *Pseudomonas fluorescens* group[a]

Locus tag	Gene name	RBS[b]	%G+C	Hrp box			Length (AAs)	N-terminal residues				Overall closest protein match [Organism]; BLASTp e-value, accession number
				Present?	Position	Sequence		Serines	Acidic	Polar	Aliphatic	
**Strain Q8r1-96**												
PflQ8_1079	*ropAA*	AAGAG	63	Yes	-42	c**ggaacc**gataggaaagaggtgg**ccac**aca	451	5	1	27	Yes	Putative type III effector protein *Ralstonia solanacearum* MolK2; e-value $1e^{-62}$ YP_002255352
PflQ8_5454		GAGG	63	Yes	-72	g**ggaacg**atcattagccttgctt**ccac**agc	205	1	1	10	No	Putative haemolysin Pfl01_5292 *Pseudomonas fluorescens* Pf0-1; e-value $6e^{-105}$ ABA77029
PflQ8_5542	*ropB*	AGGA	62	Yes	-81	t**ggaacc**gatgcgcacgcgcttg**ccac**tca	167	8	1	29	No	Putative type III effector protein RopB from *Pseudomonas fluorescens* Q2-87; e-value $3e^{-78}$
PflQ8_5560	*ropM*	GGAG	60	No*[c]			705	7	1	26	Yes	HopPtoM-like protein from *Pseudomonas viridiflava* PNA3.3a (AAT96220); $9e^{-20}$
**Strain Q2-87**												
PflQ2_4389	*ropAA*	AAGAG	62	Yes	-42	c**ggaacc**gataggcaagaggtga**ccac**aca	451	6	1	28	Yes	Putative type III effector protein *Ralstonia solanacearum* UW551; e-value $7e^{-63}$ ZP_00944509
PflQ2_0323	*ropM*	GGAG	59	No*[c]			708	6	1	25	No	HopPtoM-like protein from *Pseudomonas viridiflava* PNA3.3a; e-value $2e^{-21}$ AAT96220
PflQ2_0341	*ropB*	GGAG	62	Yes	-81	t**ggaacc**gatgcacatgcggttg**ccac**tca	172	9	1	28	No	Putative type III effector protein RopB from *Pseudomonas fluorescens* Q8r1-96; e-value $3e^{-78}$
PflQ2_0447	*exoU*	GGAAC	57	No			646	4	1	24	Yes	Hypothetical protein PFLU5718 [*Pseudomonas fluorescens* SBW25];e-value $2e^{-109}$ YP_002875210
**Strain BG33R**												
PseBG33_0857		GTGAG	53	Yes	-148	t**ggaact**tacggcactcaacttt**ccac**cca	932	4	0	29	Yes	Hypothetical protein EBI_24284 [*Enterocytozoon bieneusi* H348]; e-value $4e^{-93}$ EED41738
PseBG33_1291		GTGA	58	Yes	-63	g**ggaacc**gttttgtgtaaaaaag**ccac**aga	52	2	1	27	Yes	Hypothetical protein
PseBG33_1500		AAGGAA	53	Yes	-76	t**ggaact**cagtactccttcccgc**ccac**tat	377	6	0	32	No	Hypothetical protein Snas_0263 [*Stackebrandtia nassauensis* DSM

												44728]; e-value $2e^{-04}$ YP_003509074
PseBG33_1516		GGAG	56	Yes	-48	t**ggaacc**tgattagcgttcgga**ccac**aaa	285	7	0	23	Yes	Hypothetical protein PFLU4727 [*Pseudomonas fluorescens* SBW25];e-value $4e^{-37}$ YP_002874248
PseBG33_1624		AGGA	57	Yes	-49	g**ggaacc**ttcttctctgatttt**ccac**cca	1490	4	0	21	Yes	Hypothetical protein PFLU3022 [*Pseudomonas fluorescens* SBW25];e-value $6e^{-85}$ YP_002872601
PseBG33_2028		GGAG	57	Yes	-75	a**ggaact**gtttccgacacaaaa**ccac**aca	329	8	1	27	Yes	Hypothetical protein PFLU2099 [*Pseudomonas fluorescens* SBW25];e-value $2e^{-33}$ YP_002871717
PseBG33_2029		GAGG	55	Yes	-61	a**ggaacg**gcttacgcttatttg**ccac**ata	105	8	0	29	No	Conserved hypothetical protein
PseBG33_2030		GGAG	58	Yes	-96	c**ggaact**gccgtctccctccaa**ccac**aca	301	6	1	20	Yes	Hypothetical protein PFWH6_1944 [*Pseudomonas fluorescens* WH6];e-value $5e^{-59}$ ZP_07774553
PseBG33_2677		AAGGA	58	Yes	-54	a**ggaacc**ctttgggctgatcct**ccac**gca	300	5	0	24	Yes	Putative hemolysin-like protein PFLU1373 [*Pseudomonas fluorescens* SBW25];e-value $1e^{-48}$ YP_002871022.1
PseBG33_2801		AGGA	50	Yes	-64	tt**ggaact**gtcttgtgtaggaata**ccac**tga	452	3	1	28	Yes	Psychrophilic alkaline protease [Pseudomonas Tac Ii18]; e-value 2e-65 1G9K_A
PseBG33_2806		AAGGT	54	Yes	-72	c**ggaacc**tgccccatccaccccc**ccact**ca	1195	8	1	26	Yes	Hypothetical protein PFLU3022 [*Pseudomonas fluorescens* SBW25];e-value $4e^{-116}$ YP_002872602
PseBG33_4215		GGAG	54	Yes	-45	t**ggaacg**cccaacgcccattgcg**ccac**gga	363	5	2	25	Yes	Hypothetical protein PFLU4727 [*Pseudomonas fluorescens* SBW25];e-value $5e^{-106}$ YP_002874248
PseBG33_5141		GAGG	49	Yes	-71	g**ggaacc**gtaggaagaaatacg**ccac**ata	201	5	2	28	No	Hypothetical protein
PseBG33_5228	*exoU*	GAGAGA	58	No			639	9	1	28	Yes	Hypothetical protein PFLU5718 [*Pseudomonas fluorescens* SBW25];e-value 0.0 YP_002875210

doi: 10.1371/journal.pgen.1002784

PseBG33_5533	GAGG	54	Yes	-52	t**ggaact**acatcggtacggattg**acac**ata	327	7	1	32	Yes	Hypothetical protein PFWH6_5178 [*Pseudomonas fluorescens* WH6];e-value $2e^{-06}$ ZP_07777741
**Strain A506**											
PflA506_1197	GGAG	57	Yes	-63	t**ggaacc**atcttcattcgtacg**ccac**aga	118	7	0	24	Yes	Hypothetical protein
PflA506_1198	GGAAGG	51	Yes	-38	t**ggaacc**taattgcgtcaggta**ccac**aaa	61	3	1	26	Yes	Hypothetical protein
PflA506_1213	GAGG	51	Yes	-84	g**ggaacc**tttttttcaggcttg**ccac**aga	79	6	0	21	Yes	Hypothetical protein
PflA506_1910	GGAGG	59	Yes	-37	a**ggaacg**ttttcggtccgtagca**ccac**cta	87	8	1	26	No	Hypothetical protein
PflA506_1911	GGATG	51	Yes	-36	c**ggaacg**gtttcgtcaggcccg**ccac**ata	499	7	0	23	No	Putative hemolysin-like protein PFLU1373 [*Pseudomonas fluorescens* SBW25];e-value $1e^{-79}$ YP_002871022.1
PflA506_1913	GGAGG	53	Yes	-36	a**ggaacg**gcctccacccgtaaca**ccac**ata	575	6	1	30	No	Hemolysin-like protein [*Pseudomonas fluorescens* SBW25];e-value $6e^{-67}$ YP_002871023
PflA506_1996	GGAG	57.3	Yes	-72	a**ggaact**gattccgacgcaaaa**ccac**aca	339	8	1	22	Yes	Hypothetical protein PFWH6_1942 [*Pseudomonas fluorescens* WH6];e-value $3e^{-107}$ ZP_07774551
PflA506_1997	GAGG	59	Yes	-60	a**ggaacg**aattacgcccatttg**ccac**aga	105	10	0	29	Yes	Hypothetical protein
PflA506_1998	GGAG	59	Yes	-97	c**ggaact**gccgagttcctccaa**ccac**aca	305	8	1	25	Yes	Hypothetical protein PFLU2101 [*Pseudomonas fluorescens* SBW25];e-value $1e^{-45}$ YP_002871719
PflA506_2515	GAGG	57	Yes	-46	t**ggaacc**acagggcgagtggcga**ccac**gca	158	10	0	30	No	Hypothetical protein
PflA506_3392	GAGAC	55	Yes	-98	a**ggaact**tttccgaccgcctgg**ccac**gca	376	5	0	26	No	Hypothetical protein PFLU4200 [*Pseudomonas fluorescens* SBW25];e-value $1e^{-16}$ YP_002873748
PflA506_4033	GGAG	55	Yes	-44	t**ggaacg**cccaacgccccttgcg**ccac**gca	365	7	1	24	Yes	Hypothetical protein PFLU4727 [*Pseudomonas fluorescens* SBW25];e-value $9e^{-104}$ YP_002874248
PflA506_4642	GAGG	52	Yes	-38	t**ggaact**ttacctacagcatca**ccac**tca	989	6	0	26	Yes	Dermonecrotic toxin [*Enterocytozoon bieneusi* H348]; e-value $2e^{-108}$ XP_002651020

PflA506_5017	*exoU*	GAGAG	60	No			639	8	1	25	Yes	Hypothetical protein PFLU5718 [*Pseudomonas fluorescens* SBW25];e-value 0.0 YP_002875210
PflA506_5312		GAGG	50.1	Yes	-50	t**ggaact**acatccaaacgacagg**acac**ata	364	4	1	19	Yes	Hypothetical protein PFWH6_5178 [*Pseudomonas fluorescens* WH6];e-value $2e^{-08}$ ZP_07777741
**Strain SS101**												
PflSS101_1235		GGAG	57	Yes	-62	t**ggaacc**atattcgctcgtgcg**ccac**aga	122	7	1	24	Yes	Hypothetical protein
PflSS101_1236		GGAAG	51	Yes	-37	t**ggaacc**caattgcatcaggtg**ccac**aaa	61	4	1	26	Yes	Hypothetical protein
PflSS101_1237		GGAG	50	Yes	-55	g**ggaact**gatagacgtagccga**ccac**tct	168	4	1	26	No	Hypothetical protein
PflSS101_1238		GGAAG	52	Yes	-81	t**ggaact**gctcgcagcgattct**ccac**tta	122	3	0	22	Yes	Hypothetical protein
PflSS101_1257		GAGG	53	Yes	-84	g**ggaacc**tttttttcaggctca**ccac**aga	79	6	0	21	Yes	Hypothetical protein
PflSS101_1272		TGAG	49	Yes	-27	t**ggaacc**aatccccccctcgcttt**ccac**tac	239	7	0	29	Yes	Putative Zn-dependent metalloprotease; myroilysin precursor [*Myroides profundi*]; e-value $3e^{-28}$ gb|ACG59772.1
PflSS101_1872		AGGA	53	Yes	-36	t**ggaacg**gtttcgtcagacaca**ccac**gta	494	8	0	24	No	Putative hemolysin-like protein PFLU1373 [*Pseudomonas fluorescens* SBW25];e-value $9e^{-82}$ YP_002871022.1
PflSS101_1952		GGAG	59	Yes	-75	a**ggaact**gattccgacgcaaaa**ccac**aca	340	8	1	26	Yes	Hypothetical protein [*Pseudomonas fluorescens* SBW25]; e-value $8e^{-40}$ ref|YP_002871717.1
PflSS101_1953		GAGG	59	Yes	-58	a**ggaacg**aatgacgcccgattg**ccac**ata	105	6	0	27	No	Hypothetical protein
PflSS101_1954		GGAG	59	Yes	-97	c**ggaact**gtcgagtccatccaa**ccac**aca	307	5	1	20	Yes	Hypothetical protein PFWH6_1944 [*Pseudomonas fluorescens* WH6];e-value $9e^{-60}$ ZP_07774553
PflSS101_2432		CGAG	58	Yes	-32	t**ggaacc**taccgtcgcgtcttaa**cgac**tca	162	1	0	26	No	Putative T3SS effector Orf30 [*Pseudomonas syringae* pv. *syringae* 642]; e-value $2e^{-06}$ gb|ACU65058.1
PflSS101_2635		AGGTA	55	Yes	-70	**cggaacc**tcgccagaacctcttc**ccac**aca	1175	3	1	21	No	Hypothetical protein PFLU3022 [*Pseudomonas fluorescens* SBW25]; e-value $2e^{-111}$ ref|YP_002872602.1
PflSS101_3399		GAGAC	55	Yes	-97	c**ggaacc**tttccccccgcctgg**ccac**gca	376	6	0	26	No	Hypothetical protein PFLU4200 [*Pseudomonas fluorescens* SBW25]; e-value $3e^{-18}$ ref|YP_002873748.1
PflSS101_4113		GGAG	56	Yes	-44	t**ggaacg**cccaccgcccattgcg**ccac**gta	366	7	1	24	No	Hypothetical protein PFWH6_4508 [*Pseudomonas fluorescens* WH6];e-value $4e^{-168}$ ZP_07777076

doi: 10.1371/journal.pgen.1002784

PflSS101_5066	*exoU*	GAGAG	59	No			639	6	1	27	Yes	Hypothetical protein PFLU5718 [*Pseudomonas fluorescens* SBW25];e-value 0.0 YP_002875210
PflSS101_5366		GAGG	50	Yes	-50	t**ggaact**acatctgtataagaag**acac**aca	427	7	1	26	Yes	Conserved hypothetical protein [*Prochlorococcus marinus* str. NATL2A]; e-value $3e^{-17}$ gb|AAZ58274.1

[a] Strains Pf-5, Pf0-1, and SBW25 were not considered in this analysis
[b] RBS = Ribosome-binding site
[c] Hrp-box is present upstream of *srcM* that encodes RopM-specific chaperone. *srcM* and *ropM* are probably transcribed together.
