# Supplementary material for: Comparative Genomics of Plant-Associated Pseudomonas spp.: Insights into Diversity and Inheritance of Traits Involved in Multitrophic Interactions
Source: PLoS Genet. 2012 Jul 5;8(7):e1002784. doi: 10.1371/journal.pgen.1002784 (PMC3390384; doi:10.1371/journal.pgen.1002784)
Supplement: Table S16 — Phenotypes of strains in the P. fluorescens group and putative genes conferring these phenotypes. The ten strains listed in Table 1 were tested for phenotypes that have been used to classify strains into species of Pseudomonas or biovars of P. fluorescens. Results of the assays are listed as either positive (+), negative (−), or variable (v); controls for each assay are shown. Based on their phenotypes, we assigned the strains to biovars of P. fluorescens according to the scheme presented in Figure S10 or to established species within the P. fluorescens group [31], [130], [131]. Putative genes associated with the tested phenotypes were identified and listed. (PDF) [file pgen.1002784.s026.pdf]

Table S16. Phenotypes of strains in the *Pseudomonas fluorescens* group and putative genes conferring these phenotypes[a]

**Phenotype tested**[b]	**Sub-clade 1**						**Sub-clade 2**						**Sub-clade 3**								**Controls**[k]	
	Pf-5		30-84		O6		Pf0-1		Q8r1-96		Q2-87		SBW25		A506		SS101		BG33R			
	Locus tag[c]	pheno-type	Locus tag	pheno-type	Locus tag	pheno-type	Locus tag	pheno-type	Locus tag	pheno-type	Locus tag	pheno-type	Locus tag	pheno-type	Locus tag	pheno-type	Locus tag	pheno-type	Locus tag	pheno-type	Positive	Negative
Fluorescence[d]	+	+	+	+	+	+	+	+	+	+	+	+	+	+	+	+	+	+	+	+		
Arginine	(****)	+	(****)	+	(****)	+	(****)	+	(****)	+	(****)	+	(****)	+	(****)	+	(****)	+	(****)	+	*P. aeruginosa* PAO1	P. *syringae* B728a
Growth at 41°C	(****)	-	(****)	-	(****)	-	(****)	-	(****)	-	(****)	-	(****)	-	(****)	-	(****)	-	(****)	-	*P. aeruginosa* PAO1	P. *syringae* B728a
Oxidase	(****)	+	(****)	+	(****)	+	(****)	+	(****)	+	(****)	+	(****)	+	(****)	+	(****)	+	(****)	+	*P. aeruginosa* PAO1	P. *syringae* B728a
Tobacco HR	(****)	-	(****)	-	(****)	-	(****)	-	(****)	-	(****)	-	(****)	-	(****)	-	(****)	-	(****)	-	P. *syringae* B728a	water
Gelatinase	3210	+	3127	+	3385	+	2678	+[e]	2858	+	2793	+	3146	+	2617	+	2560	+	2907	+	9446 (chloro)	12633 (Pp Bv A)
Lipase	3202, 3205	+	3119, 3120	+	3377, 3378	+	2686, 2685	+	2864	+	2787	+	3141	-	2625, 2165	+	2567, 2054	+	2900, 2964	+	9446 (chloro), 13985 (aureo)	17467 (bv II)
Phenylacetic acid	3128-3140	+	2806-2790	+	3038-3022	+	------	-	------	-	------	-	------	-	------	-	------	-	------	-	*P. putida* KT2440	*P. aeruginosa PAO1*
Sodium Benzoate	3855	+	3864	+	4156	+	2965	+	2191	+	------	-	------	-	------	-	2539	+	------	-	*P. aeruginosa PAO1*	P. *syringae* B728a
Trehalose	4933-4934	+	4726-4727	+	4974-4975	+	------	-	0992-0991	+	------	-	5039-5040	+	4346-4347	+	4429-4430	+	4537-4538	v	9446 (chloro)	
Levan sucrase	------	-	4797	+	5048	+	------	-	Frame-shift[f]	-	4863	+	2294	+	2166	+	2055	+	------	-	*P. syringae* B728a	*P. aeruginosa* PAO1
L-arabinose	------	-	4536-4538	+	4778-4780	+	4139-4141	+	4099-4101	+	1542-1544	+	2372-2374	+	2250-2252	+	4066-4068	+	4169-4171	+	17467 (bv II)	9446 (chloro)

doi: 10.1371/journal.pgen.1002784

Nitrate reduction[g]	------	-	------	-	3625-3632	+	------	-	3192-3199	+	3067-3074	+	------	-	------	-	------	-	------	-	17467 (bv II), 9446 (chloro), 13985 (aureo)	12633 (Pp Bv A)
D-serine[h]	------	-	2230-2231	+	2461-2462	+	------	-	------	-	------	-	------	-	------	-	------	-	------	-		
Denitrification[g]	------	-	------	-	------	-	------	-	2673-2678	+	2414-2419	+	------	-	------	-	------	-	------	-	17467 (bv II), 17513 (bv IV)	9446 (chloro), 13985 (aureo)
Ethanol[i]	2216	+	------	-	------	-	------	-	2690	+	2431	+	------	-	------	-	------	-	------	-	17467 (bv II)	17513 (bv IV), 13525 (bv I)
Sorbitol	------	-	------	-	------	-	------	-	2706	+	2446	+	2474	+	3106	+	3114	+	2431	+	17513 (bv IV)	9446 (chloro)
Mannitol[h]	3074	+	2850	+	3103	+	2636	+	3133	+	2521	+	2741	+	2955	+	2412	+	3020	+		
myo-Inositol	2591	+	2391	+	2611	+	------	-	3405	+	2121	+	2580	+	3030	+	2928	+	2500	+	*P. syringae* B728a	*P. aeruginosa* PAO1
D-xylose	------	-	------	-	------	-	2303	v	2626	+	2544	+	2301	+	2232	+	2124	+	2336	+	13525 (bv I)	13985 (aureo)
L-tryptophan	0752-0753, 0762	+	0744-0745, 0752	+	0749-0750, 0757	+	------	-	------	-	------	-	5198-5199, 5188	+	4491-4492, 4480	+	4556-4557, 4545	+	4661-4662, 4650	+	17470 (Pp Bv B)	12633 (Pp Bv A)
Adonitol	(****)	-	(****)	-	(****)	-	(****)	-	(****)	-	(****)	-	(****)	+	(****)	+	(****)	+	(****)	+	13525 (bv I), 17559 (bv III)	17467 (bv II)
Biovar or species assigned[j]	*P. protegens* or Biovar V-4		*P. chlororaphis* subsp. *aureofaciens*		*P. chlororaphis* subsp. *aureofaciens*		Biovar V-2		*P. brassicacearum* or Biovar II/IV		Biovar II/IV		Biovar I		Biovar I		Biovar I		Biovar V-6			

[a] +, positive phenotype observed or substrate utilized as sole carbon source; -, negative phenotype observed or no growth on medium with substrate as sole carbon source; v, variable phenotype; ------, gene not present in genome; (****), characterized genes for this phenotype were not identified (may or may not be present). Putative genes that were correlated with phenotypes through comparative genomics are shown, but their roles in conferring these functions were demonstrated only for fluorescence and gelatinase activity.

[b] Genes associated with the phenotypes are based on the characterized proteins referenced below. Gelatinase – Confirmed in this study; Lipase[1,2]; Phenylacetic acid[3]; Sodium Benzoate[4]; Trehalose[5]; Levan Sucrase[6]; L-arabinose (transporter only)[7]; Nitrate reduction[8]; D-serine[9]; Denitrification[8]; Ethanol[10]; Sorbitol[11]; Mannitol[12]; *myo*-Inositol[13]; D-xylose[14]; L-tryptophan[15]; Adonitol – None, based on unique genes and Pfam relations, unconfirmed.

[c] Locus tag: Pf-5, PFL_; 30-84, Pchl3084_; O6, PchlO6_; Pf0-1, PF01_; Q8r1-96, PflQ8_; Q2-87, PflQ2_; SBW25, PFLU; A506, PflA506_; SS101, PflSS101_; BG33R, PseBG33_.

[d] Pyoverdine biosynthesis gene clusters present.

[e] Pf0-1 harboring plasmid pJEL5965 (having *gacA*$^+$ from strain Pf-5 cloned in plasmid pME6000) was positive for gelatinase whereas strain Pf0-1 was negative for gelatinase.

[f] Q8r1-96 did not produce levan from sucrose, which we attribute to a frameshift mutation in the levan sucrase gene in strain Q8r1-96.

[g] Strains O6, Q8r1-96, and Q2-87 reduce nitrate, as indicated by a purple color immediately after adding the nitrite test reagent (24 hours after inoculation of the medium). Strains Q8r1-96 and Q2-87 further reduce the nitrite, as no color is observed after adding the nitrite test regent 48 hour after inoculation of the medium.

[h] Results based on Biolog Phenotype Microarray data; no controls are used.

[i] 100% Ethanol was used at both 0.1% and 1.0% v/v final concentrations; results were the same for both.

[j] Biovar assignments are consistent with the scheme of Stanier *et al.,* [16] and Barrett *et al.,* [17]. Species are assigned according to Peix *et al.,* [18], Ramette *et al.,* [19] and Achouak *et al.,* [20], based upon phenotypic tests described therein and phylogenetic analysis (see Figure S2).

[k] Type strains used as controls are designated by American Type Culture Collection (ATCC) number, followed by the name of the type strain. bv I = *Pseudomonas fluorescens* biovar I, bv II = *Pseudomonas fluorescens* biovar II, bv IV = *Pseudomonas fluorescens* biovar IV, chloro = *Pseudomonas chlororaphis* subsp. *chlororaphis*, aureo = *Pseudomonas chlororaphis* subsp. *aureofaciens*, Pp bv A = *Pseudomonas putida* biovar A, Pp bv B = *Pseudomonas putida* biovar B. No negative control was found for trehalose utilization.

References:

1. Woods RG, Burger M, Beven C-A, Beacham IR (2001) The *aprX-lipA* operon of *Pseudomonas fluorescens* B52: a molecular analysis of metalloprotease and lipase production. Microbiology 147: 345-354.
2. Howard GT, Mackie RI, Cann IKO, Ohene-Adjei S, Aboudehen KS, et al. (2007) Effect of insertional mutations in the *pueA* and *pueB* genes encoding two polyurethanases in *Pseudomonas chlororaphis* contained within a gene cluster. J Appl Microbiol 103: 2074-2083.
3. Teufel R, Mascaraque V, Ismail W, Voss M, Perera J, et al. (2010) Bacterial phenylalanine and phenylacetate catabolic pathway revealed. Proc Natl Acad Sci USA 107: 14390-14395.
4. Jeffrey WH, Cuskey SM, Chapman PJ, Resnick S, Olsen RH (1992) Characterization of *Pseudomonas putida* mutants unable to catabolize benzoate: cloning and characterization of *Pseudomonas* genes involved in benzoate catabolism and isolation of a chromosomal DNA fragment able to substitute for *xylS* in activation of the TOL lower-pathway promoter. J Bacteriol 174: 4986-4996.
5. Matthijs S, Koedam N, Cornelis P, De Greve H (2000) The trehalose operon of Pseudomonas fluorescens ATCC 17400. Res Microbiol 151: 845-851.
6. Hettwer U, Jaeckel FR, Boch J, Meyer M, Rudolph K, et al. (1998) Cloning, Nucleotide Sequence, and Expression in Escherichia coli of Levansucrase Genes from the Plant Pathogens Pseudomonas syringae pv. glycinea and P. syringae pv. phaseolicola. Appl Environ Microbiol 64: 3180-3187.
7. Scripture JB, Voelker C, Miller S, O'Donnell RT, Polgar L, et al. (1987) High-affinity L-arabinose transport operon: nucleotide sequence and analysis of gene products. J Mol Biol 197: 37-46.
8. Zumft W (1997) Cell biology and molecular basis of denitrification. Microbiol Mol Biol Rev 61: 533-616.
9. Marceau M, McFall E, Lewis SD, Shafer JA (1988) D-serine dehydratase from *Escherichia coli*. DNA sequence and identification of catalytically inactive glycine to aspartic acid variants. J Biol Chem 263: 16926-16933.
10. Diehl A, v. Wintzingerode F, Görisch H (1998) Quinoprotein ethanol dehydrogenase of *Pseudomonas aeruginosa* is a homodimer. Eur J Biochem 257: 409-419.
11. Schauder S, Schneider K-H, Giffhorn F (1995) Polyol metabolism of *Rhodobacter sphaeroides*: biochemical characterization of a short-chain sorbitol dehydrogenase. Microbiology 141: 1857-1863.
12. Brünker P, Altenbuchner J, Kulbe KD, Mattes R (1997) Cloning, nucleotide sequence and expression of a mannitol dehydrogenase gene from *Pseudomonas fluorescens* DSM 50106 in *Escherichia coli*. Biochimica et Biophysica Acta (BBA) - Gene Structure and Expression 1351: 157-167.
13. Fujita Y, Shindo K, Miwa Y, Yoshida K (1991) *Bacillus subtilis* inositol dehydrogenase-encoding gene (*idh*): sequence and expression in *Escherichia coli*. Gene 108: 121-125.
14. Liao WX, Earnest L, Kok SL, Jeyaseelan K (1995) Molecular cloning and characterization of the xylose isomerase gene from a thermophilic *Bacillus* species. Biochem Mol Biol Int 36: 401-410.
15. Kurnasov O, Jablonski L, Polanuyer B, Dorrestein P, Begley T, et al. (2003) Aerobic tryptophan degradation pathway in bacteria: novel kynurenine formamidase. FEMS Microbiol Lett 227: 219-227.
16. Stanier RY, Palleroni NJ, Doudoroff M (1966) The aerobic pseudomonads: a taxonomic study. J Gen Microbiol 43: 159-271.
17. Barrett EL, Solanes RE, Tang JS, Palleroni NJ (1986) *Pseudomonas fluorescens* biovar V: its resolution into distinct component groups and the relationship of these groups to other psychotropic *Pseudomonas* associated with food spoilage. J Gen Microbiol 132: 2709-2721.
18. Peix A, Valverde A, Rivas R, Igual JM, Ramírez-Bahena M-H, et al. (2007) Reclassification of *Pseudomonas aurantiaca* as a synonym of *Pseudomonas chlororaphis* and proposal of three subspecies, *P. chlororaphis* subsp. *chlororaphis* subsp. nov., *P. chlororaphis* subsp. *aureofaciens* subsp. nov., comb. nov. and *P. chlororaphis* subsp. *aurantiaca* subsp. nov., comb. nov. Int J Syst Evol Microbiol 57: 1286-1290.
19. Ramette A, Frapolli M, Saux MF-L, Gruffaz C, Meyer J-M, et al. (2011) *Pseudomonas protegens* sp. *nov.*, widespread plant-protecting bacteria producing the biocontrol compounds 2,4-diacetylphloroglucinol and pyoluteorin. Syst Appl Microbiol 34: 180-188.
20. Achouak W, Sutra L, Heulin T, Meyer JM, Fromin N, et al. (2000) *Pseudomonas brassicacearum* sp. *nov*. and *Pseudomonas thivervalensis* sp. *nov.*, two root-associated bacteria isolated from *Brassica napus* and *Arabidopsis thaliana*. Int J Syst Evol Microbiol 50: 9-18.

doi: 10.1371/journal.pgen.1002784
