# Supplementary material for: Comparative Genomics of Plant-Associated Pseudomonas spp.: Insights into Diversity and Inheritance of Traits Involved in Multitrophic Interactions
Source: PLoS Genet. 2012 Jul 5;8(7):e1002784. doi: 10.1371/journal.pgen.1002784 (PMC3390384; doi:10.1371/journal.pgen.1002784)
Supplement: Table S17 — Primers used to construct hcnB, aprA, and chiC mutants in P. protegens Pf-5. (PDF) [file pgen.1002784.s027.pdf]

Table S17. Primers used to construct mutants in *Pseudomonas protegens* Pf-5

Primer	Sequence (5'→3')[a]
hcnB	
hcn UpFnew-Hind	CACAAGGAGCAAGCTTCCACGTTATGAGCCTGAACC
hcn UpRnew-FRT	TCAGAGCGCTTTTGAAGCTAATTCGTCTGCTTGGCGATCTTGCC
hcn DnFnew-FRT	AGGAACTTCAAGATCCCCAATTCGCAGTTGAGCCAGCAGATGG
hcn DnRnew-Hind	GTAGGAGACCAAGCTTCTTAATCATGCTGGGAGACC
aprA	
AprA UpF-Bam	GGAAGTGGATCCTGGCGATGCTCAACAACGAT
AprA UpR	GTTGACGAAGTGGACCGAGAACGAAGGTTTGC
AprA DnF	TCGTTCTCGGTCCACTTCGTCAACGCCTTCAC
AprA DnR-Bam	CAACCTGGATCCGTAACACAATCAGACCGTCG
chiC	
chi UpF-Xba	CCTCTCTCTAGACAAAGCCTTACGACAGCGAT
chi UpR	GGTGAACGCTACCATCAGGGAAGCAGCATCTG
chi DnF	GCTTCCCTGATGGTAGCGTTCACCCTGTGGAG
chi DnR-Xba	CGACACTCTAGAATCAAGGTCAGCATCGGCAG

[a] Restriction sites used for cloning are underlined.

doi: 10.1371/journal.pgen.1002784
